# Schizophrenia risk gene ZNF804A controls ribosome localization and synaptogenesis in developing human neurons

**DOI:** 10.1126/sciadv.aea0755

**Published:** 2026-05-20

**Authors:** Laura Sichlinger, Maximilian Hausherr, Sara Guerrisi, Lucia Dutan-Polit, George Chennell, Roland Nagy, Rugile Matuleviciute, Fatema Nasser, Szidonia Farkas, Rosemary A. Bamford, Szi Kay Leung, Rodrigo R. R. Duarte, Timothy R. Powell, Jonathan Mill, Katrin Marcus, Anthony C. Vernon, Deepak P. Srivastava

**Affiliations:** ^1^ Department of Basic and Clinical Neuroscience, The Maurice Wohl Clinical Neuroscience Institute, Institute of Psychiatry, Psychology & Neuroscience, King’s College London, London, UK.; ^2^ MRC Centre for Neurodevelopmental Disorders, Institute of Psychiatry, Psychology & Neuroscience, King’s College London, London, UK.; ^3^ Medizinisches Proteom-Center, Medical Faculty, Ruhr-University Bochum, 44801 Bochum, Germany.; ^4^ Medical Proteome Analysis, Center for Protein Diagnostics (PRODI), Ruhr-University Bochum, 44801 Bochum, Germany.; ^5^ Department of Clinical and Biomedical Sciences, University of Exeter Medical School, University of Exeter, Exeter, UK.; ^6^ Social, Genetic & Developmental Psychiatry Centre, Institute of Psychiatry, Psychology & Neuroscience, King’s College London, London, UK.

## Abstract

*ZNF804A* was among the first genes robustly associated with schizophrenia based on findings from large-scale genomic studies. Previous research has implicated *ZNF804A* in the regulation of gene expression and synaptic function, but the role of this gene in neurodevelopment and in schizophrenia pathogenesis remains unclear. To study its function during neurodevelopment, we generated isogenic human induced pluripotent stem cells with reduced *ZNF804A* expression, differentiated them into developing cortical glutamatergic neurons, and studied their transcriptomic, synaptic, and protein signatures. Mutant neurons showed modest evidence for changes in gene expression. However, high-content confocal imaging revealed increased excitatory synapse density in mutant neurons. Cell compartment–specific proteomic analysis further revealed that mutant neurons had higher levels of ribosomal and translational proteins within neurites, and high-content imaging confirmed increased local protein synthesis efficiency. Overall, these results demonstrate that in human developing cortical glutamatergic neurons, *ZNF804A* regulates excitatory synapse formation potential via increased local protein translation.

## Teaser

Schizophrenia risk gene ZNF804A regulates local protein synthesis and synapse formation in developing human neurons.

## INTRODUCTION

Heritability estimates for schizophrenia (SZ) are among the highest in psychiatric conditions (*1*). Large-scale genetic screenings have yielded remarkable advances in uncovering the complex polygenic basis of the disorder. It is now understood that both rare genetic variation with high penetrance (*2*, *3*) and common single-nucleotide polymorphisms (SNPs) with low penetrance (*4*) increase susceptibility for SZ. A major challenge remains the translation of information about SZ risk loci identified by genome-wide association studies (GWAS) into neurobiological insights. Evidence from single-cell RNA sequencing (RNA-seq) studies suggests that the expression of multiple SZ risk genes is enriched in glutamatergic neurons present in the developing human cortex (*5*, *6*). This is consistent with evidence from human postmortem (*7*) and neuroimaging (*8*–*10*) studies implicating the glutamatergic system in SZ (*11*). Furthermore, these studies support the view that neurodevelopment is critical, when alterations in neuronal systems, including glutamatergic neurons, may contribute to the emergence of SZ (*12*). To this end, an increasing number of studies have used human induced pluripotent stem cells (hiPSCs) to study the neurobiology of SZ. HiPSCs generated from individuals with SZ and subsequently differentiated toward neuronal fates have identified alterations in neural progenitor cell (NPC) proliferation and synaptic disruptions in developing neurons (*13*–*15*). While studies using patient-derived hiPSCs have enhanced our understanding of the neurobiology of SZ (*16*), they have also highlighted the need for functional genomic studies to dissect how specific genes contribute to disease neurobiology.

Variations within zinc finger protein 804A (*ZNF804A*) have been robustly associated with SZ on a genome-wide scale in large cohorts, establishing it as one of the most robust common risk genes (*4*, *17*, *18*). While neuroimaging studies in both healthy individuals and patients with SZ have linked ZNF804A (dys)function with SZ-related endophenotypes (*19*, *20*), the cellular and molecular characterization of gene function has proven to be challenging. Functional studies have implicated ZNF804A in the maintenance of dendritic spines, the site where the majority of excitatory synapses occur in mature neurons, and in the structural remodeling of dendritic spines in response to activity-dependent stimulation (*21*). The role of ZNF804A in the maintenance of dendritic spines in mature neurons has been subsequently replicated by multiple studies (*22*, *23*). In addition, recent investigations have identified interactions with translation-related proteins for ZNF804A in a yeast two-hybrid screen, the ability to bind mRNA and regulate translational efficiency in rodent cells (*24*, *25*). However, multiple questions remain regarding the precise mechanisms by which ZNF804A functions in neurons, including potential species, cell type, and neurodevelopmental time point–specific differences in its functionality.

To address these gaps, we characterized *ZNF804A* function in human neurons using a functional genomics approach that considers the appropriate time point and cell type in which this gene may confer risk for SZ. Using single-cell datasets of the developing human cortex, we identify developing cortical neurons as the key time point and cell type when *ZNF804A* is most highly expressed. Mutant *ZNF804A* hiPSC cell lines missing exon 3 of *ZNF804A* were generated by CRISPR-Cas9 genome engineering and subsequently differentiated into developing glutamatergic cortical neurons. Bulk RNA-seq revealed modest differential gene expression in mutant *ZNF804A* neurons, suggesting that transcriptional regulation is not a key function of this gene in developing neurons. Nevertheless, genes showing altered expression were enriched for cell adhesion and synaptic functioning, partially supporting previous findings (*26*, *27*). High-content confocal imaging further demonstrated that mutant developing glutamatergic neurons had increased pre- and postsynaptic protein expression along *ZNF804A* mutant neurites and enhanced putative synapse density. Proteomic analysis of neuronal subcompartments indicated altered distribution of translational machinery and translation regulation between neurites and cell somata of mutant ZNF804A neurons. Consistent with these findings, we observed elevated local protein synthesis efficiency and increased expression of ribosomal protein S6 along MAP2-positive (MAP2^+^) neurites in ZNF804A mutant neurons. Overall, this study provides insights into ZNF804A’s role in developing glutamatergic neurons, revealing its involvement in local protein synthesis and synapse formation. These findings establish a potential link between the known cellular functions of *ZNF804A*, while accounting for cell type and developmental time point specificity.

## RESULTS

### Developing glutamatergic cortical neurons are a suitable model to study ZNF804A function

**Fig. 1.**
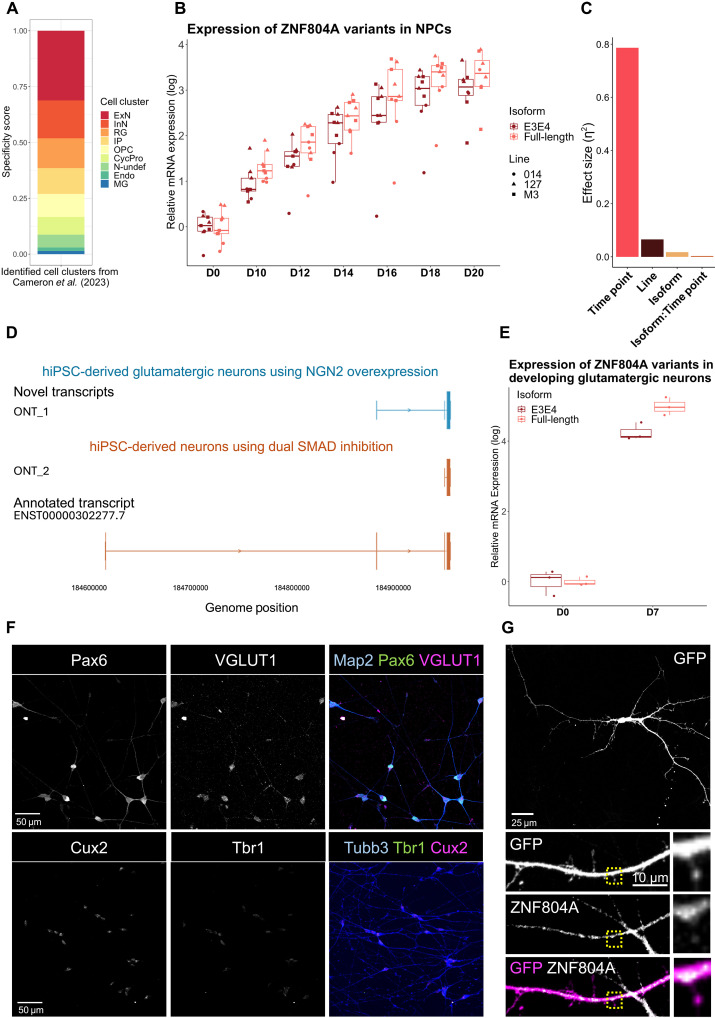
Developing glutamatergic forebrain neurons are a suitable model to study ZNF804A function. (**A**) Stacked bar chart of gene specificity scores for *ZNF804A* in cell population clusters of the fetal cortex (*5*). ExN, developing excitatory neuron; InN, developing inhibitory neuron; N-undef, neuron of undefined class; IP, intermediate progenitor; RG, radial glia; CycPro, cycling progenitor cells; OPC, oligodendrocyte precursor cell; Endo, endothelial cell; MG, microglia. (**B**) Boxplots showing RT-qPCR–derived log10-transformed 2^–∆∆Ct^ values of *ZNF804A^full-length^* and *ZNF804A^E3E4^* at D0 to D20 time points reflecting microstates of NPC development. Overlaying dot plots show values for each cell line. Two-way ANOVA revealed significant results for time point [*F*(6) = 111.3, *P* < 0.0001], isoform [*F*(1) = 15.2, *P* < 0.001], and line [*F*(2) = 27.7, *P* < 0.00001]. (**C**) Effect sizes (η^2^) of two-way ANOVA. (**D**) Oxford Nanopore (ON) long-read sequencing results for transcript isoforms in *ZNF804A* in hiPSC-derived glutamatergic neurons using NGN2 overexpression (blue) or in hiPSC-derived neurons using dual SMAD inhibition (orange). Novel transcripts are annotated with ONT (ON Transcript). (**E**) Boxplots showing RT-qPCR–derived log10-transformed 2^–∆∆Ct^ values of *ZNF804A^full-length^* and *ZNF804A^E3E4^* in D7 forebrain neurons. Overlaying dot plot shows values for each biological replicate. Student’s *t* test revealed reductions for *ZNF804A^E3E4^* at D7 [*t*(4) = −3.58, *P* < 0.05]. (**F**) Representative confocal image of D7 glutamatergic forebrain neurons immunostained for pan-neuronal marker microtubule-associated protein 2 (MAP2; blue, top), excitatory presynaptic marker vesicular glutamate transporter 1 (VGLUT1; magenta, top), paired box protein Pax-6 (Pax6; green, top), pan-neuronal early-stage class III beta-tubulin (Tubb3; blue, bottom), cortical layer VI-specific marker T-box, brain, 1 (Tbr1; green, bottom), and upper layer-specific marker cut-like homeobox 2 (Cux2; magenta, bottom). (**G**) Representative confocal image of green fluorescent protein (GFP)–transfected D7 neuron demonstrating ZNF804A immunostaining on the shaft of dendritic spines.

Previous studies have shown that *ZNF804A* is highly expressed in the brain during the second trimester of human neurodevelopment (*28*, *29*), but we lack information on both cell type and isoform specificity. Hence, we first extracted gene specificity scores for *ZNF804A* expression from published single-nuclei RNA-seq data obtained from the frontal cortices of three fetuses during the second trimester of gestation (*5*). While *ZNF804A* transcripts were broadly distributed across all identified cell populations, there was a notable preference for higher specificity in neuronal cells, particularly within excitatory neurons (specificity score 31%; Fig. 1A). Next, to account for isoform specificity, primers were designed and validated to target the SZ risk variant *ZNF804A^E3E4^* (*29*) and full-length transcripts (table S1). We used reverse transcription quantitative polymerase chain reaction (RT-qPCR) to assess mRNA expression in hiPSCs and six different microstates of cortical NPC development—days 10 to 20 (D10 to D20)—generated using three clones from three donor lines. We confirmed NPC cell fate by expression of markers specific for each state of development (fig. S1, A to C). Transcript expression of each isoform was analyzed relative to mRNA abundance of three housekeeping genes (*GAPDH*, *RPL27*, and *SDHA*) at each time point and relative to mRNA abundance of the respective isoform expression at D0 across lines. Three-way analysis of variance (ANOVA) showed that developmental time point [*F*(6) = 111.3, *P* < 0.0001, η^2^ = 0.786], isoform [*F*(1) = 15.2, *P* < 0.001, η^2^ = 0.018], and individual genetic makeup (i.e., different donor lines) [*F*(2) = 27.7, *P* < 0.00001, η^2^ = 0.065] had a statistically significant main effect on transcript abundance (table S2 and Fig. 1, B and C). Tukey post hoc analysis revealed that *ZNF804A^E3E4^* expression was significantly lower than the full-length variant throughout NPC development (*P*_adj_ = 0.0002). Moreover, Tukey post hoc analysis showed significant differences when comparing almost all time points with higher expression in later stages of development (table S2 and Fig. 1B). Comparisons of later developmental stages did not yield statistically significant results [D14 versus D16 (*P*_adj_ = 0.15), D16 versus D18 (*P*_adj_ = 0.12), and D18 versus D20 (*P*_adj_ = 0.99); table S2 and Fig. 1B]. Next, we characterized the full spectrum of *ZNF804A* isoforms expressed in developing neurons. We conducted targeted Oxford Nanopore Technologies (ONT) long-read sequencing for full-length transcript detection at neuronal time points using different differentiation protocols, namely, cortical neurons generated via dual SMAD inhibition, or via overexpression of *NEUROGENIN2* (*NGN2*). Apart from the annotated *ZNF804A* transcript (*ENST00000302277.7*), we detected two previously unidentified truncated transcripts only expressing exon 4 in neurons from both differentiations (ONT_1 and ONT_2; Fig. 1D). These transcripts likely represent *ZNF804A^E3E4^*, which is encoded purely by exon 4 (*29*). Overall, these data show that *ZNF804A* expression gradually increases throughout cortical NPC development, with its highest levels reached in developing glutamatergic neurons and underlines the importance of taking isoform specificity into account for functional analysis.

To study ZNF804A function in glutamatergic neurons, we opted to use a forward programming approach to rapidly generate this cell type (*30*, *31*). To avoid cell-type variability and enable more specific assumptions about *ZNF804A* functionality, *NGN2* overexpression was combined with a directed differentiation approach using SMAD and Wnt inhibition (*32*). This approach has been reported to yield developing upper-layer cortical glutamatergic neurons. This cell population exhibits an enrichment for SZ risk variants (*5*) and pronounced *ZNF804A* expression (Fig. 1A). On D7 of differentiation in this protocol, glutamatergic neurons were confirmed to express upper-layer cell identity markers including cut-like homeobox 2 (Cux2) transcripts (fig. S1, D to F) and protein (Fig. 1F). Furthermore, neurons highly expressed vesicular glutamate transporter 1 (VGLUT1), confirming their glutamatergic fate (Fig. 1F). Next, we tested isoform-specific *ZNF804A* expression using RT-qPCR as above. The results showed a substantial increase in transcript abundance for both isoforms on D7 and *ZNF804A^E3E4^* levels were significantly lower than full-length transcripts [(mean ± SD) *ZNF804A^E3E4^* (4.25 ± 0.25) versus *ZNF804A^full-length^* (4.98 ± 0.26), *t*(4) = −3.58, *P* < 0.05], confirming expression profiles as observed in NPCs (Fig. 1E), and further supporting observations from our long-read sequencing data (Fig. 1D). Last, we confirmed ZNF804A protein expression in D7 glutamatergic neurons by confocal microscopy, with protein expression observed along neurites and at the base of putative dendritic spines (Fig. 1G). Together, these data demonstrate that developing glutamatergic neurons are a suitable model system for studying ZNF804A neurobiological function.

### Generation of mutant ZNF804A hiPSC lines

**Fig. 2.**
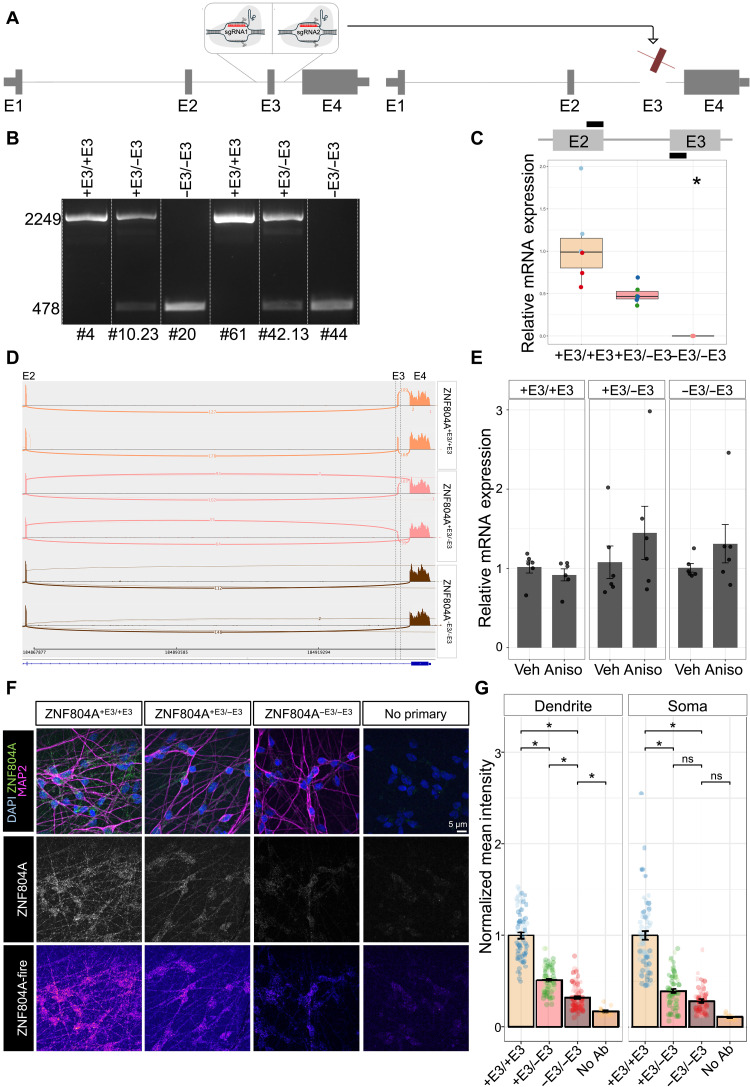
A cellular ZNF804A loss-of-function model. (**A**) Schematic representation of dual–single guide RNA (sgRNA) CRISPR-Cas9 approach to generate ZNF804A loss-of-function model. Created in BioRender. Powell, T. (2026) https://BioRender.com/vh5vr9x. (**B**) Representative PCR blot of ZNF804A^+E3/+E3^, ZNF804A^+E3/−E3^, and ZNF804A^−E3/−E3^ clones. Primers spanned the excision site amplifying either 2249 nucleotide (nt; ZNF804A^+E3/+E3^, ZNF804A^+E3/−E3^) or 478 nt (ZNF804A^−E3/−E3^, ZNF804A^+E3/−E3^) sequences. Clone IDs: wild type: ZNF804A^+E3/+E3^_4, ZNF804A^+E3/+E3^_61; heterozygous exon 3 deletion: ZNF804A^+E3/−E3^_10.23, ZNF804A^+E3/−E3^_42.13; homozygous exon 3 deletion: ZNF804A^−E3/−E3^_44, ZNF804A^−E3/−E3^_20. (**C**) Boxplots of RT-qPCR–derived log10-transformed 2^–∆∆Ct^ values of *ZNF804A^E2E3^* in D7 neurons derived from ZNF804A^+E3/+E3^, ZNF804A^+E3/−E3^, and ZNF804A^−E3/−E3^ lines (*n* = 18; three differentiations per clone). Expression in individual clones is depicted in overlapping dot plots. *P_adj_ < 0.05. (**D**) Representative Sashimi blots showing alternative splicing events in the mutant ZNF804A^+E3/−E3^ and ZNF804A^−E3/−E3^ lines skipping exon 3, but not in wild-type ZNF804A^+E3/+E3^ neurons. (**E**) Bar charts of RT-qPCR results of fusion exon 2 to 4 amplification in D7 neurons from ZNF804A^+E3/+E3^, ZNF804A^+E3/−E3^, and ZNF804A^−E3/−E3^ lines (*n* = 18; three differentiations per clone) treated with 40 μM of nonsense-mediated decay (NMD) inhibitor anisomycin or vehicle control. (**F**) Representative confocal images of D7 neurons derived from ZNF804A^+E3/+E3^, ZNF804A^+E3/−E3^, and ZNF804A^−E3/−E3^ lines immunostaining for pan-neuronal marker microtubule-associated protein 2 (MAP2; magenta), 4′,6-diamidin-2-phenylindol (DAPI; blue), and ZNF804A (green). No primary antibody control images are shown on the right. (**G**) Bar charts showing quantification of normalized mean intensity of ZNF804A staining along MAP2^+^ dendrites [left; one-way ANOVA: *F*(3, 234) = 202.4, *P* < 2 × 10^−16^] or somata [right; one-way ANOVA: *F*(3, 224) = 102.6, *P* < 2 × 10^−16^] of ZNF804A^+E3/+E3^, ZNF804A^+E3/−E3^, or ZNF804A^−E3/−E3^ neurons. Tukey HSD post hoc tests revealed a significant reduction of protein in ZNF804A^−E3/−E3^ (dendrite and soma: *P*_adj_ < 0.0001) and ZNF804A^+E3/−E3^ (dendrite and soma: *P*_adj_ < 0.0001) mutants compared to ZNF804A^+E3/+E3^. Error bars: mean ± SEM, *P_adj_ < 0.05. ns, not significant.

To study the function of ZNF804A, we used a dual–single guide RNA (sgRNA) CRISPR-Cas9 approach to disrupt the function of the gene. Specifically, we targeted exon 3 for complete ablation due to its presence in all known *ZNF804A* transcripts (*33*) (Fig. 2A and fig. S2A). sgRNAs were designed to induce double-strand breaks within intron 2 (sgRNA1) and intron 3 (sgRNA2), resulting in the excision of a 1783–base pair (bp) genomic fragment, including exon 3 (fig. S2A). This deletion was predicted to introduce a frameshift and premature stop codons in exon 4, effectively disrupting downstream translation (fig. S2B). We selected six single-cell clones for downstream analysis: 2× heterozygous exon 3 deletion (het—ZNF804A^+E3/−E3-^) clones (#10.23 and #42.13), 2× homozygous exon 3 deletion (hom—ZNF804A^−E3/−E3^) clones (#20 and #44), and 2× clones with no edits to exon 3 (wild type—ZNF804A^+E3/+E3^) (#4 and #61) (Fig. 2B). Sanger sequencing confirmed the expected and identical deletion boundaries (chr2: +184933496 and +184935280) in ZNF804A^+E3/−E3^ and ZNF804A^−E3/−E3^ mutant clones, while wild-type ZNF804A^+E3/+E3^ clones showed unaltered sequences up- and downstream of each putative Cas9 cut site (fig. S2C). Furthermore, we confirmed the integrity and ruled out off-target effects in all clones using RT-qPCR, immunocytochemistry (ICC), and Sanger sequencing (fig. S3, A to C; table S1; and Supplementary Text).

To assess the impact of this deletion on gene expression, we evaluated *ZNF804A* mRNA transcript and protein expression in D7 developing glutamatergic neurons. RT-qPCR confirmed exon 3 deletion in ZNF804A^+E3/−E3^ and ZNF804A^−E3/−E3^ mRNA transcripts, revealing a significant reduction in transcript abundance across both full-length [Kruskal-Wallis: χ^2^(2) = 14.79, *P* < 0.001] and *ZNF804A^E3E4^* [Kruskal-Wallis: χ^2^(2) = 15.16, *P* < 0.001] isoforms across all groups (Fig. 2C and fig. S2D). Dunn’s post hoc tests with Holm corrections indicated that these effects were most pronounced in ZNF804A^−E3/−E3^ mutants (ZNF804A^E2E3^: *P*_adj_ < 0.01, *ZNF804A^E3E4^*: *P*_adj_ < 0.001). To further characterize the effects of exon 3 deletion, we performed bulk RNA-seq on each of the six clones independently differentiated three times until D7 (*n* = 18).

To analyze the potential impact of *ZNF804A* mutation on neuronal differentiation, we assessed cell fate acquisition by analyzing relevant gene expression (*32*) using log-transformed DESeq2 normalized counts (fig. S3D). The results confirmed the generation of developing glutamatergic cells, aligning with the differentiation protocol (*32*). The expression of cell fate markers supports an upper-layer cortical neuron identity. These expression patterns were consistent across all clones and genotypes, indicating that *ZNF804A* mutation did not interfere with cell fate acquisition (fig. S3D).

Inspection of aligned RNA-seq reads confirmed exon 3 excision in ZNF804A^+E3/−E3^ and ZNF804A^−E3/−E3^ neurons (fig. S4). However, exon 4 appeared to be transcribed in mutation clones (fig. S4). We next analyzed alternative splicing events in ZNF804A^+E3/−E3^ and ZNF804A^−E3/−E3^ neurons compared to wild-type ZNF804A^+E3/+E3^ neurons. This identified a unique alternative splicing event in the mutant ZNF804A^+E3/−E3^ and ZNF804A^−E3/−E3^ lines where exon 3 was skipped, resulting in a previously unidentified exon 2–exon 4 fusion transcript. In contrast, exon 2–exon 4 fusion transcripts were absent in wild-type ZNF804A^+E3/+E3^ neurons (Fig. 2D). Consistent with these findings, RT-qPCR analysis of cDNA from D7 ZNF804A^+E3/+E3^, ZNF804A^+E3/−E3^, and ZNF804A^−E3/−E3^ neurons using primers spanning exon 2 to exon 4, confirmed the presence of a previously unidentified exon 2–exon 4 fusion transcript only in mutant lines (fig. S4, A to F).

To determine whether this transcript could encode a truncated protein, we performed Sanger sequencing of cDNA from D7 homozygous (*ZNF804A^−E3/−E3^*) neurons. This confirmed the presence of an exon 2–exon 4 fusion amplicon and sequence consistent with two fusion transcript variants (fig. S5, E to G; annotated as isoform 1 and isoform 2). In silico analysis of the open reading frame of the exon 2–exon 4 fusion sequence identified two potential coding scenarios: (i) translation initiating at the canonical start codon (exon 1) and terminating prematurely shortly after the beginning of exon 4, and (ii) a putative internal initiation site near the 3′ end of exon 2 that also terminates prematurely within exon 4 variants (fig. S5G). In all scenarios, a premature terminal codon was identified within exon 4, indicating that the exon 2–exon 4 fusion transcript is unlikely to generate a stable truncated proteoform, and that the exon 2–exon 4 fusion transcript would be targeted for nonsense-mediated decay (NMD).

We next directly tested whether the exon 2–exon 4 fusion transcript was sensitive to NMD. D7 neurons from ZNF804A^+E3/+E3^, ZNF804A^+E3/−E3^, and ZNF804A^−E3/−E3^ lines were treated with either the NMD inhibitor anisomycin (40 μM) or vehicle control for 6 hours before RNA extraction. RT-qPCR with exon 2 to 4 spanning primers revealed an increase in the exon 2–exon 4 fusion transcript expression only in ZNF804A^+E3/−E3^ and ZNF804A^−E3/−E3^ treated with anisomycin (Fig. 2E). Although groupwise comparisons did not reach statistical significance, estimation statistics revealed robust genotype-dependent positive shifts in exon 2 to 4 transcript expression following NMD inhibition, as demonstrated by both increased group means and positive bootstrapped mean differences (fig. S5H). Collectively, these findings support the interpretation that the exon 2–exon 4 fusion transcript generated following exon 3 excision is subject to NMD and unlikely to give rise to a stable translated product.

To determine whether exon 3 excision alters ZNF804A protein levels, we quantified ZNF804A expression by ICC and confocal microscopy in D7 neurons of each genotype. Analysis of mean fluorescence intensity of ZNF804A staining along MAP2^+^ neurites and somata revealed a gene dosage–dependent reduction of ZNF804A expression intensity along neurites [one-way ANOVA: *F*(3,234) = 202.4, *P* < 2 × 10^−16^] and somata [one-way ANOVA: *F*(3,224) = 102.6, *P* < 2 × 10^−16^] in ZNF804A^+E3/−E3^ and ZNF804A^−E3/−E3^ neurons (Fig. 2, F and G). Together, exon 3 excision results in a robust gene dose–dependent reduction in ZNF804A protein expression. Although we cannot formally exclude the existence of low-abundance truncated proteoforms encoded by the exon 2–exon 4 fusion transcript, converging evidence from transcript sequencing, open-reading-frame analysis, NMD sensitivity, and protein quantification strongly argues against the presence of a stable or biologically relevant truncated ZNF804A proteoform. Thus, the exon 3 excision model represents a biologically relevant reduction-of-function system for investigating the cellular consequences of diminished ZNF804A expression.

Together, these findings demonstrate that deletion of exon 3 leads to the generation of an unstable exon 2–exon 4 fusion transcript that undergoes NMD and produces a gene dosage–dependent reduction in ZNF804A protein expression, consistent with a loss-of-function ZNF804A neuronal model.

### Transcriptomic analysis of mutant neurons reveals modest effects on gene expression

Previous functional studies have implicated ZNF804A in the regulation of gene transcription (*26*, *27*, *34*). To test whether disruption of ZNF804A function results in significant transcriptome-wide gene expression differences, we conducted an RNA-seq analysis of mutant developing glutamatergic neurons. Each of the six clones was independently differentiated three times as above.

**Fig. 3.**
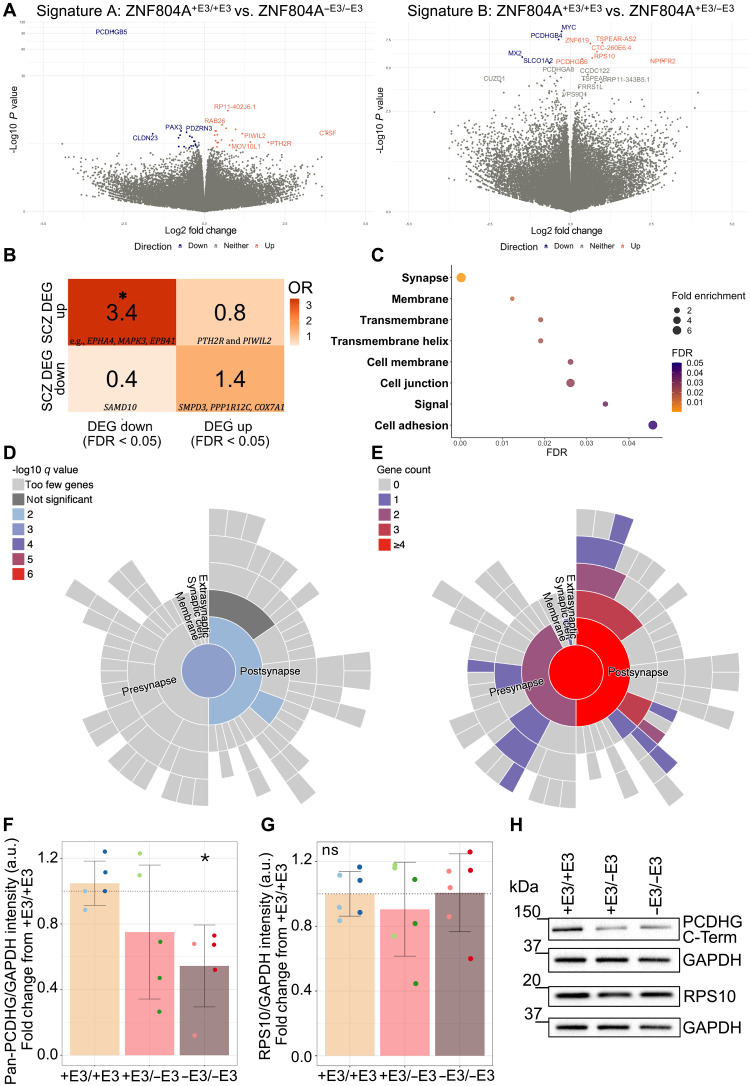
Transcriptomic effects of ZNF804A loss-of-function show disruptions in genes associated with cell adhesion and the glutamatergic synapse. (**A**) Volcano plots depicting differentially expressed genes (DEGs) in signatures A (left) and B (right). The *x* axis represents log2FC, and the *y* axis denotes −log10 *P* value. Up-regulated DEGs (FDR < 0.05) are indicated in red, and down-regulated DEGs are denoted in blue. (**B**) Heatmap illustrating Fisher’s exact test results. The color key represents odds ratios (dark orange = high; light orange = low). An asterisk denotes significance (FDR < 0.05) for the gene set overlap analysis between the down-regulated gene set of signature A and the up-regulated gene set from postmortem schizophrenia brain tissue (*35*). Examples of overlapping genes are shown in the squares of their respective comparison sets. *FDR < 0.05. (**C**) Gene ontology (GO) analysis of DEGs in signature A (FDR < 0.05). The *x* axis displays FDR, and the *y* axis lists terms with significant enrichment (FDR < 0.05). Icon size represents fold enrichment. (**D**) Sunburst blot of Log10 *q* value as analyzed by SynGO showing enrichment in genes differentially expressed in ZNF804A^−E3/−E3^ neurons (FDR < 0.05) relative to a background set of all genes expressed in dataset. (**E**) Sunburst blot of gene counts enriched in synaptic ontology terms analyzed by SynGO. (**F**) Bar chart showing the quantification of integrated densities for pan-PCDHG band (~120 kDa). Overlapping dot plots display pan-PCDHG clone intensities. One-way ANOVA [*F*(2,12) = 3.89, *P* = 0.0498] with subsequent Tukey HSD post hoc tests revealed significant reductions of pan-PCDHG in ZNF804A^−E3/−E3^. (**G**) Bar chart showing no change in ribosomal protein S10 (RPS10; ~19 kDa) expression [*F*(2,15) = 0.36, *P* = 0.701]. (**H**) Representative Western blot showing reduced pan-gamma protocadherin (PCDHG) protein in ZNF804A^−E3/−E3^ lines and no change in ribosomal protein S10 (RPS10; ~19 kDa) expression. Housekeeping protein glyceraldehyde-3-phosphate dehydrogenase (GAPDH) served as loading control. Error bars: mean ± SD, *P_adj_ < 0.05.

We then examined transcriptomic variations caused by mutated ZNF804A by comparing ZNF804A^−E3/−E3^ and ZNF804A^+E3/−E3^ mutation genotypes with ZNF804A^+E3/+E3^ cells. Initial principal components analysis (PCA) of the gene expression data indicated some limited sample clustering, which may contradict a potential role as a regulator of gene transcription. Notably, samples with ZNF804A^−E3/−E3^ and ZNF804A^+E3/+E3^ genotypes displayed a tendency to cluster together based on their genotype, while no noticeable clustering was observed for the clone variable (fig. S7A). To comprehensively evaluate the effect of exon 3 excision on gene expression and therefore ZNF804A transcriptional function, we used three different gene expression comparison signatures, each analyzed using DESeq2. All differentially expressed genes (DEGs) of each signature are provided in table S3. Signature A (“ZNF804A^+E3/+E3^ versus ZNF804A^−E3/−E3^”) comparisons yielded a total of 45 DEGs with 23 genes down- and 22 genes up-regulated (Fig. 3A) and top 20 protein-coding DEGs (10 up- and 10 down-regulated) clustered according to genotype (fig. S7B). Signature B (“ZNF804A^+E3/+E3^ versus ZNF804A^+E3/−E3^”) had fewer DEGs than signature A, which is expected due to the milder, mono-allelic genomic effects of *ZNF804A* exon 3 excision. Nevertheless, four genes were significantly down-regulated and six genes were up-regulated in heterozygous compared to wild-type conditions [false discovery rate (FDR) < 5%] (Fig. 3A). The heatmap of all 10 DEGs generally showed clustering by genotype, except for replicate 2 of ZNF804A^+E3/+E3^_4, which appeared more similar to ZNF804A^+E3/−E3^ clones (fig. S7C). Last, comparisons in signature C (“ZNF804A^+E3/−E3^ versus ZNF804A^−E3/−E3^”) yielded the highest number of DEGs (47 genes, FDR < 5%), with only 7 genes down-regulated and 35 genes up-regulated (fig. S7D). Heatmap clustering of the top 20 protein-coding DEGs (10 up-regulated and 10 down-regulated) confirmed the genotype-based impact on gene expression, as ZNF804A^−E3/−E3^ and ZNF804A^+E3/−E3^ clones clustered according to their genotype (fig. S7E).

Next, we sought to corroborate the association between *ZNF804A* and SZ by overlap analysis of DEGs from signature A with DEGs measured in postmortem brain tissue obtained from patients with SZ (*35*). Fisher’s exact test on two sets of DEGs (up- and down-regulated) from signature A versus two sets of DEGs from postmortem SZ brain tissue samples against a physiologically relevant background (four comparisons in total) revealed a significant overlap between down-regulated genes from signature A and up-regulated genes found in SZ postmortem samples [n genes in signature A down-regulated = 24, n genes in cases = 2274, overlap size = 7 genes, *P* = 0.011, odds ratio (OR = 3.4, FDR < 5%; Fig. 3B]. Gene ontology (GO) analysis of the overlapping genes did not yield significant terms at a 5% FDR threshold, but six of the seven DEGs matched a “Membrane” gene list that included *EPHA4*, *MAPK3*, and *EPB41*.

To better understand how identified DEGs may converge on shared biological or molecular processes, we investigated associations between gene products and GO terms using the Database for Annotation, Visualization, and Integrated Discovery (DAVID) (*36*). We subjected DEGs with corrected *P* values <0.07 to analysis against a predefined biologically relevant context, which included all genes expressed in the samples. Across functional annotations (including biological process, cellular component, and molecular function) and GO, terms including “Synapse” [Fold enrichment (FE) = 7.80, *P* < 0.001, FDR = 0.0002], “Cell Junction” (FE = 5.3, *P* < 0.01, FDR = 0.03), “Cell adhesion” (FE = 6.60, *P* < 0.01, FDR = 0.045), and “Signal” (FE = 1.8, *P* < 0.01, FDR = 0.03) were significantly associated with the DEG set (Fig. 3C and table S4), consistent with previous transcriptomic analyses of ZNF804A function in human cell models (*26*, *27*). While each signature identified unique DEGs, there were also substantial overlaps between the gene sets (fig. S7F). Notably, genes related to cell adhesion proteins, including protocadherins {e.g., *PCDHB8* [ZNF804A^+E3/+E3^ versus ZNF804A^−E3/−E3^: log2 fold change (log2FC) = −0.4, *P* < 0.001, FDR = q < 0.03; ZNF804A^+E3/+E3^ versus ZNF804A^+E3/−E3^: log2FC = −0.2, *P* < 0.05, FDR q > 0.05], *PCDHGB4* (ZNF804A^+E3/+E3^ versus ZNF804A^+E3/+E3^: log2FC = 0.3, *P* < 0.001, FDR q < 0.001; ZNF804A^+E3/+E3^ versus ZNF804A^+E3/−E3^: log2FC = −0.3, *P* < 0.001, FDR q < 0.001), and *PCDHGB6* (ZNF804A^+E3/+E3^ versus ZNF804A^−E3/−E3^: log2FC = −0.1, *P* > 0.05, FDR q > 0.05; ZNF804A^+E3/+E3^ versus ZNF804A^+E3/−E3^: log2FC = 0.4, *P* < 0.001, FDR q = 0.005)} and glutamatergic synapse genes [e.g., *SHANK2* (ZNF804A^+E3/+E3^ versus ZNF804A^−E3/−E3^: log2FC = 0.4, *P* < 0.001, FDR q = 0.002; ZNF804A^+E3/+E3^ versus ZNF804A^+E3/+E3^: log2FC = 0.2, *P* < 0.01, FDR q > 0.05)], exhibited dysregulation across all signatures, supporting the role of ZNF804A in synaptic regulation and formation (Fig. 3C). In line with this, synaptic ontology terms assessed by SynGO showed enrichment in genes differentially expressed due to homozygous *ZNF804A* exon 3 excision (FDR < 7%) relative to a background set of all genes expressed in our dataset (Fig. 3, D and E). “Biological Process ontology terms” significantly associated with the DEG set included synapse organization (*q* = 2.31 × 10^−4^, *P* = 7.71 × 10^−5^, gene count = 7), process in the synapse (*q* = 3.29 × 10^−4^, *P* = 2.19 × 10^−4^, gene count = 10), and regulation of synapse assembly (*q* = 2.50 × 10^−3^, *P* = 2.50 × 10^−3^, gene count = 3). “Cellular Component ontology terms” significantly enriched in the DEG set included postsynapse (*q* = 7.42 × 10^−3^, *P* = 5.56 × 10^−3^, gene count = 7) and postsynaptic membrane (*q* = 7.42 × 10^−3^, *P* = 4.14 × 10^−3^, gene count = 3), whereas presynaptic terms were not significantly enriched (Fig. 3, D and E).

Overall, our data and the existing literature (*26*, *27*) support the assumption that ZNF804A exon 3 excision yields transcriptomic disruptions related to the postsynapse and cell adhesion. However, the extent of this disruption at the protein level remains unclear. Thus, we assessed gamma protocadherin (PCDHG) protein expression in developing glutamatergic cortical neurons derived from ZNF804A^+E3/+E3^ and mutant cells. Pan-PCDHG protein levels were significantly reduced in ZNF804A^−E3/−E3^ cell lysates compared to ZNF804A^+E3/+E3^ controls (Fig. 3, F and H). Conversely, ZNF804A^+E3/−E3^ mutant neurons did not have a statistically significant effect on protein abundance compared to wild-type cells [one-way ANOVA: *F*(2,12) = 3.89, *P* = 0.0498; Tukey honestly significant difference (HSD): ZNF804A^+E3/+E3^-ZNF804A^−E3/−E3^: *P*_adj_ < 0.05, all other comparisons *P*_adj_ > 0.05; mean FC from wt: ZNF804A^−E3/−E3^ = 0.5, het = 0.3, Fig. 3F]. On the basis of previous research linking disruptions in protein synthesis to ZNF804A function (*25*) and results of the RNA-seq analysis yielding differential expression for ribosomal protein S10 (RPS10) in ZNF804A^+E3/−E3^ conditions (log2FC = 0.7, *P* < 0.001, FDR = 0.005), we investigated the impact of *ZNF804A* exon 3 excision on RPS10 protein expression as above, but found no statistically significant effect of genotype [*F*(2,15) = 0.36, *P* = 0.701; Fig. 3, G and H]. Together, transcriptomic analysis of ZNF804A exon 3 excised neurons revealed only modest effects on gene expression. However, the genes showing differential expression include those associated with postsynapse, cell adhesion, and SZ pathology as previously reported (*26*, *27*, *29*, *34*).

### Increased synapse number along dendrites of ZNF804A mutant neurons

**Fig. 4.**
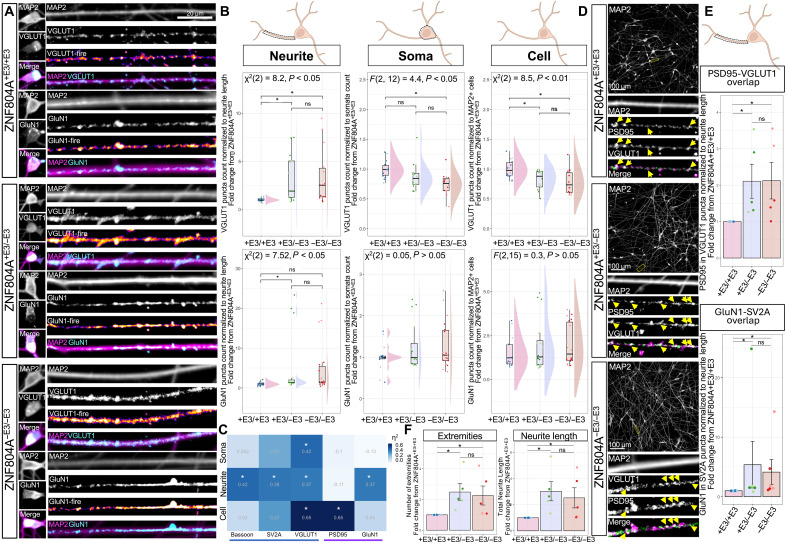
ZNF804A-deficient neurites show increased overlap of pre- and postsynaptic proteins. (**A**) Representative confocal images of D7 ZNF804A^+E3/+E3^, ZNF804A^+E3/−E3^, and ZNF804A^−E3/−E3^ neurons immunostained for microtubule-associated protein 2 (MAP2; magenta), NMDA receptor subunit 1, and vesicular glutamate transporter 1 (GluN1, VGLUT1; fire LUT and cyan) in neurites and somata. (**B**) Raincloud plots quantifying VGLUT1 and GluN1 puncta in compartments of MAP2^+^ neurons. Overlaying dot plots show technical replicates. VGLUT1 puncta were decreased in whole-cell regions [Kruskal-Wallis: χ^2^(2) = 8.5, *P* = 0.007] of ZNF804A^+E3/−E3^ and ZNF804A^−E3/−E3^ neurons, and in somata [one-way ANOVA: *F*(2,12) = 4.5, *P* = 0.03] of ZNF804A^−E3/−E3^ neurons. VGLUT1 puncta were increased along MAP2^+^ neurites of ZNF804A^+E3/−E3^ and ZNF804A^−E3/−E3^ neurons [Kruskal-Wallis: χ^2^(2) = 6.4, *P* = 0.04, *n* = 15]. GluN1 puncta were increased in MAP2^+^ neurites of ZNF804A^+E3/−E3^ neurons [Kruskal-Wallis: χ^2^(2) = 7.52, *P* = 0.01, *n* = 18]. (**C**) Heatmap illustrating effect sizes (η^2^) of ZNF804A mutation on pre- and postsynaptic proteins across compartments. Magnitude shown in blue. (**D**) Representative confocal image of colocalized VGLUT1 and PSD95 puncta in D7 neurons immunostained for MAP2 (grayscale), VGLUT1 (magenta), and PSD95 (green). Yellow arrowheads indicate colocalization. (**E**) Bar charts of colocalized VGLUT1/PSD95 and GluN1/SV2A puncta normalized to neurite length. Overlap of pre- and postsynaptic puncta was increased in ZNF804A^+E3/−E3^ and ZNF804A^−E3/−E3^ neurons. VGLUT1/PSD95: Kruskal-Wallis: χ^2^(2) = 6.6, *P* = 0.04, *n* = 15; Dunn’s post hoc with Holm corrections confirmed ~2.1-fold increases in both mutant genotypes. GluN1/SV2A: Kruskal-Wallis χ^2^(2) = 7.2, *P* = 0.03 (*n* = 16); post hoc test showed 4.1- to 5.4-fold increases in mutants (*P*_adj_ < 0.05). (**F**) ZNF804A^+E3/−E3^ and ZNF804A^−E3/−E3^ neurons displayed more extremities [Kruskal-Wallis: χ^2^(2) = 11.4, *P* = 0.003, *n* = 16] and longer neurites [Kruskal-Wallis: χ^2^(2) = 11.3, *P* = 0.003, *n* = 16] than controls. Error bars: mean ± SD, *P_adj_ < 0.05. Created in BioRender. Powell, T. (2026) https://BioRender.com/vh5vr9x.

Our transcriptomic analysis and the extant literature (*21*, *27*) suggests disruptions of the glutamatergic synapse in ZNF804A mutation models. Therefore, we investigated the impact of ZNF804A exon 3 excision on developing glutamatergic synapses by examining the density of glutamatergic pre- and postsynaptic proteins and their colocalization across various cellular compartments in D7 glutamatergic neurons. We conducted this analysis using high-throughput confocal imaging and automated image analysis, detecting puncta specifically in cell soma (somata), neurites, and across the entire cellular regions of MAP2^+^ cells. Protein puncta were analyzed relative to neurite length, somata count, and overall cell quantity and normalized to corresponding values obtained from wild-type clones within the same experimental setup. We obtained data from 54,654 MAP2^+^ neurons (ZNF804A^+E3/+E3^_4: 4681, ZNF804A^+E3/+E3^_61: 11,445, ZNF804A^+E3/−E3^_10.23: 5323, ZNF804A^+E3/−E3^_42.13: 7323, ZNF804A^−E3/−E3^_20: 6833, and ZNF804A^−E3/−E3^_44: 19,049) for postsynaptic GluN1 staining and 45,371 neurons (ZNF804A^+E3/+E3^_4: 4241, ZNF804A^+E3/+E3^_61: 10,302, ZNF804A^+E3/−E3^_10.23: 5257, ZNF804A^+E3/−E3^_42.13: 5580, ZNF804A^−E3/−E3^_20: 5550, and ZNF804A^−E3/−E3^_44: 14,441) for presynaptic VGLUT1. While genotype had no statistically significant effect on GluN1 puncta count in somata [Kruskal-Wallis test: χ^2^(2) = 0.05, *P* = 0.9, *n* = 18] or entire cell regions [one-way ANOVA: *F*(2,15) = 0.3, *P* = 0.8, *n* = 18], the significance threshold was reached when analyzing MAP2^+^ neurites [Kruskal-Wallis test: χ^2^(2) = 8.49, *P* = 0.01, *n* = 18]. Dunn’s test with Holm corrections showed that this was driven by increased puncta count in neurites of ZNF804A^+E3/−E3^ mutation neurons compared to controls [*P*_adj_ < 0.05, mean FC from wt = 4.7 (range: 1.1 to 20.7)]. Although the post hoc comparisons between ZNF804A^+E3/+E3^ and ZNF804A^−E3/−E3^ mutation neurons just failed to reach the threshold for statistical significance (*P*_adj_ = 0.055), it is important to note that there was an increase in GluN1 puncta count in neurites of ZNF804A^−E3/−E3^ neurons in all but one replicate, with a mean FC of 4.8 (range: 1.2 to 18.2; Fig. 4, A and B). The same analysis for presynaptic VGLUT1 showed significant effects for genotype on puncta counts within somata [one-way ANOVA: *F*(2,12) = 4.5, *P* = 0.03, *n* = 15], the entire cell regions [Kruskal-Wallis test: χ^2^(2) = 8.5, *P* = 0.007, *n* = 15], and puncta counts along MAP2^+^ neurites [Kruskal-Wallis test: χ^2^(2) = 6.4, *P* = 0.04, *n* = 15]. While post hoc tests showed significant reductions of VGLUT1 puncta in somata [mean FC of ZNF804A^−E3/−E3^ from ZNF804A^+E3/+E3^ = 0.8 (range: 0.6 to 0.9), Tukey HSD: *P*_adj_ < 0.05] and entire cell regions with ZNF804A^−E3/−E3^ and ZNF804A^+E3/−E3^ mutations [Dunn’s test with Holm corrections: mean FC of ZNF804A^−E3/−E3^ from ZNF804A^+E3/+E3^ = 0.8 (range: 0.6 to 1), *P*_adj_ < 0.05; mean FC of ZNF804A^+E3/−E3^ from ZNF804A^+E3/+E3^ = 0.8 (range: 0.6 to 1), *P*_adj_ < 0.05], neurites of ZNF804A^−E3/−E3^ [Dunn’s test with Holm corrections: mean FC from ZNF804A^+E3/+E3^ = 3.3 (range: 1 to 8.3), *P*_adj_ < 0.05] and ZNF804A^+E3/−E3^ mutation neurons [mean FC from ZNF804A^+E3/+E3^ = 3.1 (range: 1.1 to 6.9), *P*_adj_ < 0.05] showed a significant increase in VGLUT1 puncta counts (Fig. 4, A and B). Analysis of further pre- and postsynaptic proteins (Bassoon, SV2A, and PSD95) showed similar effects for ZNF804A^−E3/−E3^ and ZNF804A^+E3/−E3^ mutations (fig. S8, A to F). Effect size analysis of main statistical tests revealed that the ZNF804A genotype had the largest effects on VGLUT1 and PSD95 puncta in the entire cell regions (η^2^ = 0.65) and substantial effects on pre- and postsynaptic protein puncta in neurites (η^2^ = 0.37 to 0.42). Genotype had small effects on puncta counts in somatic regions except for a large effect on VGLUT1 puncta in somatic regions (η^2^ = 0.42; Fig. 4C). This indicates a pronounced impact of ZNF804A mutation on glutamatergic synaptic proteins in different cellular compartments of developing glutamatergic neurons.

To complement our analysis of synaptic density, we next assessed neurite architecture by quantifying MAP2^+^ neurite length and endpoint tips as proxies for neuronal complexity and branching. We found that ZNF804A^+E3/−E3^ and ZNF804A^−E3/−E3^ neurons had significantly more extremities [Kruskal-Wallis test: χ^2^(2) = 11.4, *P* = 0.003, *n* = 16] and longer neurites [Kruskal-Wallis test: χ^2^(2) = 11.3, *P* = 0.003, *n* = 16] than ZNF804A^+E3/+E3^ control neurons (Fig. 4, D and F). This increase suggests enhanced structural complexity and arborization, consistent with elevated synaptic density. Next, we assessed colocalization of pre- and postsynaptic puncta along MAP2^+^ neurites of 45,371 neurons to quantify putative immature synapses (Fig. 4, D and E). Overlaid puncta count was normalized to neurite length to account for the enhanced structural complexity in mutant neurons (Fig. 4, D and F). A statistical analysis on the averaged technical replicates showed significant group differences in colocalization of both VGLUT1 and PSD95 [Kruskal-Wallis test: χ^2^(2) = 6.6, *P* = 0.04, *n* = 15] as well as GluN1 and SV2A [Kruskal-Wallis test: χ^2^(2) = 7.2, *P* = 0.03, *n* = 16] quantities with genotype as an independent variable. Dunn’s post hoc analysis with Holm corrections confirmed significant increases in colocalized VGLUT1 and PSD95 puncta along neurites of ZNF804A^+E3/−E3^ [mean FC from ZNF804A^+E3/+E3^ = 2.1 (range: 1.26 to 3.54), *P*_adj_ < 0.05] and ZNF804A^−E3/−E3^ [mean FC from ZNF804A^+E3/+E3^ = 2.1 (range: 1.01 to 3.6), *P*_adj_ < 0.05] mutation neurons. Dunn’s post hoc analysis with Holm corrections also confirmed significant increases in colocalized GluN1 and SV2A puncta along neurites of ZNF804A^+E3/−E3^ [mean FC from ZNF804A^+E3/+E3^ wt = 5.4 (range: 0.9 to 24.9), *P*_adj_ < 0.05] and ZNF804A^−E3/−E3^ [mean FC from ZNF804A^+E3/+E3^ = 4.13 (range: 1.1 to 14.3), *P*_adj_ < 0.05] mutation neurons (Fig. 4, D and E). Overall, these data suggest that components of the glutamatergic synapse are overrecruited to neurites in ZNF804A mutant neurons, potentially affecting the formation of developing synapses.

### ZNF804A mutant neurons show enhanced depolarization-evoked calcium responses

To determine whether the redistribution and increased abundance of synaptic proteins in ZNF804A mutant neurites are accompanied by functional changes, we assessed depolarization-evoked calcium responses as a readout of neuronal functional competence. At this early stage of differentiation (D7), *NGN2*-induced neurons do not exhibit spontaneous activity; therefore, depolarization-evoked calcium signaling provides a controlled measure of neuronal responsiveness rather than direct synaptic transmission per se.

**Fig. 5.**
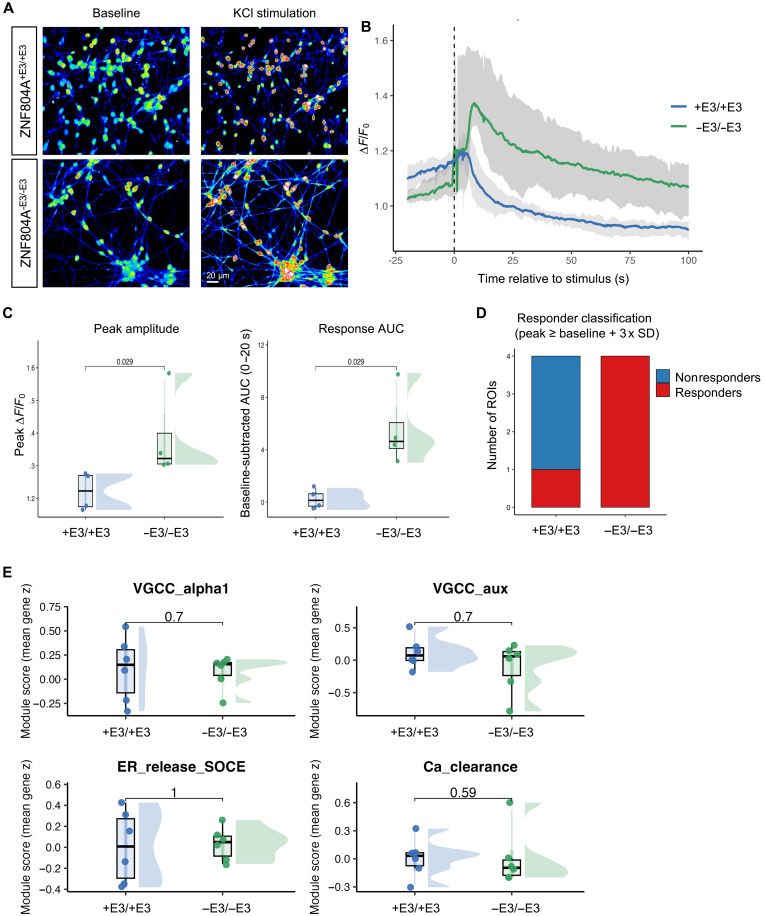
Enhanced and prolonged depolarization-evoked calcium responses in ZNF804A mutant neurons, independent of calcium channel gene expression. (**A**) Representative images of Flou-4-AM–loaded day 7 ZNF804A^+E3/+E3^ and ZNF804A^−E3/−E3^ neurons at baseline and following KCl (30 mM) stimulation. (**B**) Mean baseline-normalized calcium responses (Δ*F*/*F*_0_) aligned to stimulus onset (*t* = 0 s; dashed line) in ZNF804A^+E3/+E3^ and ZNF804A^−E3/−E3^ neurons. Solid lines represent the mean across all regions of interest (ROIs), and shaded regions indicate the full ROI-level range (minimum to maximum) at each time point. (**C** and **D**) Quantification of calcium response features at the ROI level, including (C) peak amplitude, baseline-subtracted response area under the curve (AUC; 0 to 20 s poststimulus), peak latency, rise time (10 to 90% of peak), and (D) responder classification. ROIs were classified as responders if peak Δ*F*/*F*_0_ exceeded baseline mean + 3× baseline SD. Raincloud plots show the distribution of individual ROIs (half-violin densities), with overlaid boxplots indicating median and interquartile range and points representing individual ROIs. ZNF804A^+E3/+E3^ and ZNF804A^−E3/−E3^ conditions were compared using two-sided Wilcoxon rank-sum tests; exact *P* values are shown. (**E**) Module-level expression of genes associated with calcium handling machinery (VGCC_alpha1, VGCC_aux, ER_release_SOCE, and Ca_clearance) derived from RNA sequencing. Module scores represent the mean *z*-scored expression across genes within each module for individual samples. ZNF804A^+E3/+E3^ versus ZNF804A^−E3/−E3^ comparisons were performed using two-sided Wilcoxon rank-sum tests.

Following KCl-induced depolarization, ZNF804A^−E3/−E3^ neurons exhibited enhanced calcium responses compared with ZNF804A^+E3/+E3^ controls (Fig. 5, A and C). Peak Δ*F*/*F*_0_ was significantly increased in ZNF804A^−E3/−E3^ neurons (ZNF804A^+E3/+E3^: 1.222 ± 0.029; ZNF804A^−E3/−E3^: 1.383 ± 0.067; Wilcoxon rank-sum test, *P* = 0.029; Fig. 5C), indicating a higher maximal calcium response. Similarly, baseline-subtracted response area under the curve (AUC), was significantly increased in ZNF804A^−E3/−E3^ neurons (ZNF804A^+E3/+E3^: 0.192 ± 0.373; ZNF804A^−E3/−E3^: 5.534 ± 1.490; Wilcoxon rank-sum test, *P* = 0.029; Fig. 5C), reflecting a more sustained elevation of intracellular calcium following depolarization. Consistent with this, ZNF804A^−E3/−E3^ neurons displayed a greater fraction of responding regions of interest (ROIs), suggesting increased response reliability across cells (Fig. 5D).

To assess whether these functional differences were due to altered expression of calcium channel genes, we interrogated the RNA-seq dataset for transcriptional changes in pathways associated with calcium entry, release, and clearance. Module-level analysis (table S5) revealed that gene programs encompassing voltage-gated calcium channel subunits, intracellular calcium release pathways, and calcium clearance mechanisms were largely preserved between ZNF804A^+E3/+E3^ and ZNF804A^−E3/−E3^ neurons (Fig. 5, E and F). In contrast, modules associated with synaptic readiness, excitability, and developmental receptor switching exhibited more pronounced genotype-dependent shifts (fig. S9, A to C).

Together, these findings demonstrate that enhanced and prolonged depolarization-evoked calcium responses in D7 ZNF804A mutant neurons occur in the absence of detectable transcriptional remodeling of calcium handling machinery. Instead, they are consistent with altered synaptic organization and excitability, providing functional support for the synaptic protein redistribution observed in D7 mutant neurons.

### Isolating neurite- and soma-specific proteomes in developing glutamatergic neurons

**Fig. 6.**
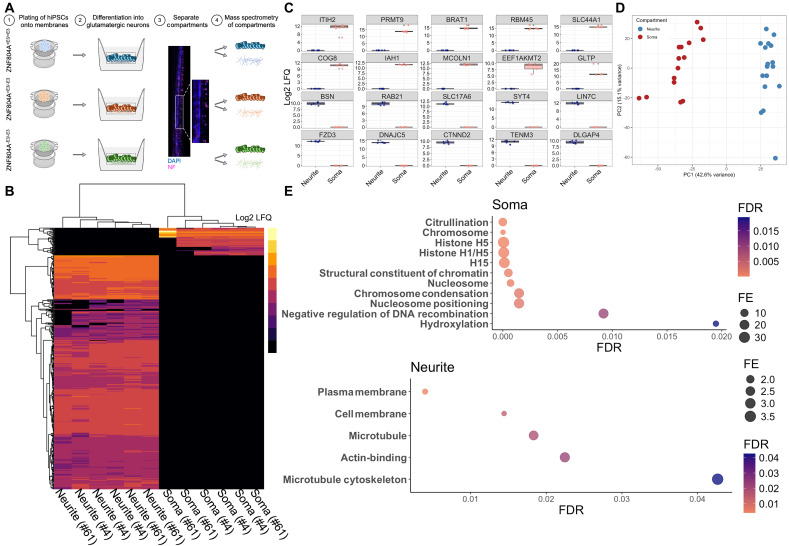
Separation of neurite and soma proteomes. (**A**) Schematic representation of cellular compartment separation assay. Created in BioRender. Powell, T. (2026) https://BioRender.com/vh5vr9x. (**B**) Unsupervised heatmap of log2-transformed label-free quantification (LFQ) of proteins solely detected in either the neurite or soma compartment of control ZNF804A^+E3/+E3^ neurons as measured by LC-MS/MS in data-independent acquisition (DIA) mode. Unsupervised heatmap analysis showed clear hierarchical clustering of samples according to cellular compartment. (**C**) Boxplots of the most highly expressed proteins in the neurite compartments including bassoon presynaptic cytomatrix protein (BSN), SAP90/PSD-95-associated protein 4 (DLGAP4), solute carrier family 17 (vesicular glutamate transporter), and member 6 (SLC17A6) and synaptotagmin 4 (SYT4). Boxplots of proteins that were highly expressed only in the somata compartment including nuclear proteins such as BRCA1-associated ATM activator 1 (BRAT1) and protein arginine methyltransferase 9 (PRMT9), as well as proteins associated with the Golgi apparatus [component of oligomeric Golgi complex 8 (COG8)] and mitochondria [solute carrier family 44 member 1 (SLC44A1)]. (**D**) Principal components analysis (PCA) of 32 samples colored by the neurite (blue) or soma (red) compartment. (**E**) GO analysis of somata-enriched (top) and neurite-enriched (bottom) proteins. The *x* axis displays FDR, and the *y* axis lists terms with significant enrichment (FDR < 0.05). Icon size represents fold enrichment.

High-throughput confocal imaging analysis suggested an increased localization of synaptic proteins to MAP2^+^ neurites as well as increased colocalization of pre- and postsynaptic proteins in ZNF804A mutant developing glutamatergic neurons. However, the expression of synaptic proteins in the somata and entire cell regions were either decreased or remained unchanged (Fig. 4, A to E). Furthermore, bulk RNA-seq did not reveal any genotype-related transcriptome-wide variations in these synaptic genes (table S3). Collectively, these data suggest that the increase in synaptic proteins within neurites likely occurs through a mechanism independent of gene expression changes. On the basis of the evidence that ZNF804A binds to ribosomal proteins and regulates protein translational efficiency (*25*), we hypothesized that neurite-localized change in synaptic proteins may be due to changes in local protein synthesis. Hence, we tested whether ZNF804A exon 3 excision leads to an alteration in the local proteome of distinct subcellular compartments. To this end, we separated neurite and cell soma compartments of developing glutamatergic neurons and used spatial proteomic analyses to examine the local proteome of each subcellular compartment (*37*). Cell lines from all three genotypes were differentiated on a membrane with a laminin-coated underside allowing neurites to extend through pores, enabling the separation of cell bodies from neurite processes (Fig. 6A). We validated the effective separation of neurites from cell soma using Western blotting and liquid chromatography–coupled tandem mass spectrometry (LC–MS/MS) (Fig. 6 and fig. S10A). We lysed neurites and somata grown on the same membrane separate from each other for proteomic analysis. We used LC–MS/MS in data-independent acquisition (DIA) mode on a total of 35 samples (neurites: ZNF804A^+E3/+E3^ *n* = 6, ZNF804A^+E3/−E3^ *n* = 5, ZNF804A^−E3/−E3^ *n* = 6; somata: ZNF804A^+E3/+E3^ *n* = 6, ZNF804A^+E3/−E3^ *n* = 6, ZNF804A^−E3/−E3^ *n* = 6) to analyze the localized proteomes of each subcompartment. Using a label-free quantification (LFQ) approach, we successfully detected a total of 5393 proteins (range per sample: 581 to 5307; table S6). To confirm the successful separation of neurites from somata (cell soma), we first assessed expression of proteins solely detected in either compartment of ZNF804A^+E3/+E3^ control neurons. A total of 404 proteins were only detected in the neurite compartment, whereas 48 proteins were only detected in the somata compartment. Unsupervised heatmap analysis of these proteins showed clear hierarchical clustering of samples according to cellular compartment (Fig. 6B). The most highly expressed proteins found only in the neurite compartment included the pre- and postsynaptic proteins: bassoon presynaptic cytomatrix protein (BSN), SAP90/PSD-95-associated protein 4 (DLGAP4), solute carrier family 17 (vesicular glutamate transporter), and member 6 (SLC17A6) and synaptotagmin 4 (SYT4). Proteins that were highly expressed only in the somata compartment included nuclear proteins such as BRCA1-associated ATM activator 1 (BRAT1) and protein arginine methyltransferase 9 (PRMT9), as well as proteins associated with the Golgi apparatus [component of oligomeric Golgi complex 8 (COG8)] and mitochondria [solute carrier family 44 member 1 (SLC44A1)] (Fig. 6C). These data confirmed the successful separation of neurites from cell soma.

To refine our analysis, we removed proteins expressed in fewer than 70% of the samples and three samples showing significantly lower identified proteins (ZNF804A^+E3/−E3^_10.23_RP3_soma, ZNF804A^+E3/−E3^_61_RP3_soma, and ZNF804A^+E3/−E3^_42.23_RP3_soma). We performed PCA on the remaining 32 samples and showed clear clustering according to cellular compartment. Protein expression patterns across biological replicates within each fraction (*n* = 3 per genotype; two clones per genotype) were highly consistent, suggesting minimal cross-contamination or variability in the separation process (Fig. 6D). To further confirm the effectiveness of our approach to separate neurites from somata, we then compared neurite- and soma-specific proteomes (regardless of genotype) by applying the limma package for differential protein expression analysis (*38*). This allowed us to assess proteome-wide differences between subcellular samples. In total, 351 proteins were differentially expressed (*P*_adj_ < 0.05, log2FC > 1.5 or <−1.5), comprising 224 proteins showing greater enrichment in neurites and 127 proteins displaying a higher abundance in somata (fig. S10B and table S7). As expected, GO analysis of differentially expressed proteins confirmed that neurite-enriched proteins are significantly enriched in gene lists termed “Cell Membrane” (FE = 1.5, *P* < 0.01, FDR q = 0.01), “Actin-binding” (FE = 2.8, *P* < 0.001, FDR q = 0.02), and “Microtubule Cytoskeleton” (FE = 3.5, *P* < 0.001, FDR q = 0.04), whereas somata-enriched proteins overlapped with genes listed under “Nucleosome” (FE = 7.7, *P* < 0.001, FDR q < 0.001), “Structural Constituent of Chromatin” (FE = 12.2, *P* < 0.001, FDR q < 0.001), and “Chromosome Condensation” (FE = 21.1, *P* < 0.001, FDR q = 0.001) (Fig. 6E and table S8). Thus, these data validate the successful separation of neurites and somata.

### Subcellular compartment–specific proteome analysis suggests overrecruitment of translational machinery to ZNF804A mutant neurites

Having validated the cellular compartment separation model, we next explored our hypothesis that the neurite-localized change in synaptic proteins in ZNF804A mutant lines may be due to changes in local protein synthesis. We first confirmed the neurite-localized change in synaptic proteins by extracting log2-transformed LFQ values. Two-sample Student’s *t* test confirmed up-regulation of pre- and postsynaptic proteins in ZNF804A^+E3/−E3^ [e.g., ARGLU1: *t*(7) = −3.68, *P* < 0.05; STX1A: *t*(4) = −9.39, *P* < 0.001; STXBP1: *t*(5) = −5.17, *P* < 0.01; SNCG: *t*(6) = −3.02, *P* < 0.05] and ZNF804A^−E3/−E3^ [e.g., ARGLU1: *t*(9) = −3.75, *P* < 0.01; SNAP29: *t*(8) = −3.75, *P* < 0.01; SYN3: *t*(5) = −4.39, *P* < 0.01; VTI1B: *t*(6) = −2.73, *P* < 0.05] cells compared to ZNF804A^+E3/+E3^ controls (fig. S11).

**Fig. 7.**
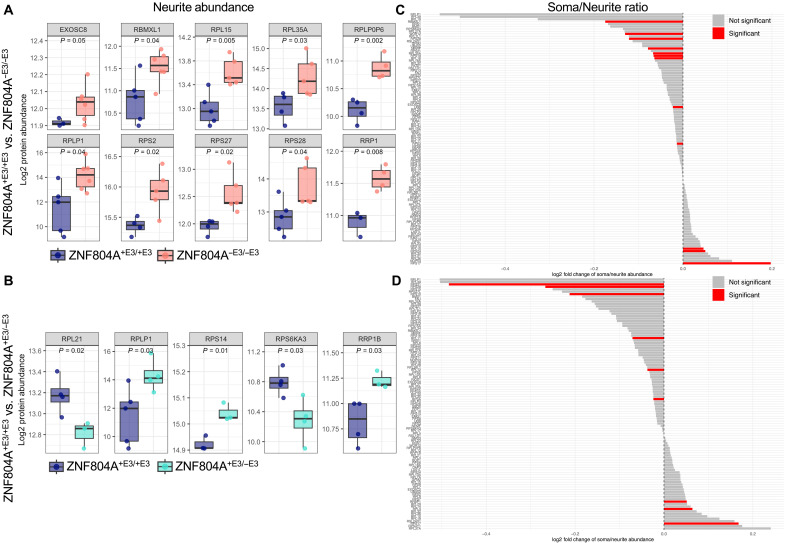
Overrecruitment of translational machinery to ZNF804A mutant neurites. (**A**) Boxplots of log2-transformed LFQ values showing significant increase of proteins associated with ribosomal function in neurite compartment samples of ZNF804A^−E3/−E3^ neurons compared to controls. (**B**) Boxplots of log2-transformed LFQ values showing significant increase of proteins associated with ribosomal function in neurite compartment samples of ZNF804A^+E3/−E3^ neurons compared to controls. (**C** and **D**) Bar plots representing the abundance ratios (*x* axis) for each tested protein (*y* axis) in ZNF804A^−E3/−E3^ (C) or ZNF804A^−E3/+E3^ (D) samples by dividing the log2-transformed LFQ values in the somata by those in the corresponding neurites within the same replicate. Positive values indicate higher abundance in somata, whereas negative values indicate higher abundance in neurite compartments.

Next, we tested whether ribosomes mislocalize in ZNF804A exon 3 excision conditions. To that end, we extracted log2-transformed LFQ values from 99 proteins associated with ribosomal function in neurite compartment samples and performed statistical analysis to compare mean differences of ribosomes localized in ZNF804A^+E3/−E3^ and ZNF804A^−E3/−E3^ neurites compared to controls. In ZNF804A^−E3/−E3^ neurites, 14% of tested proteins showed significant differences compared to control lines (Fig. 7A), whereas 10% of tested proteins significantly differed in ZNF804A^+E3/−E3^ neurites (Fig. 7B). Large ribosomal subunit 60S (RPLP1), small ribosomal subunit 40S (RPS21 and RPS27), and RNA-binding proteins (RBMXL1) were significantly up-regulated in neurites of mutant cells. Next, we evaluated whether proteins may be overrecruited to distal cellular regions in ZNF804A^−E3/−E3^ and ZNF804A^+E3/−E3^ mutant conditions by calculating the abundance ratios for each protein by dividing the log2-transformed LFQ values in the somata by those in the corresponding neurites within the same replicate. Twelve percent of ribosomal proteins showed significant mislocalization in ZNF804A^−E3/−E3^ (Fig. 7C) and 9% in ZNF804A^+E3/−E3^ (Fig. 7D) conditions, most of which showed significant overrecruitment from the soma to distal regions of the cell. These included both large ribosomal subunit 60S (RPLP1) and small ribosomal subunit 40S (RPS21 and RPS27) proteins. We observed substantial fold changes in ribosomal protein levels under ZNF804A^−E3/−E3^ and ZNF804A^+E3/−E3^ conditions. However, these changes did not reach statistical significance, primarily due to their detection in only a limited number of samples, which excluded them from statistical testing.

### ZNF804A exon 3 excision yields increased protein synthesis efficiency within neurites

**Fig. 8.**
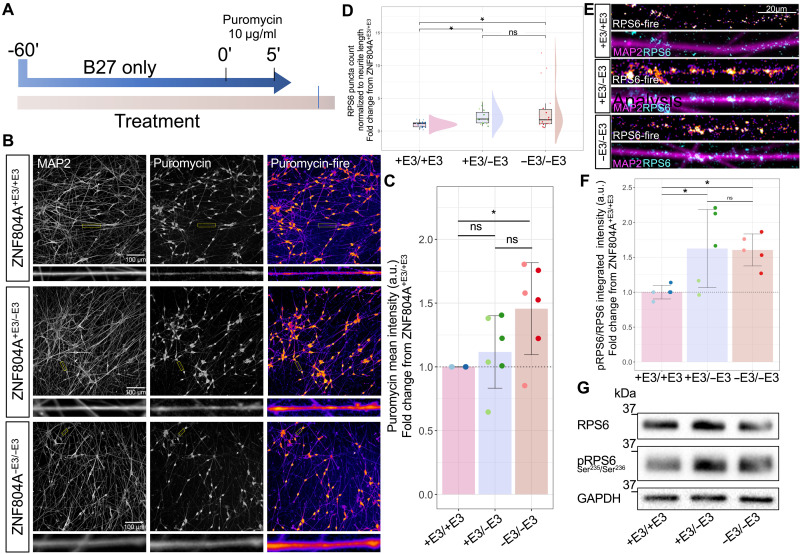
Local protein synthesis efficiency is increased in ZNF804A-deficient neurites. (**A**) Experimental timeline of SUnSET assay. (**B**) Representative confocal image of puromycin incorporation (fire LUT) in MAP2^+^ neurites of ZNF804A^+E3/+E3^, ZNF804A^+E3/−E3^, and ZNF804A^−E3/−E3^ D7 neurons captured with a high-content microscope. (**C**) Bar chart showing quantification of mean intensities of puromycin staining in MAP2^+^ neurites. Overlaying dot plots show staining intensities for each clone. ZNF804A^−E3/−E3^ show significantly increased puromycylated polypeptides in MAP2^+^ neurites [one-way ANOVA: *F*(2,15) = 4.8, *P* = 0.02, *n* = 18]. (**D**) Raincloud plot showing quantification of RPS6 puncta in MAP2^+^ neurites. Overlaying dot plots show puncta counts for each technical replicate. RPS6 puncta were significantly increased in MAP2^+^ neurites of ZNF804A^+E3/−E3^ and ZNF804A^−E3/−E3^ neurons [Kruskal-Wallis test: χ^2^(2) = 7.7, *P* = 0.021, *n* = 18]. (**E**) Representative confocal image of ribosomal protein S6 (RPS6; cyan) puncta in MAP2^+^ neurites (magenta) of ZNF804A^+E3/+E3^, ZNF804A^+E3/−E3^, and ZNF804A^−E3/−E3^ D7 neurons. (**F**) Representative Western blot of full and phosphorylated RPS6 (~32 kDa) and housekeeper GAPDH. (**G**) Bar charts showing quantification of integrated densities (ID) of RPS6 total, and phosphorylated (Ser^235^/Ser^236^) protein bands. Overlaying dot plots show IDs for each clone. Phosphorylated RPS6 was increased in ZNF804A^+E3/−E3^ and ZNF804A^−E3/−E3^ D7 neurons [one-way ANOVA: *F*(2,12) = 5.06, *P* = 0.02, *n* = 15]. Error bars: mean ± SD, *P_adj_ < 0.05.

As our proteomic data suggested an overrecruitment of translational machinery and regulatory effectors of protein synthesis to neurites in ZNF804A^+E3/−E3^- and ZNF804A^−E3/−E3^-mutant neurons, we next assessed whether this had a functional impact on protein translation. To this end, we used the surface sensing of translation (SUnSET) assay, which we previously validated [fig. S12; (*39*)]. Here, we used SUnSET in combination with ICC and high-throughput imaging to assess localized protein synthesis efficiency in MAP2^+^ neurites. Cell lines of each genotype underwent three distinct differentiations, and before fixation, cells were treated with puromycin (10 μg/ml) for 5 min. Puromycin incorporation into the newly forming polypeptide chain was visualized using immunolabeling and high-content imaging confocal microscopy (Fig. 8A). An automated analysis script measured the mean intensity of puromycin staining in neurites of MAP2^+^ cells. A total of 35,461 MAP2^+^ cells (ZNF804A^+E3/+E3^_4: 8539, ZNF804A^+E3/+E3^_61: 5313, ZNF804A^+E3/−E3^_10.23: 7268, ZNF804A^+E3/−E3^_42.13: 5487, ZNF804A^−E3/−E3^_20: 4970, and ZNF804A^−E3/−E3^_44: 3353) were identified in this experiment. As above, we performed statistical analysis on the averaged mean signal intensities of each clone per replicate to avoid pseudo-replication, and we normalized puromycin signal intensities within neurites of each clone to the ZNF804A^+E3/+E3^ clone imaged in the same plate to calculate an FC difference. One-way ANOVA revealed significant differences in puromycin mean intensities within neurites due to genotype [*F*(2,15) = 4.8, *P* = 0.02]. This statistically significant difference was attributed to an increase in puromycin staining intensities in neurites of ZNF804A^−E3/−E3^ mutation lines [Tukey HSD: mean FC from wt = 1.5 (range: 0.9 to 1.8), *P*_adj_ < 0.05]. Puromycin staining within neurites from ZNF804A^+E3/−E3^ mutation lines was not significantly different from ZNF804A^+E3/+E3^ counterparts [Tukey HSD: mean FC from wt = 1.1 (range: 0.6 to 1.8), *P*_adj_ > 0.05; Fig. 8, B and C]. This implies that ZNF804A^−E3/−E3^ mutation results in a significant increase of newly synthesized proteins in neurites of developing human glutamatergic neurons, supporting the more exploratory proteomic findings. To determine whether this increase is accompanied by elevated levels of key components of the translational machinery in distal regions beyond the soma, we visualized ribosomal protein S6 (RPS6) staining in neurites using the high-content imaging approach described above. We imaged RPS6 puncta in three differentiations involving two clones per genotype (*n* = 17, 6 ZNF804A^+E3/+E3^, 5 ZNF804A^+E3/−E3^, and 6 ZNF804A^−E3/−E3^—we excluded one replicate of ZNF804A^+E3/−E3^_42.13 due to staining failure). An automated analysis script identified RPS6 puncta in neurites of 55,150 MAP2^+^ cells (ZNF804A^+E3/+E3^_4: 6712, ZNF804A^+E3/+E3^_61: 13,241, ZNF804A^+E3/−E3^_10.23: 8984, ZNF804A^+E3/−E3^_42.13: 4838, ZNF804A^−E3/−E3^_20: 7772, and ZNF804A^−E3/−E3^_44: 13,603). We normalized RPS6 puncta counts as above and observed a significant group difference when comparing counts across genotype [Kruskal-Wallis test: χ^2^(2) = 7.7, *P* = 0.021; Fig. 8, D and E]. Post hoc Dunn’s test with Holm adjustments revealed significant increases in RPS6 puncta in ZNF804A^−E3/−E3^ [mean FC from wt = 3.1 (range: 0.7 to 10.1), *P*_adj_ < 0.05] and ZNF804A^+E3/−E3^ [mean FC from wt = 2.2 (range: 1.2 to 3.2), *P*_adj_ < 0.05] conditions compared to ZNF804A^+E3/+E3^ (Fig. 8E), supporting proteomic findings indicating an overrecruitment of translational machinery to neurites. Last, to identify a potential mechanism driving these changes in local protein synthesis, we tested abundance of RPS6 phosphorylation at Ser^235^/Ser^236^ using Western blotting. We adopted this approach because RPS6 phosphorylation is known to predominantly affect the translation of a specific subset of mitochondrial-related mRNAs, not having a global impact on translation (*40*, *41*) and it is phosphorylated at Ser^235^/Ser^236^ near active synapses in response to synaptic activity (*42*), suggesting its potential significance in regulating the local proteome rather than the global one. While group comparisons of full protein abundances were not statistically significant [Kruskal-Wallis test: χ^2^(2) = 5.6, *P* = 0.06, *n* = 15], phosphorylated RPS6 exhibited a significant increase in both ZNF804A^−E3/−E3^ and ZNF804A^+E3/−E3^ lines [one-way ANOVA: *F*(2,12) = 5.06, *P* = 0.02, *n* = 15; Tukey HSD: ZNF804A^+E3/+E3^ versus ZNF804A^−E3/−E3^ and ZNF804A^+E3/+E3^ versus ZNF804A^+E3/−E3^: *P*_adj_ < 0.05, ZNF804A^+E3/−E3^ versus ZNF804A^−E3/−E3^ *P*_adj_ > 0.05; mean FC from wt: ZNF804A^−E3/−E3^ = 1.6 (range: 1.3 to 1.9), ZNF804A^+E3/−E3^ = 1.62 (range: 0.9 to 2.2); Fig. 8, F and G]. Overall, these results are in support of the proteomic data suggesting that ZNF804A exon 3 excision results in an overrecruitment of translational machinery to neurites and offer a previously unexplored perspective for the function of the SZ susceptibility gene in local translational control.

## DISCUSSION

There remains a considerable gap in our understanding of observable neurobiological processes linked to the genetic susceptibility of complex psychiatric conditions such as SZ. In this study, we characterized the function of a robust SZ risk gene, ZNF804A, in a cell type and at a neurodevelopmental time point relevant for peak human brain ZNF804A gene expression and SZ etiology. We first established developing human glutamatergic neurons, starting to take on an upper layer cortical neuronal fate, as a suitable model to study ZNF804A gene function. We then applied a dual-sgRNA based CRISPR-Cas9 design to excise exon 3 of the ZNF804A locus. We successfully validated two clones per excision genotype (ZNF804A^+E3/+E3^, ZNF804A^+E3/−E3^, and ZNF804A^−E3/−E3^) and confirmed transcriptomic disruptions due to ZNF804A mutation in genes associated with cell adhesion and glutamatergic synapses using bulk RNA-seq. Dysregulated genes in ZNF804A^−E3/−E3^ mutant neurons were enriched for a gene set that has been found to be dysregulated in postmortem frontal cortex tissue obtained from patients with SZ, which strengthens the role of ZNF804A in the disorder and supports the validity of our model. We then proceeded to test hypotheses generated by our RNA-seq results and the extant literature on ZNF804A function encompassing a variety of human cellular models and gene targeting approaches. We assessed the expression and density of glutamatergic synaptic proteins and early putative synapses in mutation lines in a high-throughput confocal imaging experimental setup. Unexpectedly, both pre- and postsynaptic proteins as well as putative synapses were increased in ZNF804A mutant MAP2^+^ neurites (Fig. 4, A to F). However, mass spectrometry analysis of neurite and soma proteomes suggested no proteome-wide differences in protein expression according to genotype, but rather significant differences in neurite/somata protein abundance ratios of translational machinery and translation regulation proteins (Fig. 7, A to D). This suggests an overrecruitment of translational machinery to ZNF804A mutant MAP2^+^ neurites, which was accompanied by increases in local protein synthesis efficiency (Fig. 8, B and C). Moreover, we observe that KCl-driven stimulation driven calcium signaling is enhanced in ZNF804A mutant neurons (Fig. 5, A to D), consistent with an altered synaptic organization and increased excitability. To our knowledge, the mechanisms by which ZNF804A functions at this stage of early neuronal development and in this cell type have not been analyzed before and results of this study will aid in our understanding of how disruptions in this gene contribute to SZ etiology during neurodevelopment.

A major consideration of our study design was to develop a cellular model that is relevant to both susceptibility gene function and the neurobiology of SZ. As it has been proven difficult to translate the plethora of identified common disease-associated genetic variants into observable neurobiological processes (*4*), we aimed to take information about disorder-relevant cell types and developmental time points into account, laying the foundation of a previously unexplored functional genetics approach. Previous research has identified the second trimester of fetal development as a developmental period when SZ risk genes reach a peak in expression (*43*) and single-cell transcriptomics demonstrated that common risk genes were specifically expressed in immature glutamatergic neurons of the fetal frontal cortex and hippocampus (*5*, *44*). In line with this, existing literature (*28*, *29*) and our findings showed a peak of *ZNF804A* expression, both full-length and SZ risk variant *ZNF804A^E3E4^*, in developing cells, particularly in glutamatergic neurons with a forebrain, cortical upper-layer identity. Notably, our statistical analysis yielded the largest effect size for developmental time point on transcript expression, underlining the importance of taking temporal context specificity into account when characterizing gene function. Furthermore, the CRISPR-Cas9–mediated excision of exon 3 allowed for the generation of isogenic mutation of both full-length and *ZNF804A^E3E4^*, which has been specifically linked to SZ diagnosis and risk allele expression (*29*). We then confirmed the validity of the model by holistic transcriptomic analysis, showing transcriptomic disruptions in ZNF804A mutation lines linked to the glutamatergic synapse and cell adhesion, which is a replication of a robust finding in previous transcriptomic studies of ZNF804A function (*26*, *27*). Furthermore, down-regulated DEGs from signature A in our model were significantly enriched in an up-regulated gene set from postmortem brain tissue from individuals with SZ (FDR < 0.05) (*35*). Intriguingly, the observed effect seemed to manifest in opposite directions, possibly due to development-related differences in gene expression (development versus postmortem). Despite these differences in postmortem brain samples and our developmental model, a significant correlation between gene expression in idiopathic SZ and ZNF804A mutation suggests shared molecular processes, reinforcing the role of ZNF804A in the disorder. These results will provide a foundation from which studies assessing the function of ZNF804A in cell lines with different gene risk backgrounds (*45*) or studies where the combinatorial effects of perturbing the effects of multiple risk factors, including ZNF804A (*46*, *47*), can be conducted with knowledge of the mechanism by which this risk gene affects cellular and molecular function in developing neurons.

A key finding from our work is that ZNF804A potentially increases early glutamatergic synaptogenesis in developing neurons. Previous work in primary rodent neuronal cells showed that both knockdown and overexpression of Zfp804A (rodent homolog) resulted in decreased dendritic spine density (*21*, *22*). Furthermore, rodent neurons with reduced Zfp804A expression showed attenuated responses to activity-dependent spine plasticity (*21*). These findings suggest that a critical balance in ZNF804A expression is required for the maintenance and activity remodeling of dendritic spines in mature neurons. Consistent with these findings, our transcriptomic analysis of mutant ZNF804A neurons also showed significant disruptions in genes associated with the glutamatergic synapse. However, high-content confocal imaging of synaptic proteins as well as proteomic analysis showed an increased redistribution of pre- and postsynaptic proteins to MAP2^+^ neurites with ZNF804A mutations and an increase of VGLUT1 and PSD95 overlap, indicating a larger quantity of synapses (Figs. 4 and 6). The differences between the observations in the current study and those previously reported are likely due to different developmental time points analyzed. While decreases in dendritic spines were observed in more mature neurons with fully developed dendritic arbors, in our model, we observe increases in early synaptogenesis. This may indicate excessive formation of synapses due to ZNF804A mutation before neural circuitry is fully established—future studies will need to determine this at a functional level. A prominent theory is that excessive or aberrant synaptic pruning contributes to synaptic dysfunction in SZ (*48*). Studies using mutant mouse models with blocked neurotransmitter release have demonstrated that excitatory synapses can still form and function without glutamatergic neurotransmission (*49*–*51*), suggesting that developmental synapse formation is activity independent and potentially “hard wired” compared to postnatal and adult synaptogenesis. Thus, because of the difference in response to *ZNF804A* deficiency during the later stages of activity-dependent synaptogenesis compared to the initial phases of activity-independent synapse formation, ZNF804A might take on different roles at the synapse throughout neurodevelopment. The increases in synapses observed here early in development could potentially have profound implications, affecting crucial processes in later life, such as synapse specification or synaptic pruning (*52*, *53*), ultimately contributing to disordered signal transmission as observed in SZ.

A question that remains to be answered now is what caused this increase in putative early synapse formation in ZNF804A mutant neurons. Previously, ZNF804A has been shown to interact with ribosomal proteins (*25*) and to play a role in structural remodeling of dendritic spines in response to activity-dependent stimulation (*21*), suggesting a role for the susceptibility gene in local translational control. Dendritic translation was robustly implicated in long-term synaptic plasticity in mature neurons (*54*, *55*) and presynaptic axonal translation occurs during synapse formation (*56*). In addition, while RNA granules have been shown to dynamically move along main axons and dendrites, there is some evidence that stationary RNA granules not only localize to presynaptic terminals (*57*) but also translocate toward synaptic spine heads upon neuronal stimulation (*58*). This indicates that facilitation of local translation-dependent synapse formation is based on the function of stationary ribosomes near synapses. An interesting observation in our data is that ribosomes as well as proteins associated with translation regulation seem to be excessively recruited to MAP2^+^ dendrites of ZNF804A mutation neurons, which was accompanied by increased dendritic translation efficiency and RPS6^Ser235/236^ phosphorylation, a phosphorylation site specifically associated with local translation initiation (*42*). ZNF804A dysfunction may therefore contribute to excessive redistribution of stationary ribosomes within immature MAP2^+^ dendrites, leading to increased synapse formation early on in development. This is in contrast to findings from nonneuronal and proliferative cell systems, where ZNF804A has been proposed to act as a global translation promoter (*25*), underscoring a context- and cell type–dependent role for ZNF804A in translational control.

While the aim of this study was to characterize the neurobiological function of a robust SZ risk gene, it must be acknowledged that the deletion introduced into the gene locus here is not the SNP associated with the disorder. Certainly, this limits the ability to directly infer neurobiological dysfunction from genetic variation directly linked to SZ. To tackle this, future investigations may benefit from CRISPR-Cas9–mediated ZNF804A SNP modeling to establish a direct relation between genetic predisposition to neurobiology of SZ. It should also be noted that while multiple converging lines of evidence argue against the existence of a stable truncated ZNF804A proteoform following exon 3 excision; we cannot formally exclude the presence of low-abundance truncated ZNF804A protein encoded by the exon 2 to exon 4 fusion transcript. In addition, as our analyses focus on an early developmental time point, it remains possible that ZNF804A dysfunction exerts distinct or even opposing effects on synaptic organization and translational control at later stages of neuronal maturation. Furthermore, while ZNF804A expression is most pronounced in developing glutamatergic cells, transcripts of the susceptibility gene have also been found in other brain cell types including immature inhibitory neurons and radial glia (Fig. 1A). This suggests that its function is not exclusive to glutamatergic neurons. Therefore, the isogenic *ZNF804A* mutant model could be integrated with differentiation protocols aimed at generating cells of nonglutamatergic lineages, such as microglial (*59*, *60*) or GABAergic (*61*) cells, enabling the characterization of gene function in mono- or coculture paradigms. Last, translatability of findings is inevitably dependent on the characteristics of the study population. It becomes more and more evident that sex differences are often overlooked but important in not only the genetics of psychiatric disorders but also their underlying neurobiology (*62*). In the case of ZNF804A, there is evidence of a sex-specific SNP associated with SZ (*63*) as well as a mouse model indicating sex-dependent variations in SZ-like phenotypes (*64*). Hence, future investigations should take biological sex into consideration when modeling gene function.

Overall, this study characterizes the function of SZ susceptibility gene *ZNF804A* in a cellular model that incorporates developmental time point and cell type specificity. Furthermore, by demonstrating an excessive localization of ribosomes to dendrites of developing glutamatergic neurons coupled with an increase in early putative synapses, we suggest a link of the previously identified processes of ZNF804A function, synapse maintenance and translational control, by its ability to mediate local protein synthesis efficiency. Ultimately, research building on our findings will contribute to unravelling cellular neurodevelopmental processes directly linked to the etiology of SZ and potentially aid in the identification of targets for future therapeutic interventions.

## MATERIALS AND METHODS

### Experimental design

The overarching aim of this study was to characterize the function of a robust SZ susceptibility gene, *ZNF804A*, in a cellular model system that is both (i) relevant to gene function and (ii) SZ etiology. Therefore, to identify the most suited developmental time point and cell type, we first conducted a comprehensive expression profile of ZNF804A throughout glutamatergic cell development. Using two distinct differentiation protocols to generate hiPSC-derived cortical NPCs and postmitotic forebrain neurons in combination with RT-qPCR, ICC, and confocal microscopy, we identified immature postmitotic forebrain neurons taking on an upper layer identity as a suitable model to study ZNF804A gene function. Subsequently, we carried out CRISPR-Cas9 genome editing to excise exon 3 of ZNF804A, inducing mutations to the ZNF804A locus relevant to SZ. To account for potential genetic, epigenetic, and phenotypic heterogeneity within a cell culture before single-cell isolation, we validated two clones per ZNF804A mutation genotype (ZNF804A^+E3/+E3^, ZNF804A^+E3/−E3^, and ZNF804A^−E3/−E3^). Thereafter, we conducted bulk RNA-seq to determine transcriptional perturbations due to ZNF804A mutation. On the basis of these findings, we tested early immature synapses and pre- and postsynaptic protein expression in a compartment-specific automated analysis paradigm on confocal images of ZNF804A^+E3/+E3^, ZNF804A^+E3/−E3^, and ZNF804A^−E3/−E3^ glutamatergic neurons acquired with a high-content microscope. Further hypotheses were generated to test the involvement of ZNF804A in local translational control. These hypotheses were tested using mass spectrometry on neurite- and soma-specific proteomes as well as SUnSET to assess translational efficiency within dendrites of ZNF804A mutation cells. If not otherwise stated, no data were excluded. In Fig. 1B, NPCs derived from a given hiPSC line were considered to be biological replicates when they were generated from a different clone of a donor line. The rest of the study was carried out on CRISPR-Cas9–edited isogenic lines, with biological replicates considered to be independent when neurons were derived from genome edited hiPSC clones with a different passage number. Statistical analysis was carried out on averaged technical replicates to avoid pseudo-replication. All figure legends and/or figures themselves specify sample sizes and statistical tests used in the individual experiments. Detailed parameters of each experiment as well as analysis procedures can be found in the methodology sections below.

### Cell lines and hiPSC maintenance

HiPSC lines 014_CTM, M3_CTM, 127_CTM, 069_CTF, 007_CTF, and SCTi003-A (table S9) were generated and characterized previously (*60*, *65*, *66*): hiPSCs were generated from participants recruited, and methods were carried out in accordance with the “Patient iPSCs for Neurodevelopmental Disorders (PiNDs) study” (REC No 13/LO/1218). Informed consent was obtained from all participants for participation in the PiNDs study. Ethical approval for the PiNDs study was provided by the NHS Research Ethics Committee at the South London and Maudsley (SLaM) NHS R&D Office. The cell line SCTi003-A was purchased from STEMCELL Technologies (catalog no. 200-0511). Genetic editing was performed in a male neurotypical hiPSC line containing NGN2 under the control of an inducible, Tet-ON-controlled transgene overexpression system (NGN2-OPTi-OX). This cell line was a gift from M. Kotter (University of Cambridge) and has previously been described (*31*). HiPSCs were maintained in Geltrex-coated (Gibco; A1413302) plates in StemFlex media (Gibco; A3349401) under hypoxic conditions, media were changed every 48 hours, and cells were passaged at 70 to 90% confluency using Versene (Gibco; 15040066) and neuralized between passages 10 and 24.

### Cortical neural progenitor and neuron differentiation

To characterize ZNF8004A expression throughout NPC development, cortical NPCs were differentiated following a previously validated modified dual SMAD inhibition (SMADi) protocol (*67*). In brief, neuralization was induced in hiPSC cultures when they reached at least 90% confluency by adding neuralization medium containing small-molecule inhibitors LDN193189 (1 μM; Sigma-Aldrich; SML0559) and SB431542 (10 μM; Cambridge Bioscience; ZRD-SB-50). Neuralization medium was changed daily until D7, when cells were passaged (1:1) using StemPro Accutase (Gibco; A1110501) and 10 μM of Rho-associated, coiled-coil containing protein kinase (ROCK) inhibitor (ROCKi; Enzo Life Sciences, LKT-Y1000-M005) to a new plate, which contained NPC maintenance medium without SMADi. Further neuropassages were conducted on D12, D16, and D18.

To terminally differentiate the cells into postmitotic forebrain cortical neurons for long-read sequencing experiments, cells were passaged on D21 and plated on poly-d-lysine (25 μg/ml; PDL; Gibco, A3890401) in 1× borate buffer (Thermo Fisher Scientific; 28341)–laminin (10 μg/ml; Merck, L2020)–coated plates. The cells were treated with medium containing the Notch pathway inhibitor *N*-[2*S*-(3,5-difluorophenyl)acetyl]-l-alanyl-2-phenyl-1,1-dimethylethyl ester-glycine (DAPT; 10 μM; Merck, D5942) for 6 days daily before proceeding with half medium changes every 48 hours. RNA samples for long-read sequencing were collected on D18 and D30.

### Combining the OPTi-OX system with inhibitors to generate patterned forebrain neurons

The NGN2-OPTi-OX hiPSC line uses inducible NGN2 transgene overexpression for rapid neuronal differentiation; however, there is a concern regarding the potential heterogeneity of these neurons (*68*). To address this, a differentiation protocol was tailored for differentiation of more patterned forebrain neurons by combining inducible NGN2 forward programming with Wnt inhibition and SMADi (*32*).

Plates were coated with PDL (Millipore; A-003-E) in 1× borate buffer (Thermo Fisher Scientific; 28341) for 4 hours at 37°C, followed by laminin (Sigma-Aldrich; L2020) overnight at 37°C. The terminal plating process included rinsing hiPSCs with Hanks’ balanced salt solution, incubating them with Accutase (4 min), counting cells with an automated cell counter (TC10 Bio-Rad), and plating them at appropriate densities using D1 neuralization medium supplemented with 10 μM ROCKi, 2 μM doxycycline hyclate (dox; Sigma-Aldrich, D9891), SMADi (as above), and Wnt (2 μM; XAV939; Sigma-Aldrich, X3004) inhibitors to facilitate patterned differentiation into glutamatergic forebrain neurons. SMAD and Wnt inhibition were discontinued after D3, and the neurons were matured with daily medium changes involving 10 μM DAPT (Santa Cruz Biotechnology, SC-201315A), neurotrophic factors (brain-derived neurotrophic factor, 10 ng/ml, PeproTech, 450-02, and glial cell–derived neurotrophic factor, 10 ng/ml, PeproTech, 450–10), and B27-based maturation medium until D7. Daily media changes were conducted. Developing glutamatergic neurons were transfected with peGFP-N1 (BD Biosciences Clontech) using a calcium phosphate transfection approach. On D4, 1 μg of plasmid DNA was mixed with 1 M CaCl_2_ and HBS (50 mM Hepes buffer, 280 mM NaCl, and 1.5 mM Na_2_HPO_4_, pH 7.0); solution was allowed to precipitate for 30 min, before being added dropwise to D3 glutamatergic neurons. Cells were incubated with calcium phosphate/DNA mixture for 3 hours, before being replaced with B27-media with Ri; cells were fixed at D7.

For long-read sequencing experiments, ready-made glutamatergic neurons, derived from the NGN2-Opti-OX hiPSC line, were obtained (BitBio, io1001) and grown according to the manufacturer’s instructions. The RNA samples were collected on D3 of differentiation, which is an equivalent of D7 of NGN2-overexpression protocol when differentiating from hiPSC stage.

### Genome engineering

CRISPR, Cas9, and a dual sgRNA approach were chosen to excise exon 3 of *ZNF804A*. CRISPR RNA sequences (crRNA) were designed for sgRNAs to target either intron 2 or intron 3 of ZNF804A using the Benchling web tool (https://benchling.com/). Before their in vitro application, the specificity and efficiency of sgRNAs were evaluated using online resources CRISPOR (http://crispor.tefor.net) and IDT (https://eu.idtdna.com/pages) (table S10).

CRISPR-Cas9 complexes were delivered into the nuclei of OPTi-OX hiPSCs via nucleofection. Before this, cells were cultured on a Geltrex-coated 10-cm petri dish and allowed to proliferate in StemFlex medium until reaching 70% confluency. On the day of nucleofection, the CRISPR-Cas9 complex was formed following the manufacturer’s instructions. Briefly, crRNA:tracrRNA was annealed, added to Cas9 protein (2 μl), and incubated for 20 min to form the ribonucleoprotein complex. Thirty minutes before nucleofection, StemFlex was supplemented with RevitaCell (Gibco; A2644501; StemFlex:RC hereafter) to promote cell survival. For nucleofection, hiPSCs were incubated in Accutase (4 min). After trituration and cell straining (40 μm cell strainer, Falcon; 08-771-1), 1 × 10^6^ cells were added to 10 ml of StemFlex:RC, pelleted by 2 min centrifugation at 1250 rpm, and the nucleofection reaction was prepared using the Cell Line Nucleofector Kit V (Lonza; VCA-1003) as per the manufacturer’s instructions. The cell pellet was resuspended in nucleofection buffer, combined with the CRISPR-Cas9 construct and electroporation enhancer, and nucleofected using the Amaxa Nucleofector II and Nucleofector program B-016. Following nucleofection, cells were transferred to Synthemax (Corning; 734-2634)–coated six-well plates containing StemFlex:RC and incubated for 24 hours. A full-media change with StemFlex supplemented with CloneR (STEMCELL Technologies; 05888; 1:10 dilution) was performed once, and 24 hours later, CloneR was omitted from the medium to allow for hiPSC recovery. Media changes were carried out every 48 hours until cells reached 70% confluency.

To obtain a homogeneous cell culture with ZNF804A mutation genotypes, single-cell cloning was performed. Cells were transferred to two Synthemax-coated 10-cm dishes at low densities (500 and 1000 cells) using Accutase and 40-μm cell strainers. After single-cell passaging, hiPSCs were allowed to recover and form colonies in StemFlex supplemented with CloneR (1:10 dilution) as per the manufacturer’s instructions, to ensure robust cloning efficiency and single-cell survival. Subsequently, single cell–derived colonies were allowed to proliferate for a week with full media changes every 48 hours.

For clone picking, individual colonies were selected and transferred into a Geltrex-coated Nunc MicroWell 96-well plate (Thermo Fisher Scientific; 243656). Individual colonies, each originating from a single cell, were scraped using a P20 micropipette and transferred to separate wells containing 100 μl of Stemflex:RC in preparation for genotyping. After 24 hours, medium was fully replaced, excluding RC, and after a 2-day recovery period, the colonies were ready for genotyping.

To ensure monoclonality of heterozygous exon 3 excision ZNF804A^+E3/−E3^ lines, single-cell cloning and clone picking was performed a second time after identifying a promising clone. ZNF804A^+E3/−E3^_10.23 and _42.13 subclones were selected, which consistently showed dual bands in PCR gel.

### NMD assay

ZNF804A^+E3/+E3^ and mutant iPSCs were differentiated until D7, after which, cells were treated with either 40 μM anisomycin or vehicle for 6 hours. Cells were subsequently collected in TRIzol reagent (Thermo Fisher Scientific, 15596026) and RNA extracted and cDNA generated. RT-qPCR was performed on cDNA samples, with primers spanning exons 2 and 4 (table S1).

### Cellular compartment separation assay

Separation of neurites and somata was achieved by differentiating and maintaining neurons on Nunc Polycarbonate Cell Culture Inserts for six-well plates with a 3-μm pore size (Thermo Fisher Scientific; 140642). Before cell plating, the inserts were subjected to a bottom coating process involving the application of PDL in borate buffer (25 μg/ml), followed by laminin (20 μg/ml) only to the bottom of the membrane. This served as a guidance cue for the cells, facilitating the extension of their processes through the membrane toward the lower side. HiPSCs were terminally plated at a density of 500,000 cells per insert and differentiated into developing glutamatergic forebrain neurons using the protocol above. Media changes were performed daily by applying 2 ml to the bottom and 2 ml to the top of the insert until D7, when protein extraction was performed separately on neurites and somata.

Freshly prepared DIGE buffer (7 M urea, 2 M thiourea, and 30 mM tris base, pH 8.0) was used for protein extraction, with the procedure taking place on ice. Somata and neurites were extracted separately from the same membrane. First, membranes were transferred into clean six-well plates with ice-cold phosphate-buffered saline with magnesium and calcium (PBS-MC Gibco; 14040141). Somata were then dislodged from the upper surface of the membrane through gentle trituration with ice-cold PBS, transferred to a precooled microcentrifuge tube and pelleted at 2000*g* for 2 min at 4°C. Meanwhile, the remaining somata fractions were removed from the top of the membrane with a cotton-tip applicator, any excess PBS aspirated from the top and bottom sides, and the insert was then placed upside down on the bench. The membrane was excised from the insert using a sharp scalpel. It was then held bottom-up with forceps, and a cut was made from the circumference to the center using scissors. The resulting membrane piece was placed on top of a microcentrifuge tube. By applying gentle pressure with forceps, the center of the membrane was pushed down into the tube, creating a funnel shape until it was fully immersed in 150 μl of DIGE buffer. The tube was briefly vortexed, and after a 15-s spin, the membrane was discarded. Meanwhile, the centrifugation of the somata had completed, and the pelleted somata fractions were resuspended in 150 μl of DIGE buffer. Both fractions were then prepared for mass spectrometric analysis.

### RNA sequencing

Bulk RNA-seq was conducted on 18 samples, which included ZNF804A^+E3/+E3^, ZNF804A^+E3/−E3^, and ZNF804A^−E3/−E3^ lines (*n* = 2 clones per genotype and three differentiations per clone) at Genewiz Inc. in Azenta Life Sciences (South Plainfield, NJ). The libraries were prepared using the NEBNext Ultra II RNA Library Prep Kit for Illumina (NEB, Ipswich, MA, USA), and the preparation involved a polyA selection method. The libraries were quantified with a Qubit 4.0 Fluorometer (Life Technologies, Carlsbad, CA, USA) and assessed for RNA integrity using an RNA Kit on an Agilent 5300 Fragment Analyzer (Agilent Technologies, Palo Alto, CA, USA). The sequencing libraries were then multiplexed and loaded onto the Illumina NovaSeq 6000 instrument’s flow cell, and sequencing was performed with a 2 × 150 Pair-End (PE) configuration v1.5, as per the manufacturer’s instructions. Last, image analysis and base calling were carried out using the NovaSeq Control Software v1.7 on the NovaSeq instrument. Information about the number of reads and quality scores per sample can be found in table S11. The FastQ screen tool (*69*) was used to ensure quality control of the FASTQ files, which were then aligned to the human reference genome (GRCh38) using STAR (*70*). Subsequently, the aligned files were sorted based on chromosomal coordinates using the samtools sort function (*71*), and duplicate reads were identified using the Picard command MarkDuplicates (http://broadinstitute.github.io/picard/). A counts table, providing information about the number of aligned reads overlapping exons, was generated using the HTseq tool htseq-count (*72*). This table was used for downstream differential gene expression analysis (DGEA).

### Differential gene expression and gene set enrichment analyses

DGEA was performed on the transcriptomes from ZNF804A^+E3/+E3^, ZNF804A^+E3/−E3^, and ZNF804A^−E3/−E3^ lines using the default Wald test in the DEseq2 package with the following comparisons:

Signature A: “ZNF804A^+E3/+E3^ versus ZNF804A^−E3/−E3^”, ZNF804A^+E3/+E3^_4, ZNF804A^+E3/+E3^_61, *n* = 6 and ZNF804A^−E3/−E3^_20, ZNF804A^−E3/−E3^_44, *n* = 6. We used ZNF804A^+E3/+E3^ as baseline for DGEA.

Signature B: “ZNF804A^+E3/+E3^ versus ZNF804A^+E3/−E3^”, ZNF804A^+E3/+E3^_4, ZNF804A^+E3/+E3^_61, *n* = 6 and ZNF804A^+E3/−E3^_10.23, ZNF804A^+E3/−E3^_42.13, *n* = 6. We used ZNF804A^+E3/+E3^ as baseline for DGEA.

Signature C: “ZNF804A^+E3/−E3^ versus ZNF804A^−E3/−E3^”, ZNF804A^+E3/−E3^_10.23, ZNF804A^+E3/−E3^_42.13, *n* = 6 and ZNF804A^−E3/−E3^_20, ZNF804A^−E3/−E3^_44, *n* = 6. We used ZNF804A^+E3/−E3^ as baseline for DGEA.

DEGs were identified based on an adjusted *P* value threshold of <0.05 using the Benjamini-Hochberg method. Enrichment analyses were performed on DEGs using the DAVID Gene Functional Classification Tool (*36*). The DEG sets were tested against a physiologically relevant background, which included all genes meeting DESeq2’s internal filtering criteria for expression, i.e., those with adjusted *P* values not equal to NA. Significant terms were determined with an adjusted *P* value of <0.05.

To assess the overlap of genes affected by ZNF804A mutation with those in postmortem brain samples from SZ cases (*35*), Fisher’s exact tests were applied using GeneOverlap (https://bioconductor.org/packages/release/bioc/html/GeneOverlap.html), considering FDR correction with significance at FDR < 5%.

### Long-read sequencing

The library preparation for ONT targeted long-read sequencing was based on a reported protocol (*73*). One hundred nanograms of total RNA per sample was used for sequencing. Briefly, the samples were first reverse transcribed and barcoded with the NEBNext Single Cell/Low Input RNA Library Prep Kit for Illumina (New England BioLabs, E6420S/L) as per the manufacturer’s instructions, adapted for ONT library preparation. Barcoded and PCR-amplified samples were pooled based on concentration and region molarity to make up 1000 ng of cDNA in total before proceeding with target enrichment. The panel for targeted sequencing was designed using the IDT xGen Custom Hyb Panel Design Tool (https://eu.idtdna.com/pages/tools/xgen-hyb-panel-design-tool). The probes were designed for each exon of the genes in the panel. For genes with exons of more than 1000 bp, additional probes were designed to cover the length of the exon. The target enrichment was performed based on the manufacturer’s instructions. After target enrichment, the library for sequencing was prepared with ONT ligation sequencing kit (SQK-LSK114, ONT) and sequenced for 72 hours on ONT PromethION 24.

### Long-read data processing and analysis

The data processing and statistical analyses were run with King’s College London High Performance Computing cluster CREATE and R (v4.3.3). The samples were basecalled during sequencing with Dorado (v7.2.13) (ONT, https://github.com/nanoporetech/dorado) in super high accuracy mode. FASTQ pass files were used for downstream analyses. Porechop (v0.2.3) (https://github.com/rrwick/Porechop) was used to demultiplex reads and trim barcodes and sequencing adaptors, followed by Cutadapt (v4.2) (*74*) to trim the poly(A) tails. The demultiplexed reads were pooled together based on specific barcodes. This analysis was based on a previously reported pipeline (*75*). The FASTA files for individual barcodes were aligned to a human genome reference (GRCh38, Gencode release 44) with minimap2 (v2.24) (*76*, *77*) using the settings for ONT cDNA sequencing (−ax splice) and sorted with samtools (1.17) (*71*). To identify and quantify transcript isoforms, TALON (v5.0) (*78*) and Isoseq (v3.8.2) (Pacific Biosciences) were used. Before proceeding with isoform detection, the reads were first corrected with TranscriptClean (v2.0.3) (*79*). SQANTI3 (v4.3.1) was used to quality control isoforms, identified by TALON and Isoseq (*80*). Transcript tracks were plotted with ggtranscript (*81*).

### Western blotting

D7 neurons were collected in ice-cold radioimmunoprecipitation assay buffer, including protease and phosphatase inhibitors, followed by sonication (10 pulses at 40%). Protein concentrations were measured at 562 nm using the Pierce Bicinchoninic Acid kit (Thermo Fisher Scientific; 10678484). Proteins were prepared for SDS–polyacrylamide gel electrophoresis separation by denaturation with 2× Laemmli sample buffer (Bio-Rad Laboratories; 161-0737) with 355 mM β-mercaptoethanol for 5 min at 95°C.

Proteins (5 to 10 μg) were loaded onto self-made acrylamide gels (10 to 15%), separated at 100 V for approximately 90 min, and transferred onto polyvinylidene difluoride membranes (Bio-Rad; 1620177) at 78 mA overnight at 4°C. The membranes were blocked in 5% bovine serum albumin (Sigma-Aldrich; A7906) diluted in TBS-T solution, followed by primary antibody incubation overnight, washes in TBS-T, and secondary antibody incubation for 1 hour at room temperature. After a final wash, membranes were incubated in ECL Western Blotting Substrate (GE Healthcare; RPN2106) before imaging. See table S1 for antibody details.

Membrane images were analyzed using Image Studio Lite software version 5.2 (LICOR). The integrated density (ID) of each protein signal was measured and background corrected using the software’s automatic detection. Signal quantifications were processed in Microsoft Excel. The ID of each protein was normalized to the housekeeping gene (GAPDH) probed on the same blot. To calculate FC compared to wild types, these ratios were adjusted for potential batch effects by dividing them by the wild-type ratio from the same membranes. The normalization process followed the provided formulas, with ΔΔID representing the normalized ID value used for statistical analysis and data visualization

$$∆{\text{ID}}_{\text{target protein}}=\frac{{\text{ID}}_{\text{target protein}}}{{\text{ID}}_{\text{GAPDH or full protein}}}$$

$$∆{\mathrm{\Delta ID}}_{\text{target protein}}=\frac{{\mathrm{\Delta ID}}_{\text{target protein}}}{{\mathrm{\Delta ID}}_{\text{wildtype protein}}}$$

### In-gel digestion

For proteomic analysis, samples were prepared through a tryptic in-gel digestion as described before (*82*) with slight modifications. Briefly, 20 μg of protein was loaded on self-made acrylamide gels (12%) for neurites and somata fractions. After proteins were separated for 15 min at 50 V and 2 to 3 min at 180 V, protein bands were excised after staining with Coomassie Blue and destaining with 10% acetic acid. Afterward, the remaining dye was removed using repetitive washing steps with 50 mM ammonium bicarbonate and 25 mM ammonium bicarbonate/50% acetonitrile. Before tryptic digestion, destained gel pieces were dried in a Concentrator plus (Eppendorf) and afterward rehydrated with 50 mM ammonium bicarbonate. Afterward, 0.5 μg of trypsin (Serva) was added, and samples were incubated overnight at 37°C and 300 rpm. Digestion was stopped through the addition of trifluoric acid (TFA) and peptides were eluted through consecutive incubation steps with 50% acetonitrile/0.05% trifluoric acid. Resulting peptide solution was concentrated in a fresh Eppendorf tube and reconstituted in 20 μl of 0.1% TFA. This solution (0.5 μl) was afterward used for mass spectrometric measurements.

### Mass spectrometric analysis

The LC–MS/MS measurements were performed on an Ultimate 3000 RSLC nano LC system (Dionex) coupled to an Orbitrap Fusion Lumos Tribrid mass spectrometer (Thermo Fisher Scientific). Peptides were concentrated on a precolumn (Acclaim PepMap nanoViper, Thermo Fisher Scientific; 100 μm, 2 cm, 5 μm particle size) and washed for 7 min with 0.1% TFA at 30 μl/min. Subsequently, the precolumn was connected to an analytical C18 column (Acclaim PepMap nanoViper, Thermo Fisher Scientific; 75 μm, 50 cm, 2 μm particle size). Separation of peptides was performed at a flow rate of 400 nl/min with a gradient starting with 95% solution A (0.1% formic acid) and 5% solution B (84% acetonitrile, 0.1% formic acid). The concentration of solution B was increased up to 30% after 105 min, then within 2 min to 95%, and maintained at that level for an additional 3 min. Subsequently, the column was adjusted back to 5% solution B.

For DIA MS runs, the MS1 full scans were performed at a mass range of 350–1400 *m*/*z* (mass/charge ratio) with a resolution of 120,000 at 200 *m*/*z* (AGC 4 × 10^5^, 20 ms maximum injection time). Fragment analysis (MS2) was subdivided into 45 DIA isolation windows of different widths (10.5 to 268.4 *m*/*z* wide) using a resolution of 30,000 at 200 *m*/*z* (AGC 5 × 10^4^, 54 ms maximum injection time).

### Mass spectrometric data analysis

DIA data were analyzed with Spectronaut (Version 16.5, Biognosys) in a direct-DIA approach using the human reference proteome obtained from (*83*) using the following settings. Calibration was set to nonlinear iRT calibration with precision iRT enabled. Identification was performed using 1% *q* value cutoff on precursor and protein level whereas the maximum number of decoys was set to a fraction of 0.1 of library size. The mass tolerance for matching precursor and fragment ions was set to dynamic (default), which lets SN determine the optimal value. For quantification, interference correction was enabled with at least three fragment ions used per peptide; major and minor group quantities were set to mean peptide and mean precursor quantity, respectively, with top 3 group selection each. Quantity was determined on the MS2 level using area of XIC peaks with enabled cross run normalization. Proteomic data have been deposited to the ProteomeXchange Consortium via the PRIDE partner repository (*84*) with the dataset identifier PXD047788.

Before statistical analysis, the list of identified proteins was filtered based on NA values; only proteins that were identified in 70% of the samples were subjected to further analysis. Neurite- and soma-specific proteomes were assessed using the limma package’s integrated moderated *t* statistic and makeContrasts function with res = “detected proteins in neurite samples” − “detected proteins in soma samples”. Neurite-enriched proteins were identified based on an adjusted *P* value threshold of <0.05 using the Benjamini-Hochberg method and a log2 fold change of < −1.5. Soma-enriched proteins were identified based on the same adjusted *P* value threshold and a log2 fold change of >1.5. Neurite- and soma-enriched list of proteins were then subjected to enrichment analyses using the DAVID Gene Functional Classification Tool as described above. Of note here is that protein expression patterns across biological replicates within each fraction (*n* = 3 per genotype; two clones per genotype) were highly consistent, suggesting minimal cross-contamination or variability in the separation process.

### Surface sensing of translation

Protein synthesis rates were assessed in MAP2^+^ neurites of developing glutamatergic neurons using the SUnSET assay (*85*). Puromycin (Sigma-Aldrich; P8833) incorporation was validated at a 10 μg/ml concentration and puromycin was allowed to integrate into the newly forming polypeptide chain of proteins for 5 min before fixation, and an anti-puromycin antibody (Millipore; MABE343) was used to visualize the rate of protein synthesis through ICC.

### Immunocytochemistry

Cells were washed in PBS-MC and fixed using either double fixation with 4% paraformaldehyde (PFA) in 4% sucrose in PBS-MC (10 min) and 100% methanol on ice (10 min) or single fixation with 4% PFA in 4% sucrose only (SUnSET experiment). After fixation, cells were washed with PBS-MC, followed by incubation in a permeabilization and blocking solution containing Triton X-100 and 2% normal goat serum (Cell Signaling: 5425S) for 1 hour at room temperature. Primary antibodies were applied overnight at 4°C, followed by washing and incubation with Alexa Fluor secondary antibodies for 1 hour at room temperature. Cells were then stained with 4′,6-diamidino-2-phenylindole (DAPI) and cells/membranes on coverslips were mounted with ProLong Gold antifade mountant (Life Technologies: P36930). Antibodies are listed in table S1.

### Confocal microscopy

Fluorescent staining of cells and membranes on coverslips was visualized using a Leica SP5 confocal microscope equipped with 40× [numerical aperture (NA) 1.25] and 63× (NA 1.4) oil-immersion objectives, or a Nikon Spinning Disc Confocal equipped with a 60× (NA 1.4) oil-immersion objective and 405/488/594-nm lasers. LAS-AF software (v2.7.3) or NIS elements were used to capture Z-series images with a 0.5-μm Z-step. Images were processed using ImageJ version 2.1.0/1.53c. This involved maximum intensity projection, background subtraction using ImageJ’s built-in subtraction algorithm based on the “rolling ball” method, and adjustment of contrast and brightness to ensure consistent image quality for all images in the experiment.

### High-content imaging and automated image analysis

High-content imaging was performed on neurons grown on Nunc MicroWell 96-well optical-bottom plates with a coverglass base (Thermo Fisher Scientific; 164588) using an Opera Phenix High-Content Screening System (PerkinElmer, Waltham, MA) with a 20× water-immersion objective (NA 1.0). Immunofluorescence was captured using confocal imaging with 385-, 488-, 561-, and 640-nm lasers. An automated imaging script was generated, adjusting exposure times and laser powers for each channel and antibody using negative control wells containing DAPI-stained cells treated with primary or secondary antibodies only. Once generated, scripts remained consistent across all biological replications. Each plate contained neurons from a single clone per genotype. Each condition was imaged in technical triplicate, capturing 15 fields of view per well in z-stacks with three planes and 1.5-μm plane spacing. The acquired images were further processed using the Harmony High Content Imaging and Analysis Software.

An automated image analysis script was developed to analyze synaptic and ribosomal proteins. The script used max-projected images from a z-stack (three planes) as input and defined ROIs for separate analysis of protein puncta and mean intensities in somata, neurites, and whole cellular regions. It began by identifying DAPI^+^ cell nuclei and segmenting the MAP2^+^ areas around these nuclei, marking them as soma ROIs. Neurites were defined as ROIs based on the MAP2 channel and an algorithm by the Australian CSIRO research institute (www.csiro.au). Whole cell outlines were also marked as ROIs based on the MAP2 channel. The analysis script was tailored to detect puncta (0.4 to 4 μm) and to measure signal mean intensities separately within each ROI. To filter out erroneous puncta detections, a threshold was established using negative control wells to assess the mean intensity of background staining. Only puncta exceeding this threshold value were considered for analysis (fig. S13). For colocalization analysis, the script was instructed to identify positively colocalized puncta by overlaying the PSD95 channel onto the VGLUT1 channel. Puncta were counted as colocalized if a positively identified PSD95 punctum had at least 50% overlap with a positively identified VGLUT1 punctum. Puncta counts for each cellular region and mean intensities of puromycin staining within neurite ROIs were averaged per well and exported to Microsoft Excel for additional analysis.

For SUnSET analyses, FC in puromycin mean intensities were determined by dividing the MI of the mutant by the MI of the wild-type cells from the same plate. This was done to mitigate the influence of batch effects and technical variability

$$\text{FC}\;\text{from wild type}:∆{\text{MI}}_{\text{puromycin}}=\frac{{\text{MI}}_{\text{target puromycin}}}{{\text{MI}}_{\text{wildtype puromycin}}}$$

For synaptic and ribosomal proteins, puncta counts were initially normalized to ROI variable parameters, which included normalizing somatic puncta to somata counts, whole-cell puncta to MAP2^+^ cell counts, and neurite puncta to neurite lengths. This normalization process corrected for technical variations in automated analyses, such as varying numbers of detected somata in different wells. Subsequently, FCs were computed using the provided formulas (PC = puncta count)

Somata:

$$∆{\text{PC}}_{\text{target protein}}=\frac{{\text{PC}}_{\text{target protein}}}{\text{somata count}}$$

$$∆∆{\text{PC}}_{\text{target protein}}=\frac{∆{\text{PC}}_{\text{target protein}}}{∆{\text{PC}}_{\text{wild}-\text{type protein}}}$$

Neurites:

$$∆{\text{PC}}_{\text{target protein}}=\frac{{\text{PC}}_{\text{target protein}}}{\text{neurite length}}$$

$$∆∆{\text{PC}}_{\text{target protein}}=\frac{∆{\text{PC}}_{\text{target protein}}}{∆{\text{PC}}_{\text{wild}-\text{type protein}}}$$

Cells:

$$∆{\text{PC}}_{\text{target protein}}=\frac{{\text{PC}}_{\text{target protein}}}{\text{cell count}}$$

$$∆∆{\text{PC}}_{\text{target protein}}=\frac{∆{\text{PC}}_{\text{target protein}}}{∆{\text{PC}}_{\text{wild}-\text{type protein}}}$$

Statistical analysis was performed on averaged technical replicates to yield a single data point per biological replicate. Each biological replicate represented the averaged mean intensities or puncta counts of two to three wells. For better representation of data variability, technical replicates are plotted in raincloud plots.

### Calcium imaging and response quantification

Calcium imaging was performed in D7 neurons. Changes in intracellular calcium were quantified using baseline-normalized fluorescence (Δ*F*/*F*_0_). Stimulus onset was defined based on the time of maximal change in the mean Δ*F*/*F*_0_ trace, and baseline fluorescence was calculated from the prestimulus period. Responses were quantified within a 0- to 20-s poststimulus window for each ROI.

For each ROI, peak Δ*F*/*F*_0_ (maximum value within the response window), baseline-subtracted AUC (trapezoidal integration of Δ*F*/*F*_0_ minus baseline mean), peak latency (time from stimulus onset to peak), and rise time (10 to 90% of peak) were extracted. ROIs were classified as responders if peak Δ*F*/*F*_0_ exceeded baseline mean + 3× baseline SD.

### Gene module analysis

Gene expression modules were defined based on functional annotation of genes associated with calcium handling, synaptic organization, excitability, and neuronal activity (table S5). Module scores were calculated as the mean *z*-scored expression across all genes within each module for each sample. ZNF804A^+E3/+E3^ versus ZNF804A^−E3/−E3^ comparisons were performed using two-sided Wilcoxon rank-sum tests.

### Statistical analysis

All statistics were conducted in RStudio version 1.3.1093. Data normality was assessed through Shapiro-Wilk tests. Parametric tests were applied to normally distributed data, while nonparametric tests were used for data that did not follow a normal distribution. Nonparametric testing was carried out using Kruskal-Wallis tests and Dunn’s post hoc comparisons, while parametric testing was conducted using one-way/two-way ANOVA with Tukey HSD corrections or unpaired Student’s *t* tests. Statistical significance was determined based on *P* values and adjusted *P* values, with <0.05 considered significant. Effect sizes were determined for ANOVA and Kruskal-Wallis tests using R’s integrated functions that computed η^2^. The data were presented graphically through bar charts, boxplots, and raincloud plots using ggplot2. SD is indicated by error bars on the bar charts. For calcium imaging experiments, comparisons between ZNF804A^+E3/+E3^ and ZNF804A^−E3/−E3^ neurons were performed using two-sided Wilcoxon rank-sum tests for all continuous calcium response metrics. Individual ROIs were treated as independent observations. Exact *P* values are reported.
