# Supplementary material for: Schizophrenia risk gene ZNF804A controls ribosome localization and synaptogenesis in developing human neurons
Source: Sci Adv. 2026 May 20;12(21):eaea0755. doi: 10.1126/sciadv.aea0755 (PMC13189102; doi:10.1126/sciadv.aea0755)
Supplement: Supplementary file 1 — Supplementary Methods Supplementary Text Figs. S1 to S13 Legends for tables S1 to S12 [file sciadv.aea0755_sm.pdf]

Science Advances
AAAS

Supplementary Materials for

Schizophrenia risk gene ZNF804A controls ribosome localization and synaptogenesis in developing human neurons

Laura Sichlinger *et al.*

Corresponding author: Deepak P. Srivastava, deepak.srivastava@kcl.ac.uk

Sci. Adv. **12**, eaea0755 (2026)
DOI: 10.1126/sciadv.aea0755

The PDF file includes:

Supplementary Methods
Supplementary Text
Figs. S1 to S13
Legends for tables S1 to S12

Other Supplementary Material for this manuscript includes the following:

Tables S1 to S12

Supplemental Methods

Genotyping

Initial genotyping to identify potential CRISPR/Cas9 candidates involved PCR amplification using primers flanking the excision site, and DNA from crude cell lysates. A total of 96 clones were prepared for genotyping, where 20µl of each clone's cell suspension was placed in individual PCR tubes, and the remaining 40µl were evenly divided into two pre-warmed 96-well plates. Initial genotyping involved treating each clone's 20µl cell suspension with a PBS-based solution containing 0.5µl of proteinase K (20mg/ml; Promega; A505B), allowing cell lysis and genomic DNA (gDNA) extraction at 56°C for 1h, followed by stopping the reaction at 95°C for 10min, and using 1µl of crude lysates for PCR. 15 promising clones were expanded and underwent final genotyping by extracting gDNA using Promega ReliaPrep™ gDNA Tissue Miniprep System (A2051). To validate genotypes, PCRs were performed on extracted gDNAs from clones using two distinct primer pairs (**table S1**) and CloneAmp HiFi Polymerase (Takara; 639298).

Sanger sequencing

To confirm the precise excision sites, 5µl of PCR products and 5µl of forward primers (final concentration: 0.3µM; **table S1**) were submitted to Eurofins Genomics LLC or Source BioScience, and genotype was confirmed by aligning sequences to the amplicon sequence using the Clustal Omega online tool (https://www.ebi.ac.uk/Tools/msa/clustalo/). Sequencing was performed in duplicate using two distinct forward primers to validate the excision sites or wildtype genotypes.

Off-target analysis

Potential off-target sites for both sgRNAs were evaluated using IDT's CRISPR/Cas9 guide RNA design checker (https://www.idtdna.com/site/order/designtool/index/CRISPR_SEQUENCE). The top four sequences with the highest off-target cleavage risk were selected for further examination. Primers (**table S1**) were designed to target regions located approximately 250-850bp both up- and downstream of predicted off-target sites. PCR and Sanger sequencing were carried out as above.

Real-time quantitative PCR

RNA samples were collected in TRIzol reagent (Thermo Fisher Scientific, 15596026) and RNA was extracted using chloroform (372978, Sigma) and isopropanol as described before (Warre-Cornish et al., 2020). RNA purity was ensured through sodium acetate (Sigma, S2889) and 100% molecular grade ethanol treatments overnight, and the RNA samples were resuspended in RNase/DNase-free water and stored at -80°C. To analyze ZNF804A gene expression in NPCs, RNA was extracted at seven timepoints, totaling 92 samples, which included NPCs derived from three hiPSC lines and three clones per line (except for 014_01, which lacked a D20 sample due to technical issues). The mRNA extraction was carried out in nine batches, each time using samples from different donors to control for potential batch effects. mRNA from D7 neurons (n = 6) and CRISPR/Cas9 edited lines (n = 18) was extracted in two batches on the same day.

Reverse transcription to produce complementary DNA was conducted using SuperScript III (Invitrogen, 18080-044) and RT-qPCR was performed using MicroAmpTM Optical 384-well reaction plates (4343814, Applied Biosystems) and 5x HOT FIREPol® EvaGreen® qPCR Mix Plus (ROX) (final concentration 1X; Solis BioDyne 08-24-00020) and gene expression was measured by a QuantStudio7 Flex RT-qPCR system and QuantStudio RT-PCR software (Thermo Scientific, v1.3), based on a protocol optimised for the dyes and primers used (**table S1**). Each condition was assessed with three technical replicates, and the raw data for the cycle threshold (Ct) was analyzed using the $2^{-\Delta\Delta Ct}$ method to calculate the FC in gene expression relative to the control conditions (*54*).

Supplementary Text

Off-target analysis

We evaluated potential off-target effects. We confirmed pluripotency and self-renewal properties in the selected clones using RT-qPCR and immunocytochemistry (ICC) along with confocal microscopy (**fig. S5B**). Using *in silico* modeling to identify potential CRISPR/Cas9 off-target sites for both sgRNAs (**fig. S5C**), we selected the four most high-risk sequences for each sgRNA, isolated genomic DNA from the hiPSCs of each clone, and conducted Sanger sequencing of genomic regions spanning the potential off-target sites. We identified point mutations at the Cas9 cleavage site in off-target sequence 1 of sgRNA1 and off-target sequence 2 of sgRNA2. Specifically, within off-target sequence 1 of sgRNA1 cytosine was substituted for thymine (C → T) and within off-target sequence 2 of sgRNA2 thymine was substituted for guanine (T → G). Interestingly, these off-target effects were observed in all six clones (**fig. S5C**). Genomic sequences located both up- and downstream of each off-target substitution displayed no irregularities. In-depth

analysis of off-target sequence 1 of sgRNA1 indicated that it resides within a sizable non-coding region on chromosome 1, with the nearest coding region positioned over 17k nucleotides downstream (ring finger protein 220) and more than 32k nucleotides upstream (exoribonuclease family member 3) of the point mutation. Similarly, off-target sequence 2 of sgRNA2 is located in a vast intronic region on chromosome 20, with the closest downstream coding region being over 600k nucleotides away (Jagged1) and the nearest upstream coding region positioned more than 500k nucleotides away (BTB domain containing 3). Considering the significant distances to coding regions, the isolated editing effects, and the uniformity of the off-target effects among all clones, we proceeded with further investigations.

Supplemental Figures

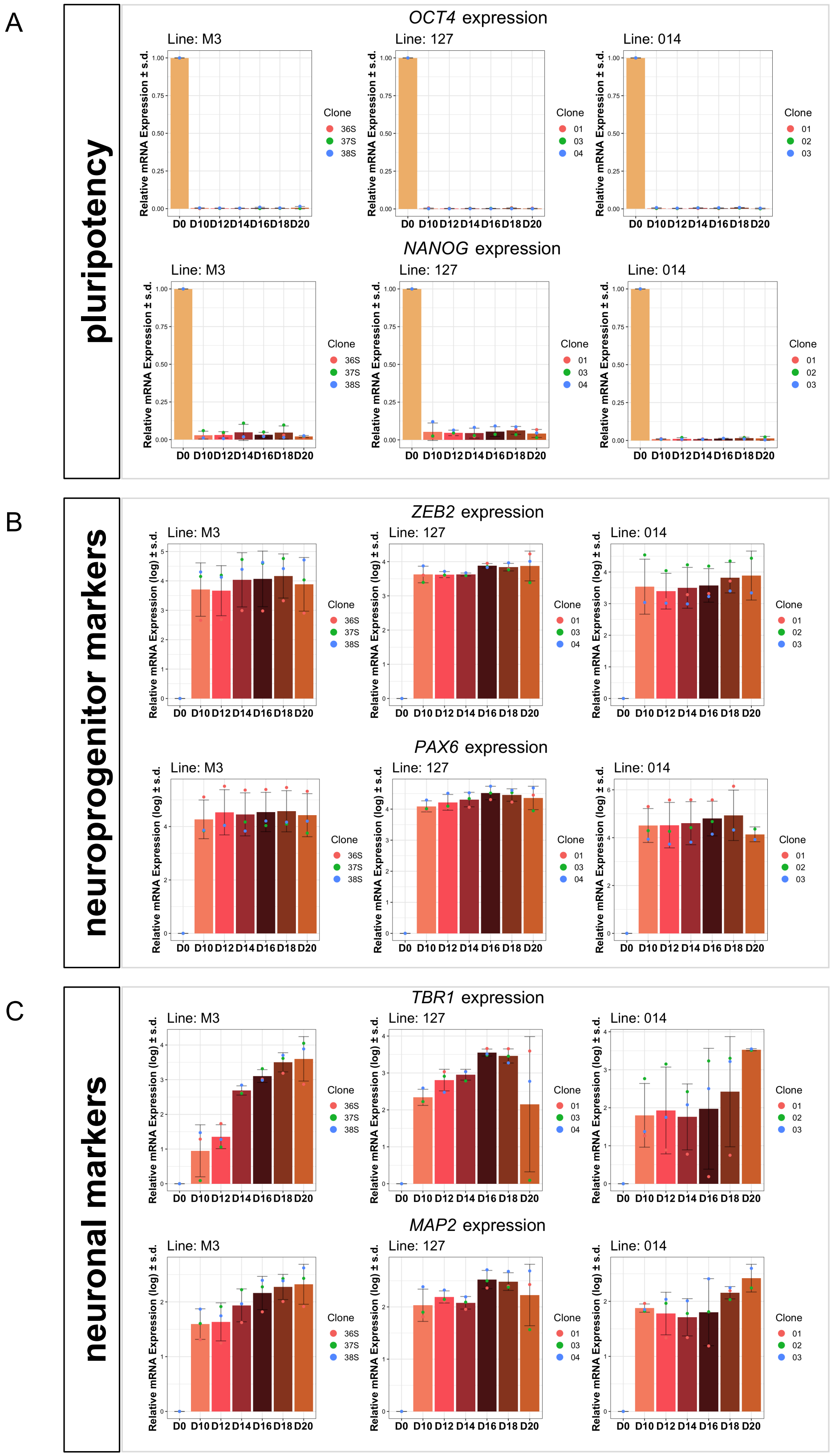

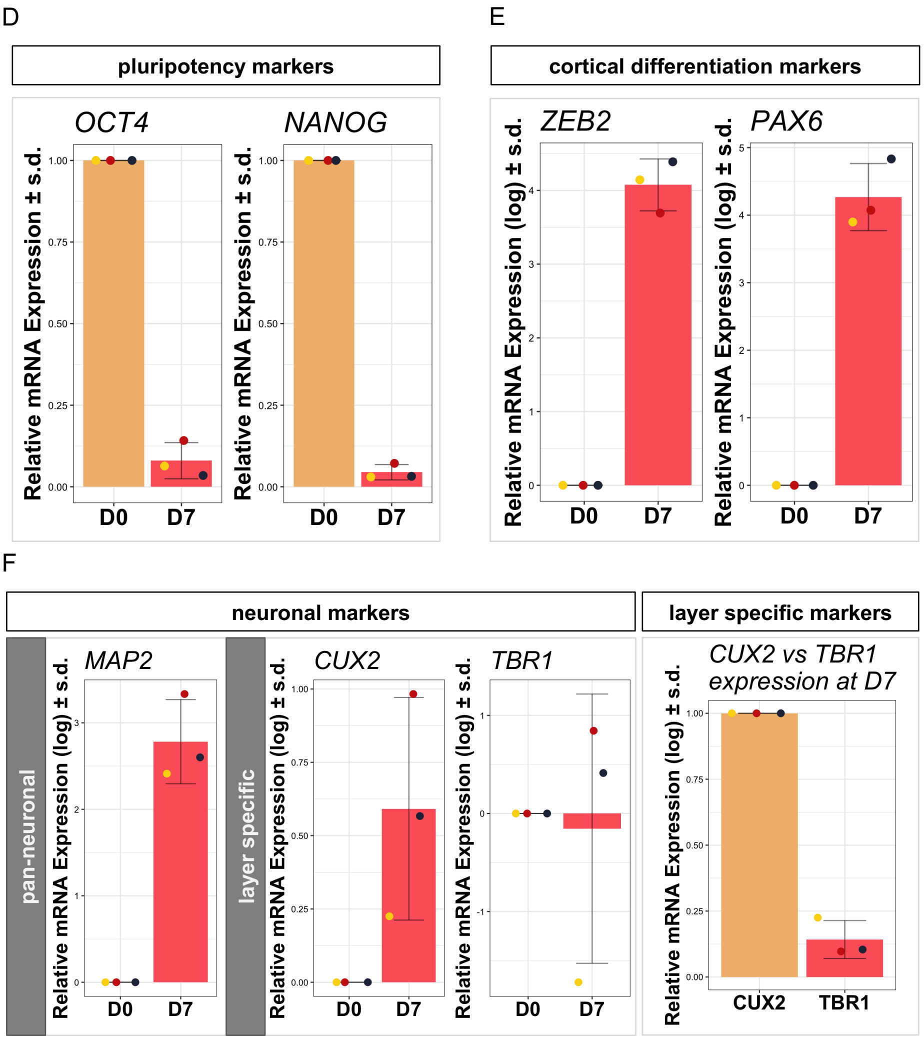

Fig. S1 Fate marker expression of cell lines at different developmental timepoints. Barcharts of RT-qPCR-derived log10-transformed $2^{-\Delta\Delta Ct}$ of (A) pluripotency markers octamer-binding transcription factor 4 (*OCT4*) and homeobox protein NANOG (*NANOG*), (B) neuroprogenitor cell (NPC) markers zinc finger E-box binding homeobox 2 (*ZEB2*) and paired box 6 (*PAX6*), and (C) neuronal markers T-box brain transcription factor 1 (*TBR1*) and microtubule associated protein 2 (*MAP2*) in each cell line used in this study (M3, 127 and 014) at seven timepoints between days (D)0-D20. Overlaying dotplots show colour-coded clones. Cells show expression profiles of NPCs appropriate to their developmental timepoint. (D – F) ioGlutamatergic cell-derived neurons resemble

immature upper layer cortical neurons. Barcharts of RT-qPCR-derived log10-transformed $2^{-\Delta\Delta Ct}$ of (D) pluripotency markers *OCT4* and *NANOG* (B) NPC markers *ZEB2* and *PAX6,* and (C) pan-neuronal marker *MAP2*, layer VI specific marker *TBR1*, and upper layer specific maker cut like homebox 2 (*CUX2*) in hiPSCs (D0) and developing neurons seven days post neural induction (D7). Biological replicates (i.e. distinct differentiations) are plotted in overlaying dotplots. High *MAP2* transcript abundance at D7 indicates post-mitotic neuronal fate. Biological replicates showed variable layer-specific marker expression; *CUX2* expression was more abundant compared to *TBR1* expression at D7. Error bars: mean ± standard deviation (SD)

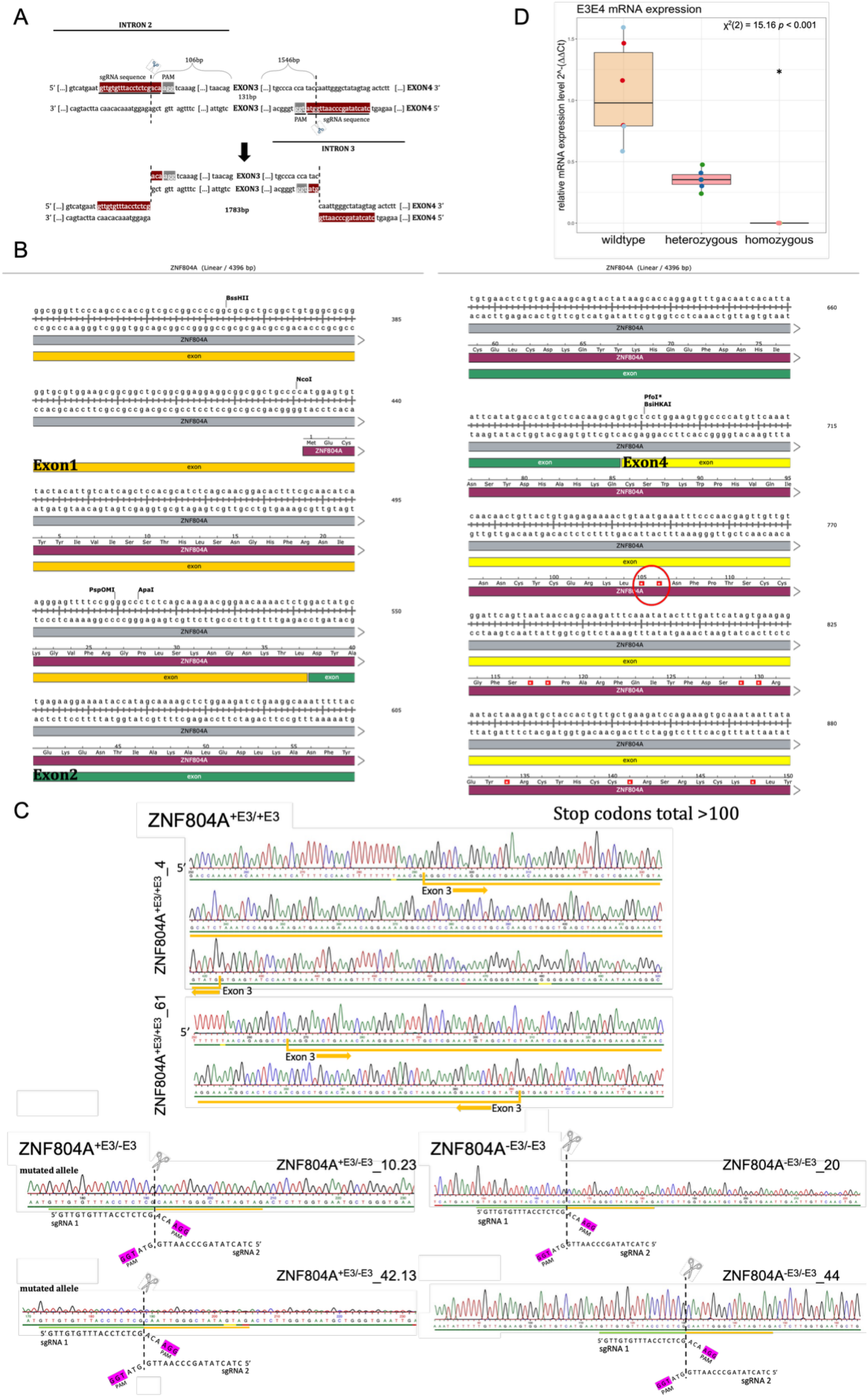
A
INTRON 2
106bp
1546bp
sgRNA sequence
PAM
EXON3
131bp
EXON4 3'
EXON4 5'
sgRNA sequence
INTRON 3
1783bp
B
ZNF804A (Linear / 4396 bp)
BssHII
NcoI
Exon1
PspOMI
ApaI
Exon2
PfoI*
BsiHKAI
Exon4
Stop codons total >100
C
ZNF804A+E3/+E3
ZNF804A+E3/+E3_4
ZNF804A+E3/+E3_61
Exon 3
ZNF804A+E3/-E3
mutated allele
ZNF804A+E3/-E3_10.23
ZNF804A+E3/-E3_42.13
ZNF804A-E3/-E3
ZNF804A-E3/-E3_20
ZNF804A-E3/-E3_44
sgRNA 1
sgRNA 2
PAM
D
E3E4 mRNA expression
χ2(2) = 15.16 p < 0.001
relative mRNA expression level 2^-(ΔΔCt)
wildtype
heterozygous
homozygous

Fig. S2 Validation of ZNF804A mutation model. (A) Schematic of excision site (1783 base pairs (bp)) of dual sgRNA approach; sgRNAs induced double strand breaks within either intron 2 (sgRNA1) or intron 3 (sgRNA2). (B) *In silico* design of mutant *ZNF804A* model. SnapGene (www.snapgene.com) modelling of exon 3 excision of *ZNF804A* showing putative premature stop codons in exon 4 (first stop condons marked with red circle; in total > 100). (C) Sanger sequences of ZNF804A mutant clones. Exon 3 excision (heterozygous: ZNF804A$^{+E3/-E3}$_10.23, ZNF804A$^{+E3/-E3}$_42.13; homozygous: ZNF804A$^{-E3/-E3}$_44, ZNF804A$^{-/-}$_20.) or unaltered genotype (wildtype clones ZNF804A$^{+E3/+E3}$_4, ZNF804A$^{+/+}$_61) was confirmed by sanger sequencing with two distinct forward primers. (D) mRNA validation of ZNF804A mutant clones. Boxplots showing RT-qPCR-derived log10-transformed $2^{-\Delta\Delta Ct}$ values of schizophrenia risk variant of zinc-finger protein 804A (*ZNF804A*), *ZNF804A ^{E3E4},* in each clone of three genotypes (ZNF804A$^{+E3/+E3}$, ZNF804A$^{+E3/-E3}$, ZNF804A$^{-E3/-E3}$) in D7 developing glutamatergic neurons (n = 18; 3 individual differentiations per clone). Clones are plotted in overlaying dotplots.

* p.adj. < 0.05.

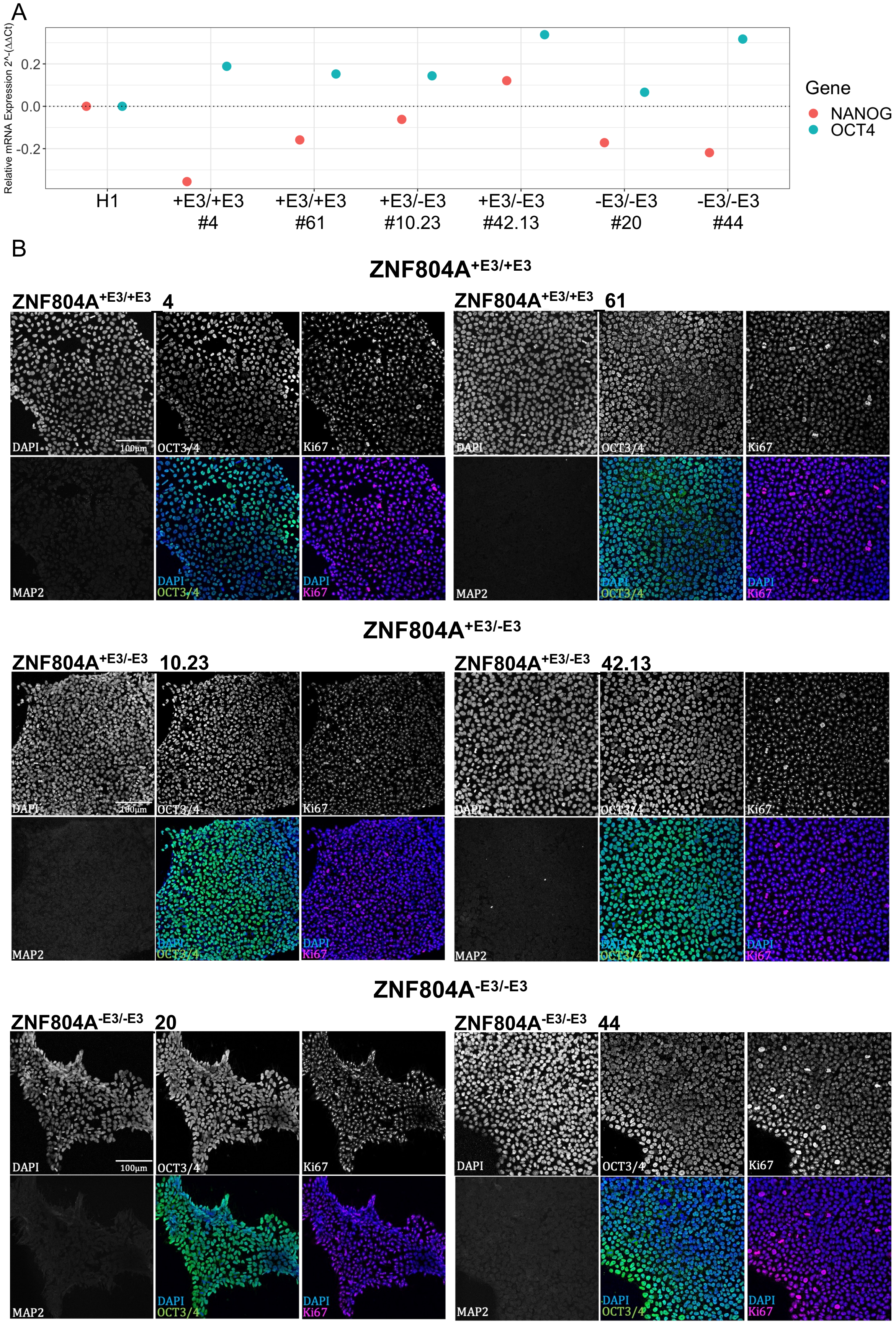
A
Relative mRNA Expression 2^-(ΔΔCt)
0.2
0.0
-0.2
H1
+E3/+E3 #4
+E3/+E3 #61
+E3/-E3 #10.23
+E3/-E3 #42.13
-E3/-E3 #20
-E3/-E3 #44
Gene
NANOG
OCT4
B
ZNF804A+E3/+E3
ZNF804A+E3/+E3 4
ZNF804A+E3/+E3 61
ZNF804A+E3/-E3
ZNF804A+E3/-E3 10.23
ZNF804A+E3/-E3 42.13
ZNF804A-E3/-E3
ZNF804A-E3/-E3 20
ZNF804A-E3/-E3 44
DAPI
OCT3/4
Ki67
MAP2
100μm

C

Off-target analysis

sgRNA sequence 1 - Intron 2<br>**GTTGTGTTTACCTCTCGACA**		
**Potential off-target sequence**		**Locus**
1	*AATGTGTGTACCTCTTGACA*	chr1:-44387600
2	*TTTCTGTTTACCTCTCTACA*	chr7:+122403326
3	*ATTGTGTGTGCCTCTTGACA*	chr5:+110971250
4	*CTTGAGTTTTCCTCTCTACA*	chr3:-117476849

sgRNA sequence 2 - Intron 3<br>**CTACTATAGCCCAATTGGTA**		
**Potential off-target sequence**		**Locus**
1	*CACCTA-AGCCCAATTGGTA*	chr12:+111474134
2	*CTTCTATA-CCCAATTGGTA*	chr20:+11338400
3	*CTTCTAT-GCCTAATTGGTA*	chr20:+23807904
4	*TTACTAG-GCCCAATTGGTA*	chr20:-39488186

ZNF804A $^{+E3/+E3}$

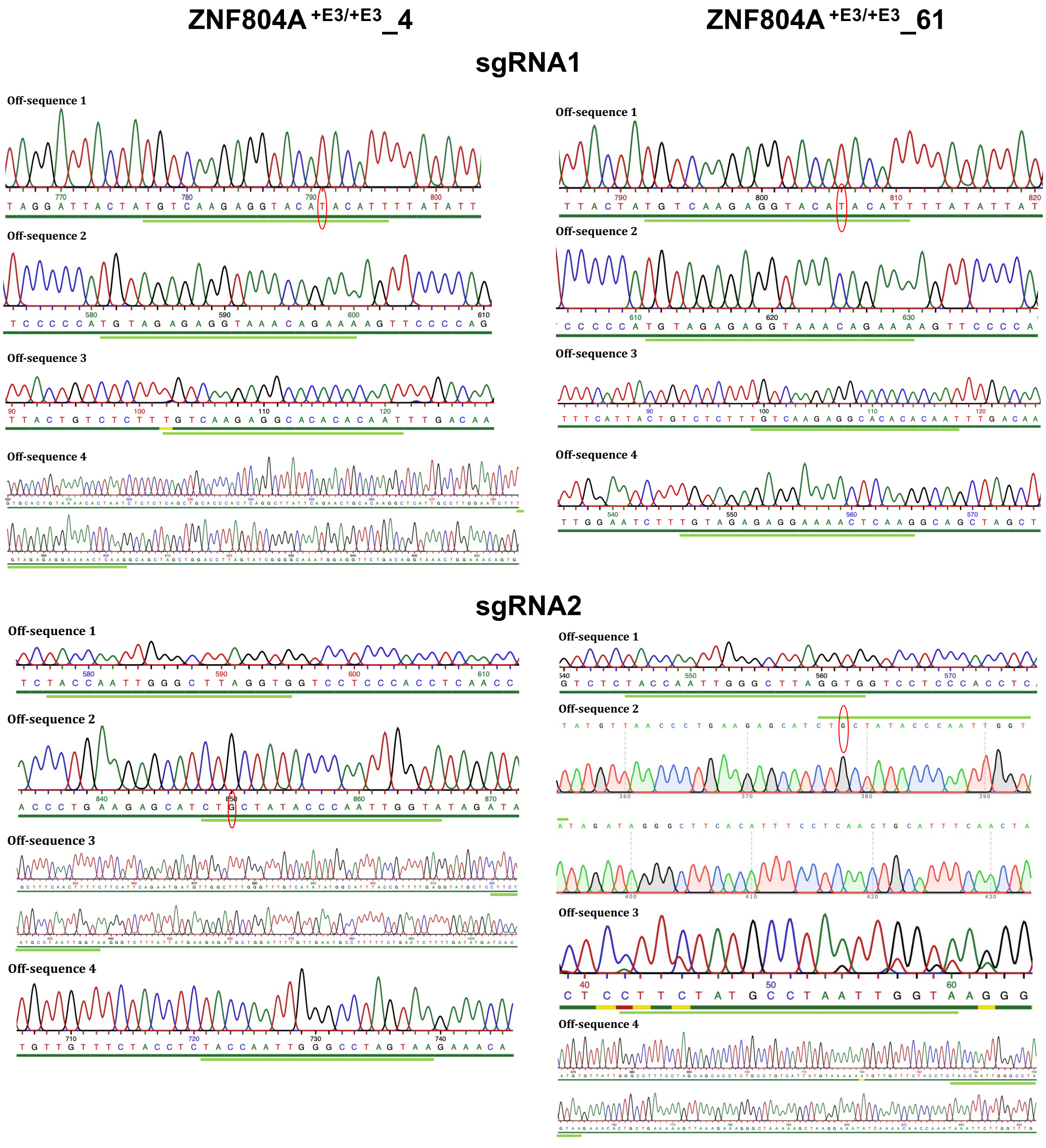

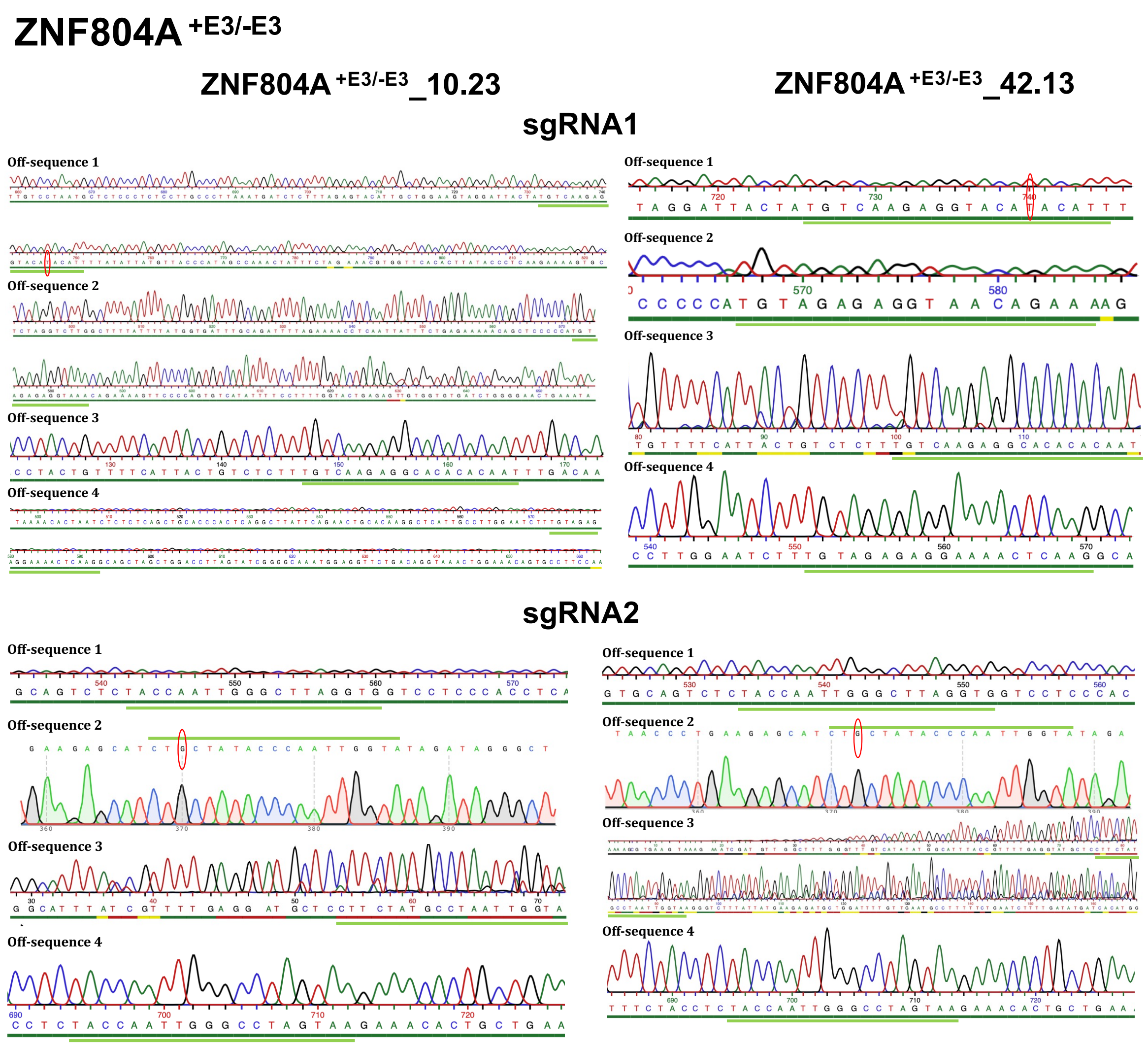
ZNF804A +E3/-E3
ZNF804A +E3/-E3_10.23
ZNF804A +E3/-E3_42.13
sgRNA1
Off-sequence 1
Off-sequence 2
Off-sequence 3
Off-sequence 4
Off-sequence 1
Off-sequence 2
Off-sequence 3
Off-sequence 4
sgRNA2
Off-sequence 1
Off-sequence 2
Off-sequence 3
Off-sequence 4
Off-sequence 1
Off-sequence 2
Off-sequence 3
Off-sequence 4

ZNF804A $^{-E3/-E3}$

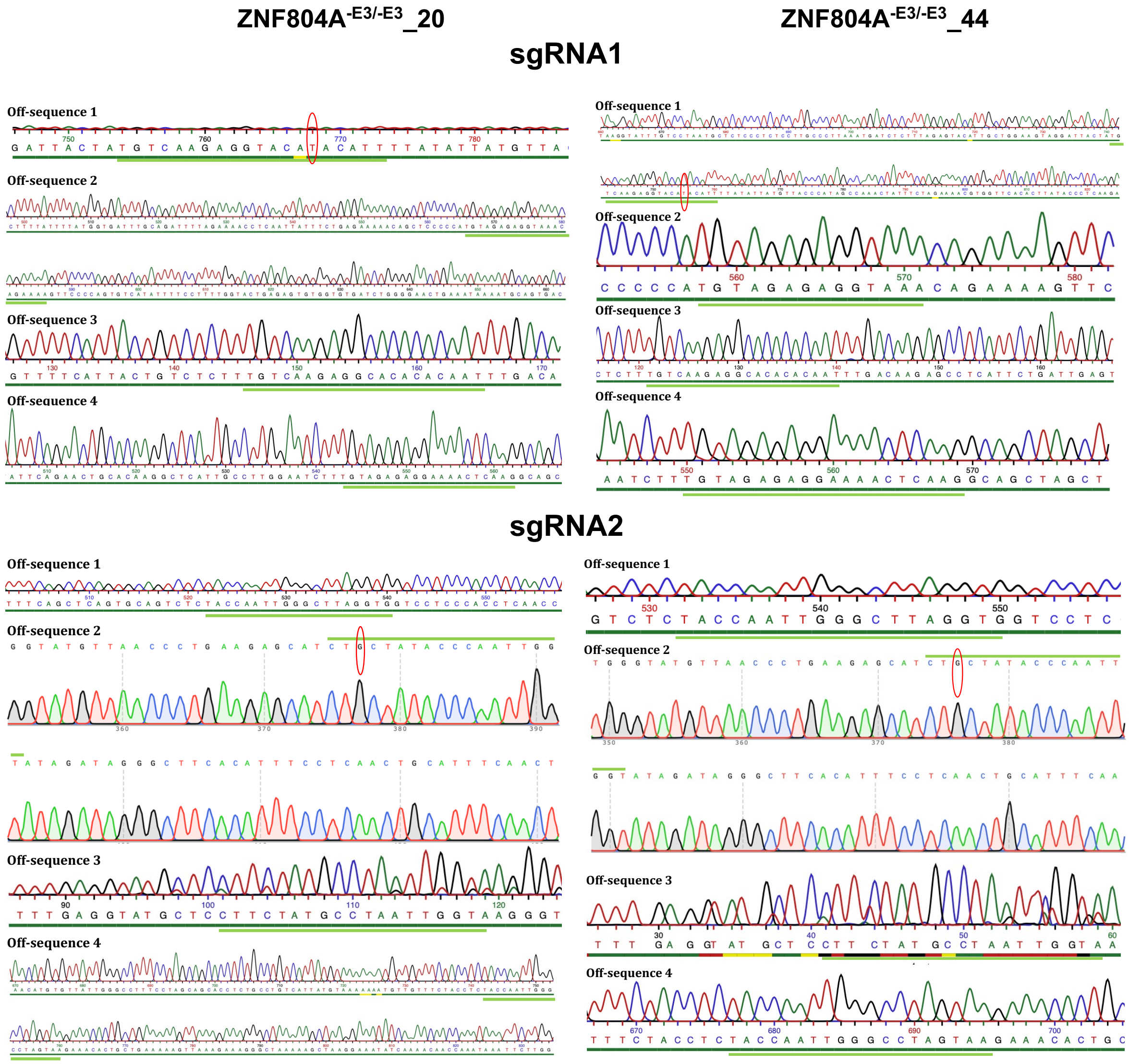

D

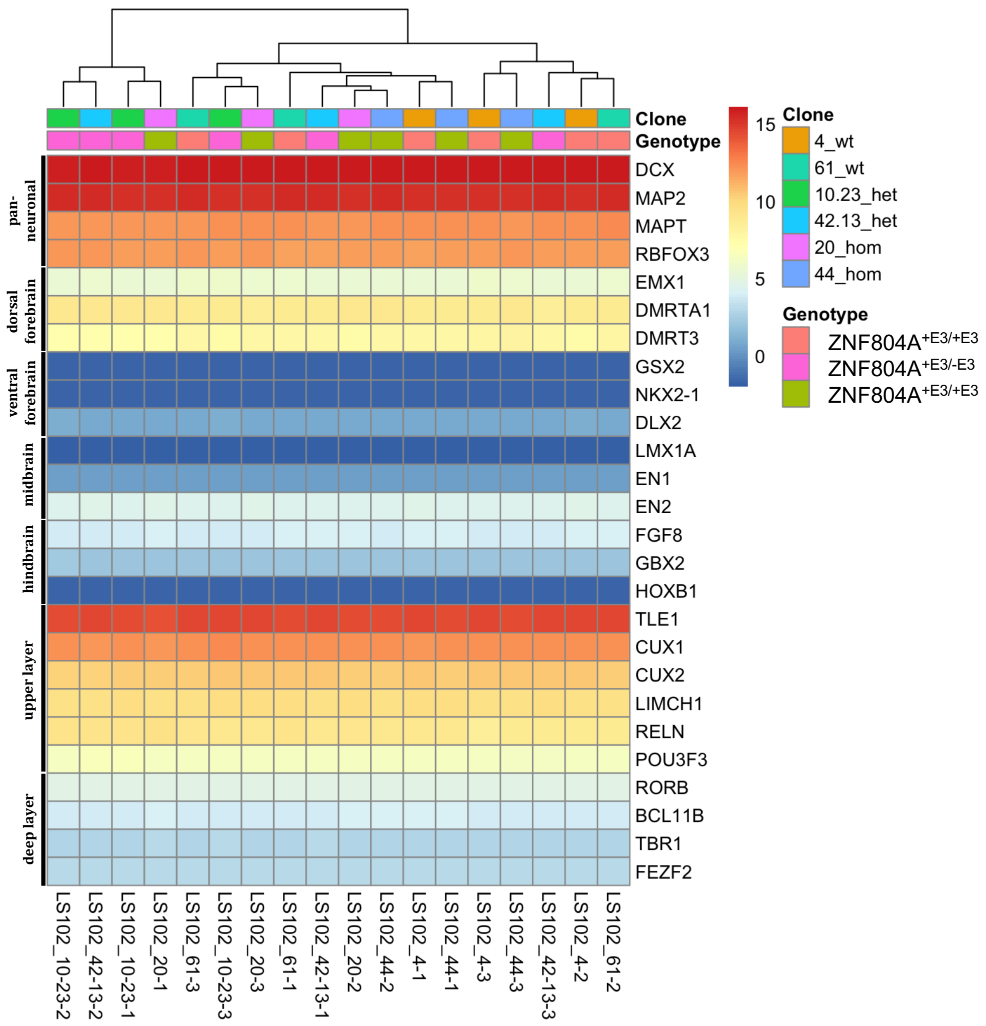

Fig. S3 Validation of cell line integrity and gene editing specificity. (A-B) Genome editing did not interfere with pluripotency properties. (A) Dotplot of RT-qPCR-derived $2^{-\Delta\Delta Ct}$ of values pluripotency markers octamer-binding transcription factor 4 (*OCT4*) and homeobox protein NANOG (*NANOG*) in human induced pluripotent stem cells (hiPSCs) of each genome edited (heterozygous: ZNF804A$^{\mathbf{+E3/-E3}}$_10.23, ZNF804A$^{\mathbf{+E3/-E3}}$_42.13; homozygous: ZNF804A$^{\mathbf{-E3/-E3}}$_44, ZNF804A$^{\mathbf{-E3/-E3}}$_20.) or wildtype clone (wildtype: ZNF804A$^{\mathbf{+E3/+E3}}$_4, ZNF804A$^{\mathbf{+E3/+E3}}$_61) compared to the expression in a male human embryonic stem cell line (hESC – H1). High expression of pluripotency markers, similar

to H1 line, indicate clones retained pluripotency properties. (B) Representative confocal images of hiPSCs from each clone. Immunostaining shows pluripotency marker OCT3/4 (green), proliferation marker Ki67 (magenta), and pan-neuronal marker microtubule-associated protein 2 (MAP2). Nuclei were stained with DAPI (blue). Global expression of OCT3/4 and Ki67 confirm unaltered hiPSC properties. MAP2 was not expressed. (C) Potential off-target effect screening of gene edited clones. Top four predicted off-target sites of each single guide RNA (sgRNA) (table) were tested in clones using sanger sequencing. Sanger sequences show clear sequences except for two substitutions in off-target sequence 1 of sgRNA1 (C → T) and off-target sequence 2 of sgRNA2 (T → G). Off-target effects were isogenic. Substitutions are indicated by red circle and predicted off-target sites are indicated by green line. (D) Heatmap showing transcriptomic signature of D7 neurons derived from all cell lines closely resemble developing glutamatergic forebrain neurons as they mature into upper-layer cortical cells. Log-transformed expression of DESeq2 normalized counts of markers associated with specific cell types is plotted using colour key scaled from 15 to 0 with 15 (red) representing high gene expression and 0 (dark blue) representing low gene expression. There is no discernible clustering based on clone or genotype.

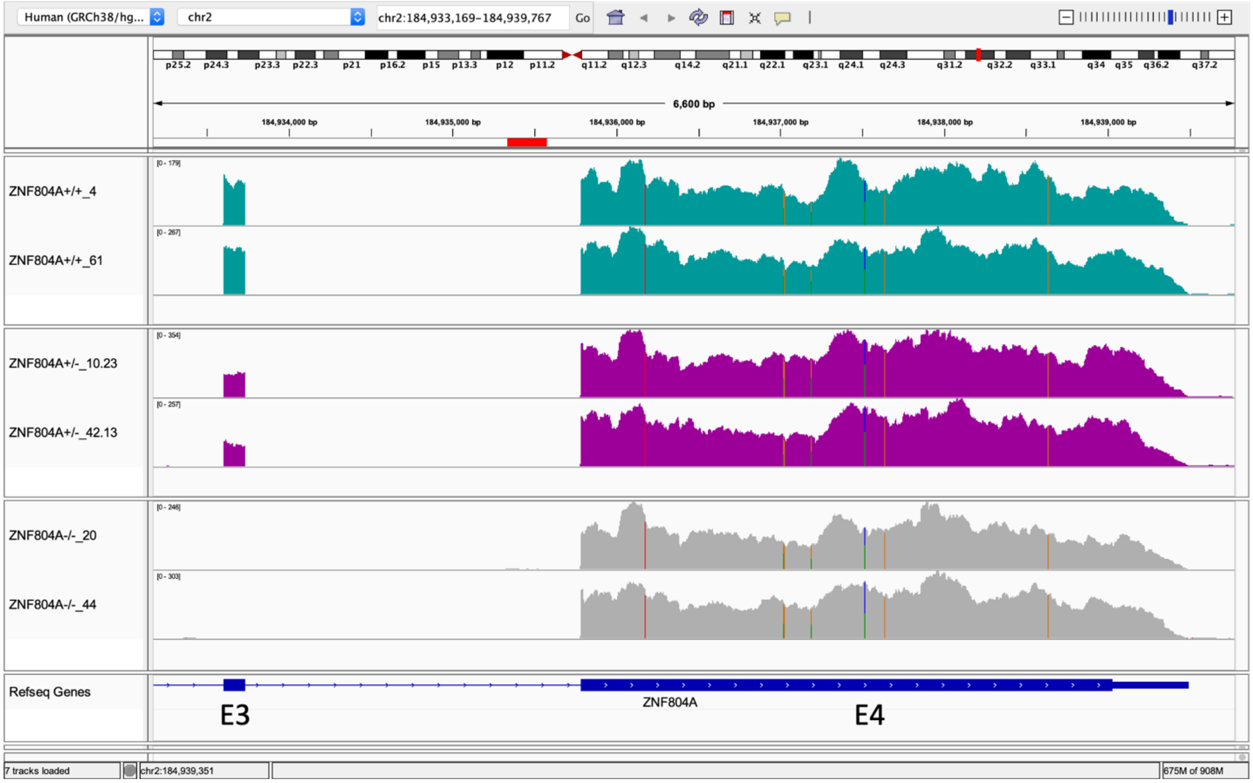

Fig. S4 Integrative genome viewer (IGV) tracks of each ZNF804A mutant clone at 6000 base pair resolution. Representative alignments of each clone; wildtype alignments ($ZNF804A^{+E3/+E3}$_4 and _61) are shown as turquois, heterozygous alignments ($ZNF804A^{+E3/-E3}$_10.23 and _42.13) as magenta and homozygous alignments ($ZNF804A^{-E3/-E3}$_20 and _44) as grey polygons. Reference genome sequence of ZNF804A is plotted on the bottom in blue. Exon 3 deletion was confirmed in mutation clones, whereas exon 4 seems to be transcribed.

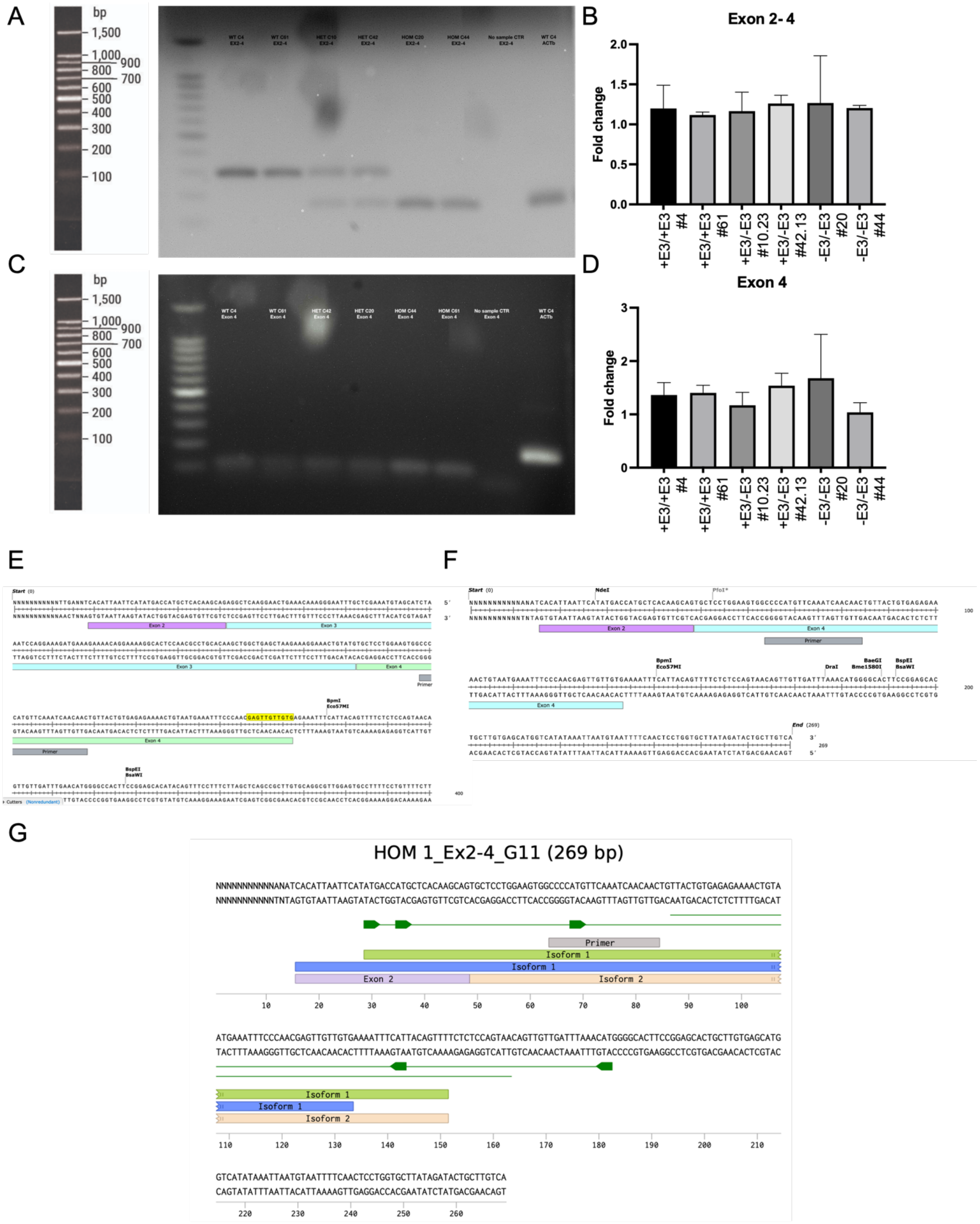

Fig. S5. PCR analysis of cDNA from D7 neurons confirms presence of exon 3 skipping transcript in ZNF804A mutant lines. (A & C) Representative PCR plots of

primers spanning exon 2 to exon 4 and primers targeting exon 4 of all ZNF804A mutation and control lines. ACTB expression in ZNF804A$^{+E3/+E3}$ control line was used as a positive control. (B & D) Bar chart of RT-qPCR-derived log10-transformed $2^{-\Delta\Delta Ct}$ values of exon 2-4 and exon 4 primers of all ZNF804A mutant and control lines. (E-F) Sanger sequences of ZNF804A mutant and control lines confirming a mutant exon 3 skipping transcript in mutant lines. (G) Sanger sequencing of cDNA from day 7 homozygous (ZNF804A$^{-E3/-E3}$) neurons confirmed the presence of an exon 2-4 fusion amplicon and sequence consistent with two fusion transcript variants (annotated as Isoform 1 and Isoform 2).

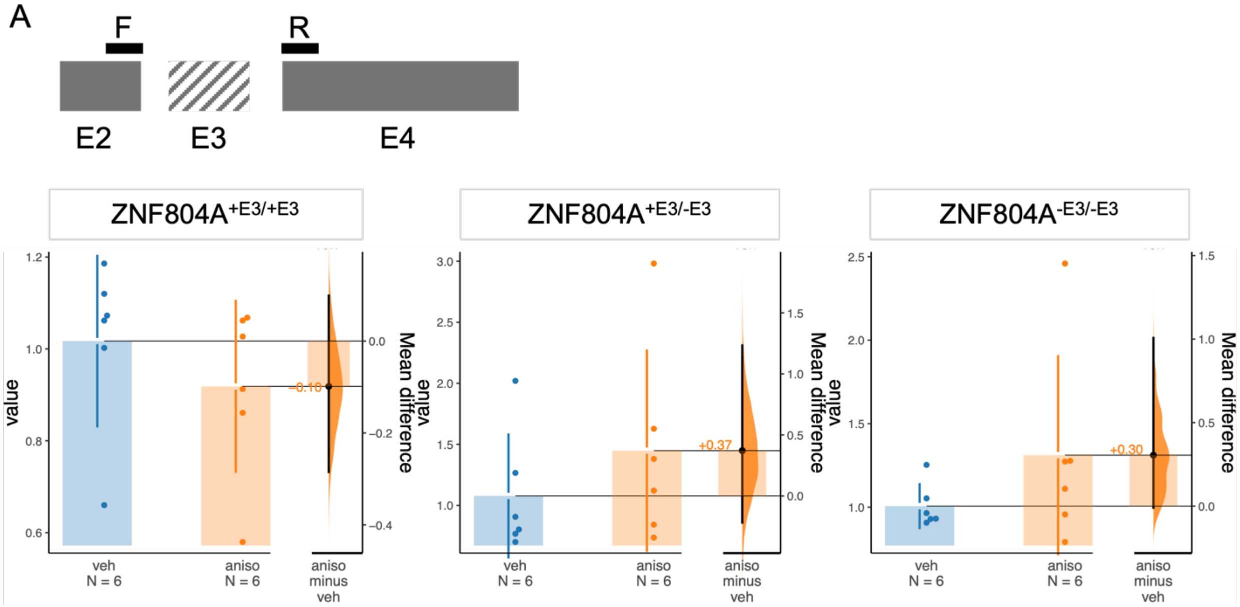

Fig S6. Exon 2-4 fusion transcript show nonsense mediated decay sensitivity. (A) Gardner-Altman estimation plots showing genotype-dependent effects of nonsense-mediated decay (NMD) inhibition of truncated isoform expression. While NMD inhibition had little to no effect, with mean differences close to zero and confidence intervals overlapping zero, in ZNF804A$^{+E3/+E3}$ neurons, both ZNF804A$^{+E3/-E3}$ and ZNF804A-$^{E3/-E3}$ conditions showed a consistent positive shift following anisomycin treatment (aniso), reflected by increased group means and positive bootstrapped mean differences.

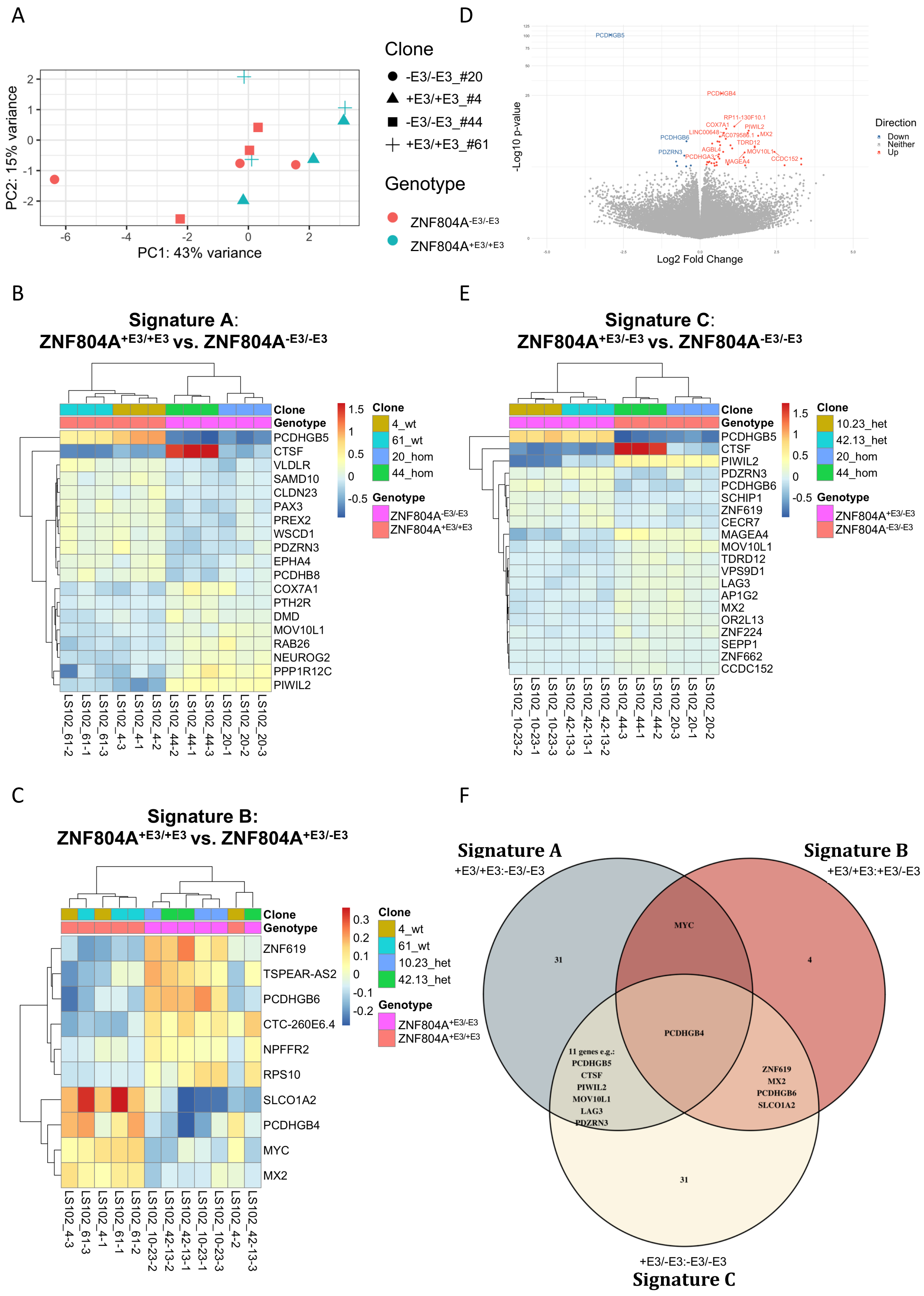

A
PC2: 15% variance
PC1: 43% variance
Clone
-E3/-E3_#20
+E3/+E3_#4
-E3/-E3_#44
+E3/+E3_#61
Genotype
ZNF804A-E3/-E3
ZNF804A+E3/+E3
D
-Log10 p-value
Log2 Fold Change
Direction
Down
Neither
Up
PCDHGB5
PCDHGB4
PCDHGB6
PDZRN3
B
Signature A:
ZNF804A+E3/+E3 vs. ZNF804A-E3/-E3
Clone
Genotype
PCDHGB5
CTSF
VLDLR
SAMD10
CLDN23
PAX3
PREX2
WSCD1
PDZRN3
EPHA4
PCDHB8
COX7A1
PTH2R
DMD
MOV10L1
RAB26
NEUROG2
PPP1R12C
PIWIL2
4_wt
61_wt
20_hom
44_hom
E
Signature C:
ZNF804A+E3/-E3 vs. ZNF804A-E3/-E3
PCDHGB5
CTSF
PIWIL2
PDZRN3
PCDHGB6
SCHIP1
ZNF619
CECR7
MAGEA4
MOV10L1
TDRD12
VPS9D1
LAG3
AP1G2
MX2
OR2L13
ZNF224
SEPP1
ZNF662
CCDC152
10.23_het
42.13_het
20_hom
44_hom
C
Signature B:
ZNF804A+E3/+E3 vs. ZNF804A+E3/-E3
ZNF619
TSPEAR-AS2
PCDHGB6
CTC-260E6.4
NPFFR2
RPS10
SLCO1A2
PCDHGB4
MYC
MX2
4_wt
61_wt
10.23_het
42.13_het
F
Signature A
+E3/+E3:-E3/-E3
Signature B
+E3/+E3:+E3/-E3
MYC
31
4
PCDHGB4
11 genes e.g.:
PCDHGB5
CTSF
PIWIL2
MOV10L1
LAG3
PDZRN3
ZNF619
MX2
PCDHGB6
SLCO1A2
31
+E3/-E3:-E3/-E3
Signature C

Fig. S7 Transcriptomic analysis of novel ZNF804A partial loss-of-function model. (A) Principal component analyses (PCA) plot of ZNF804A$^{+/+}$ and ZNF804A$^{-/-}$ samples. PC1 (43%) is plotted on x-axis, PC2 (15%) is plotted on y-axis. Color-coding according to genotype, shape-coding according to shapes. Samples cluster slightly according to ZNF804A$^{+E3/+E3}$ and ZNF804A$^{-E3/-E3}$ genotypes. Supervised heatmaps of top protein coding DEGs of Signature A (B), B (C) and C (E). Dendrogram clustering occurs according to genotype. (D) Volcano plot of differentially expressed genes (DEGs; x-axis= log2 fold change, y-axis = -log10 p-value). Significantly up-regulated DEGs (FDR < 0.05) depicted in red, significantly down-regulated DEGs in blue. (F) Overlapping differentially expressed genes of each Signature plotted in Venn diagram.

A

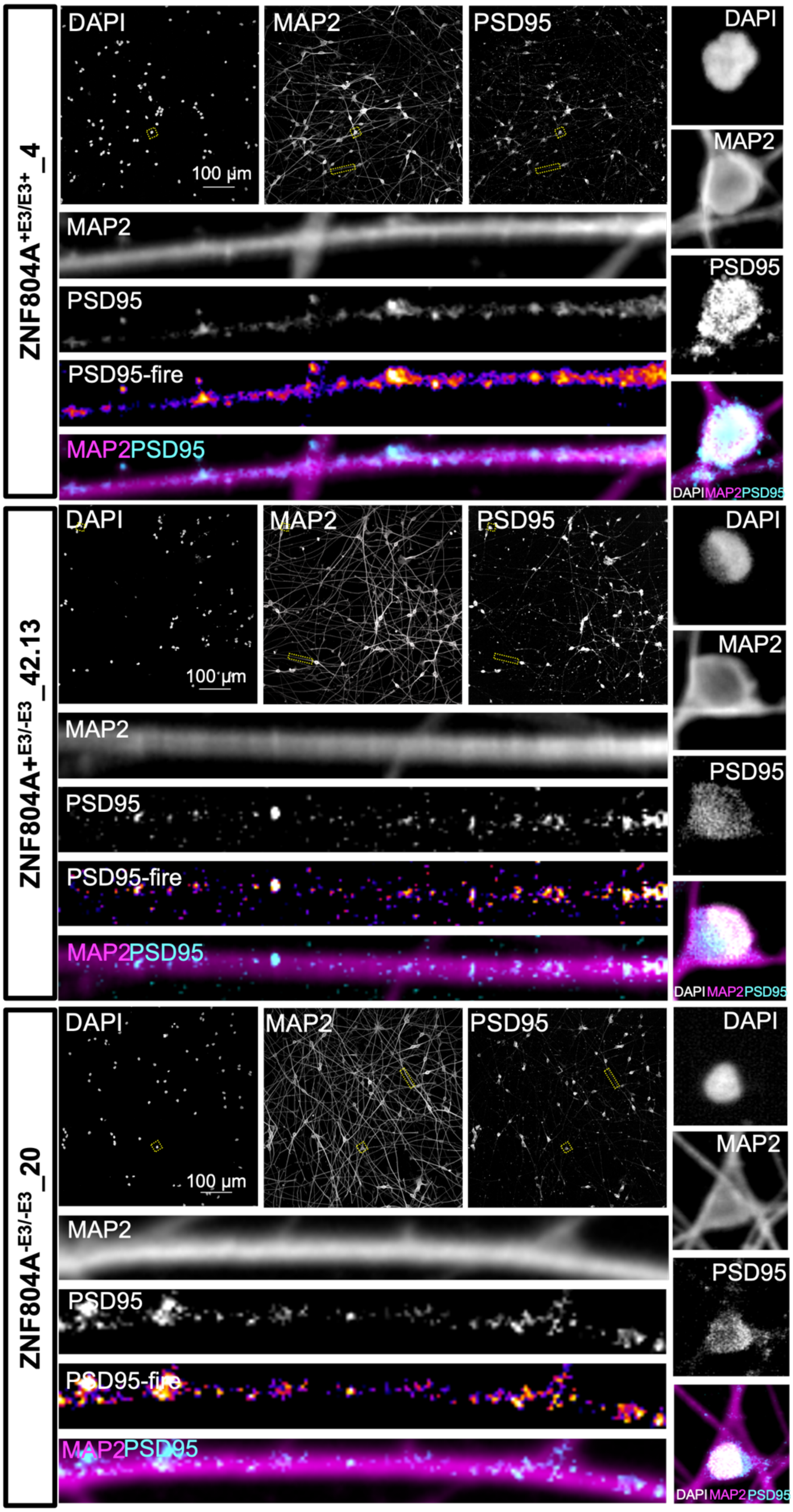
ZNF804A+E3/E3+_4
ZNF804A+E3/-E3_42.13
ZNF804A-E3/-E3_20
DAPI
MAP2
PSD95
100 µm
PSD95-fire
MAP2PSD95
DAPIMAP2PSD95

B

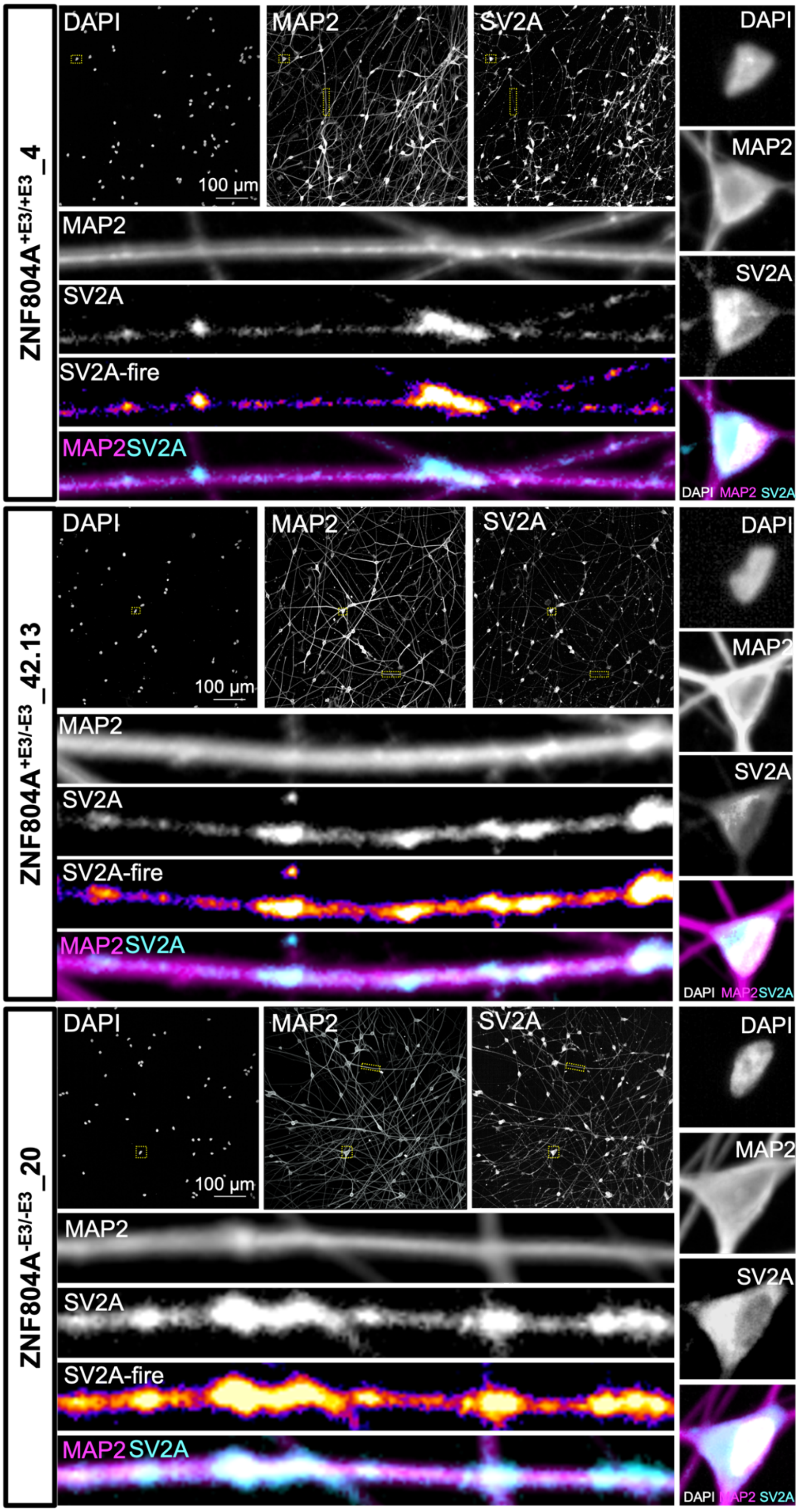
ZNF804A+E3/+E3_4
DAPI
MAP2
SV2A
100 µm
MAP2
SV2A
SV2A-fire
MAP2SV2A
DAPI MAP2 SV2A
ZNF804A+E3/-E3_42.13
DAPI
MAP2
SV2A
100 µm
MAP2
SV2A
SV2A-fire
MAP2SV2A
DAPI MAP2SV2A
ZNF804A-E3/-E3_20
DAPI
MAP2
SV2A
100 µm
MAP2
SV2A
SV2A-fire
MAP2SV2A
DAPI MAP2 SV2A

C

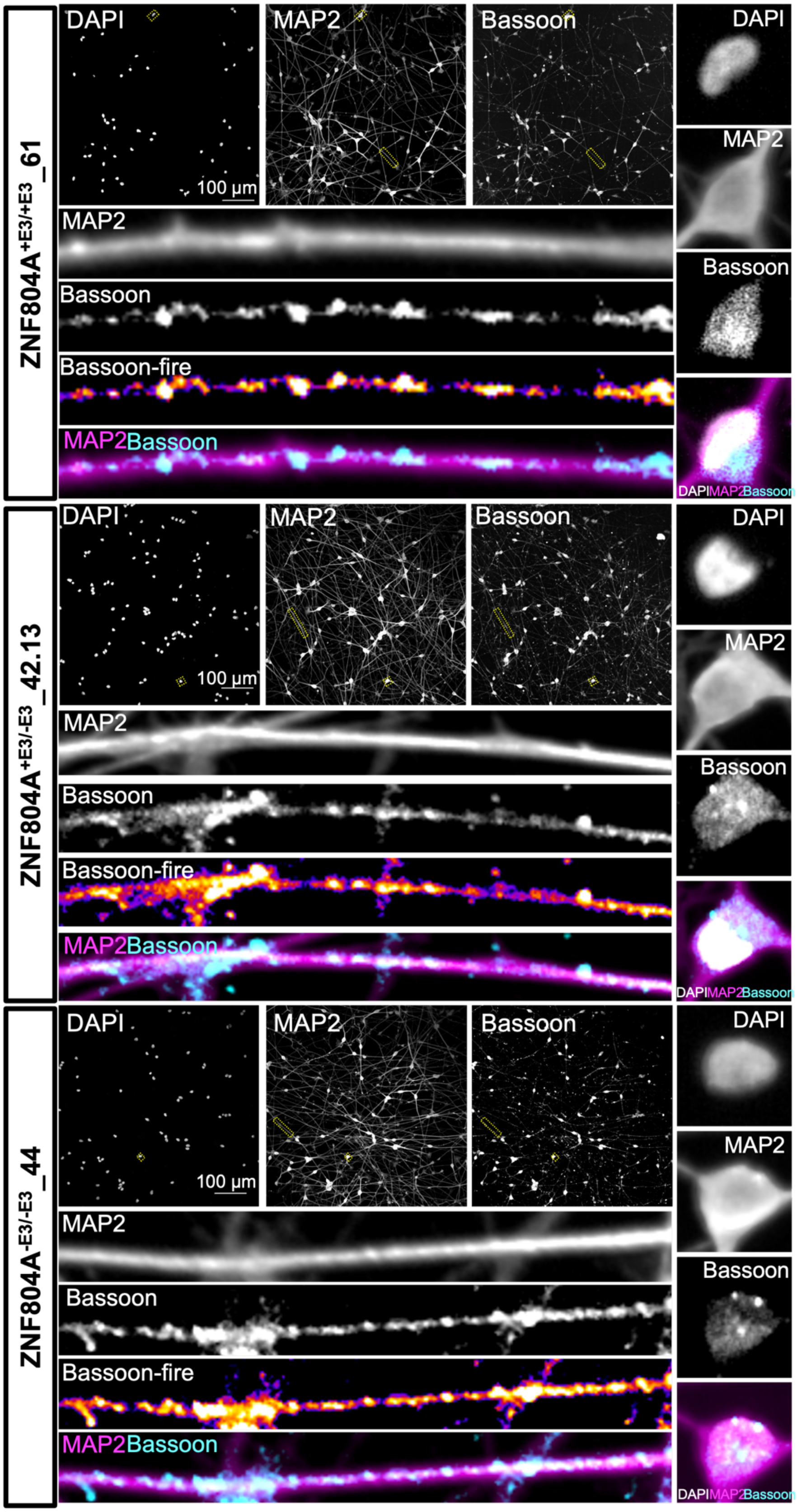
ZNF804A+E3/+E3_61
ZNF804A+E3/-E3_42.13
ZNF804A-E3/-E3_44
DAPI
MAP2
Bassoon
100 µm
Bassoon-fire
MAP2Bassoon
DAPIMAP2Bassoon

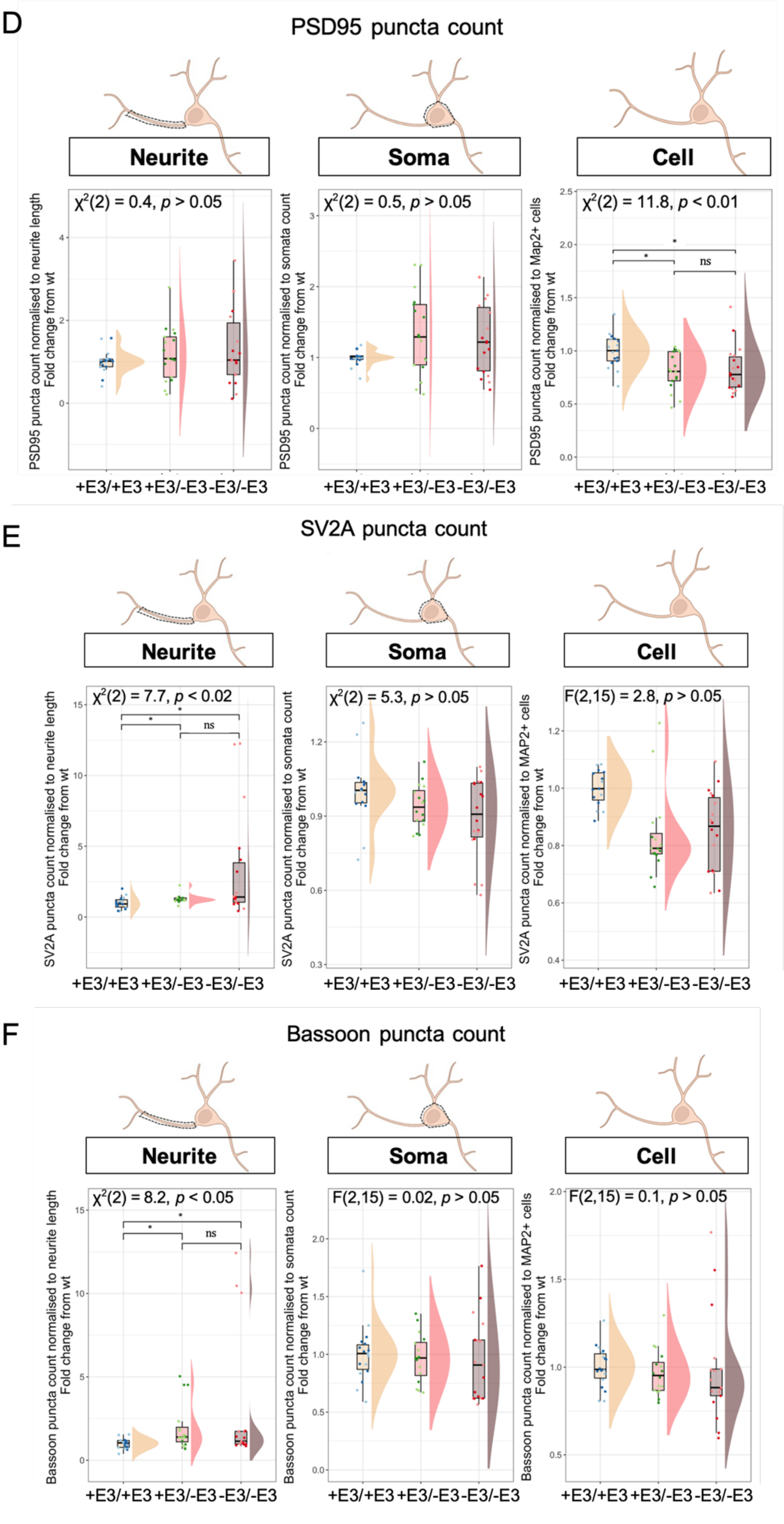

D
PSD95 puncta count
Neurite
Soma
Cell
χ2(2) = 0.4, p > 0.05
χ2(2) = 0.5, p > 0.05
χ2(2) = 11.8, p < 0.01
PSD95 puncta count normalised to neurite length Fold change from wt
PSD95 puncta count normalised to somata count Fold change from wt
PSD95 puncta count normalised to Map2+ cells Fold change from wt
+E3/+E3 +E3/-E3 -E3/-E3
E
SV2A puncta count
Neurite
Soma
Cell
χ2(2) = 7.7, p < 0.02
χ2(2) = 5.3, p > 0.05
F(2,15) = 2.8, p > 0.05
SV2A puncta count normalised to neurite length Fold change from wt
SV2A puncta count normalised to somata count Fold change from wt
SV2A puncta count normalised to MAP2+ cells Fold change from wt
+E3/+E3 +E3/-E3 -E3/-E3
F
Bassoon puncta count
Neurite
Soma
Cell
χ2(2) = 8.2, p < 0.05
F(2,15) = 0.02, p > 0.05
F(2,15) = 0.1, p > 0.05
Bassoon puncta count normalised to neurite length Fold change from wt
Bassoon puncta count normalised to somata count Fold change from wt
Bassoon puncta count normalised to MAP2+ cells Fold change from wt
+E3/+E3 +E3/-E3 -E3/-E3

Fig. S8 Synaptic proteins are increased in and juxtaposed to MAP2$^+$ neurites. Representative confocal images (A - C) of ZNF804A$^{+E3/+E3}$, ZNF804A$^{+E3/-E3}$, and ZNF804A$^{-E3/-E3}$ developing glutamatergic neurons (Day 7) from high-content confocal microscopy. Cells were immunostained for pan-neuronal marker MAP2 (magenta) to identify neurite, somata and whole cell region morphology. An example of post-synaptic density protein 95 (PSD95; A), pre-synaptic synaptic vesicle glycoprotein 2A (SV2A; B) and Bassoon (C) puncta are shown in neurite and somata regions in cyan and fire LUT. (D-F) Quantification of synaptic puncta counts in neurites, somata and whole cell regions of MAP2$^+$ neurons plotted in raincloud plots. Boxplots (left), corresponding density plots (right) and overlaying dotplots of technical replicates for each clones give clear visualisation of data variability. Statistical analysis on averaged technical replicates normalised to neurite length, somata or cell counts and as ratio to the respective wildtype counts acquired from images, which were imaged in the same plate, showed (D) a significant decrease of PSD95 puncta in whole cell regions (Kruskal-Wallis: $\chi^2(2)=11.8$, $p = 0.003$, $n = 18$) in ZNF804A$^{+E3/-E3}$ and ZNF804A$^{-E3/-E3}$ (Dunn's post-hoc tests with holm corrections: $p.adj < 0.05$) neurons, and (E) significant increases of SV2A (Kruskal-Wallis: $\chi^2(2) = 7.7$, $p = 0.02$, $n = 18$) and (F) Bassoon (Kruskal-Wallis: $\chi^2(2) = 8.2$, $p = 0.02$, $n = 18$) puncta lining neurite regions of ZNF804A$^{+E3/-E3}$ and ZNF804A$^{-E3/-E3}$ neurons (Dunn's post-hoc tests with holm corrections: $p.adj. < 0.05$). Created in BioRender. Powell, T. (2026) https://BioRender.com/vh5vr9x.

*p.adj. < 0.05

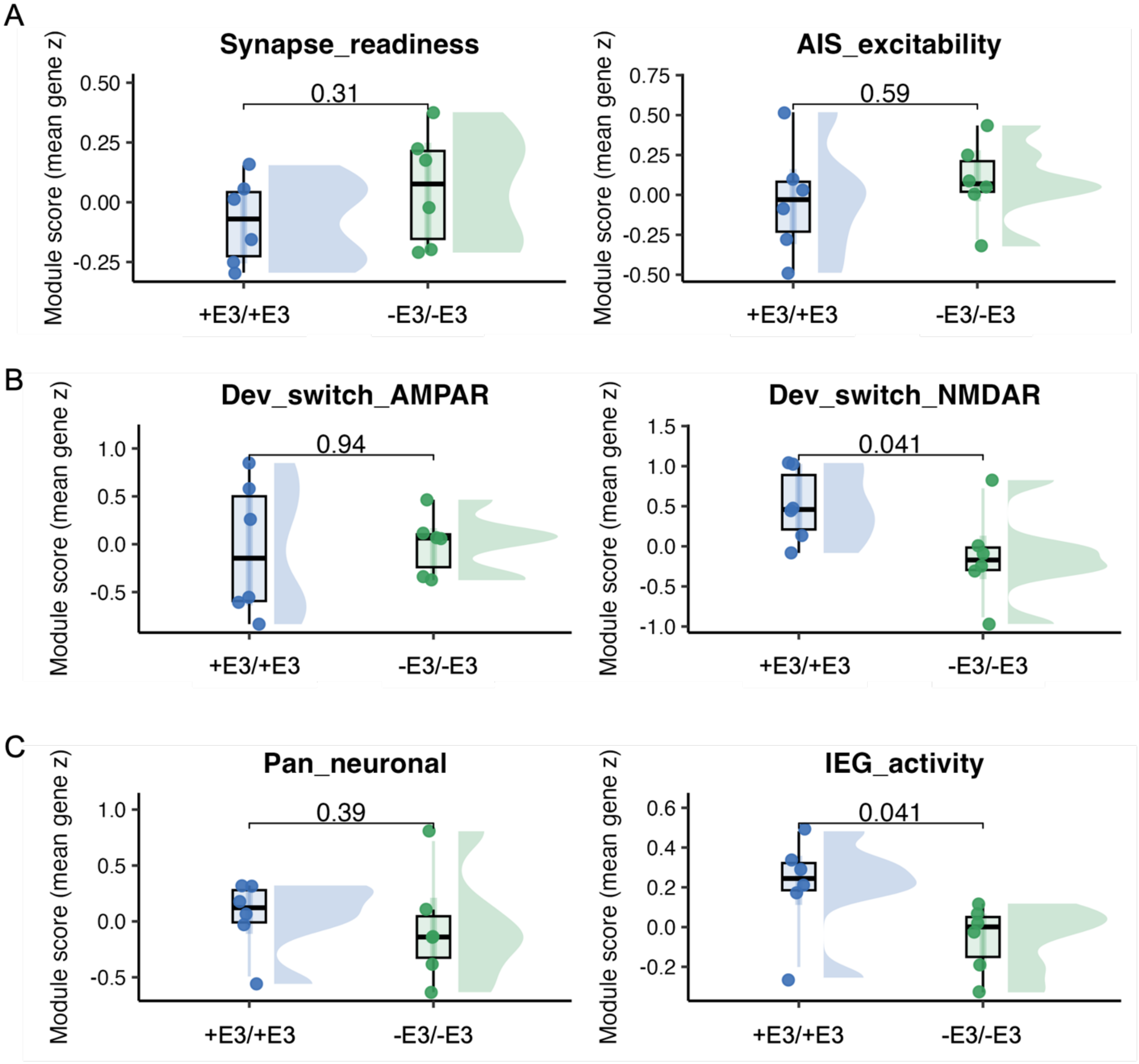

Fig S9 Synapse- and neuronal-related transcriptional programs (A-C) Module-level expression of synaptic, excitability, and activity-related gene programs: (A) Synapse_readiness, AIS_excitability; (B) Dev_switch_AMPAR, Dev_switch_NMDAR; (C) Pan_neuronal, IEG_activity) in ZNF804A$^{+E3/+E3}$, ZNF804A$^{+E3/-E3}$, and ZNF804A$^{-E3/-E3}$ neurons. Module scores represent the mean z-scored expression across genes within each module for individual samples. ZNF804A$^{+E3/+E3}$ vs ZNF804A$^{-E3/-E3}$ comparisons were performed using two-sided Wilcoxon rank-sum tests.

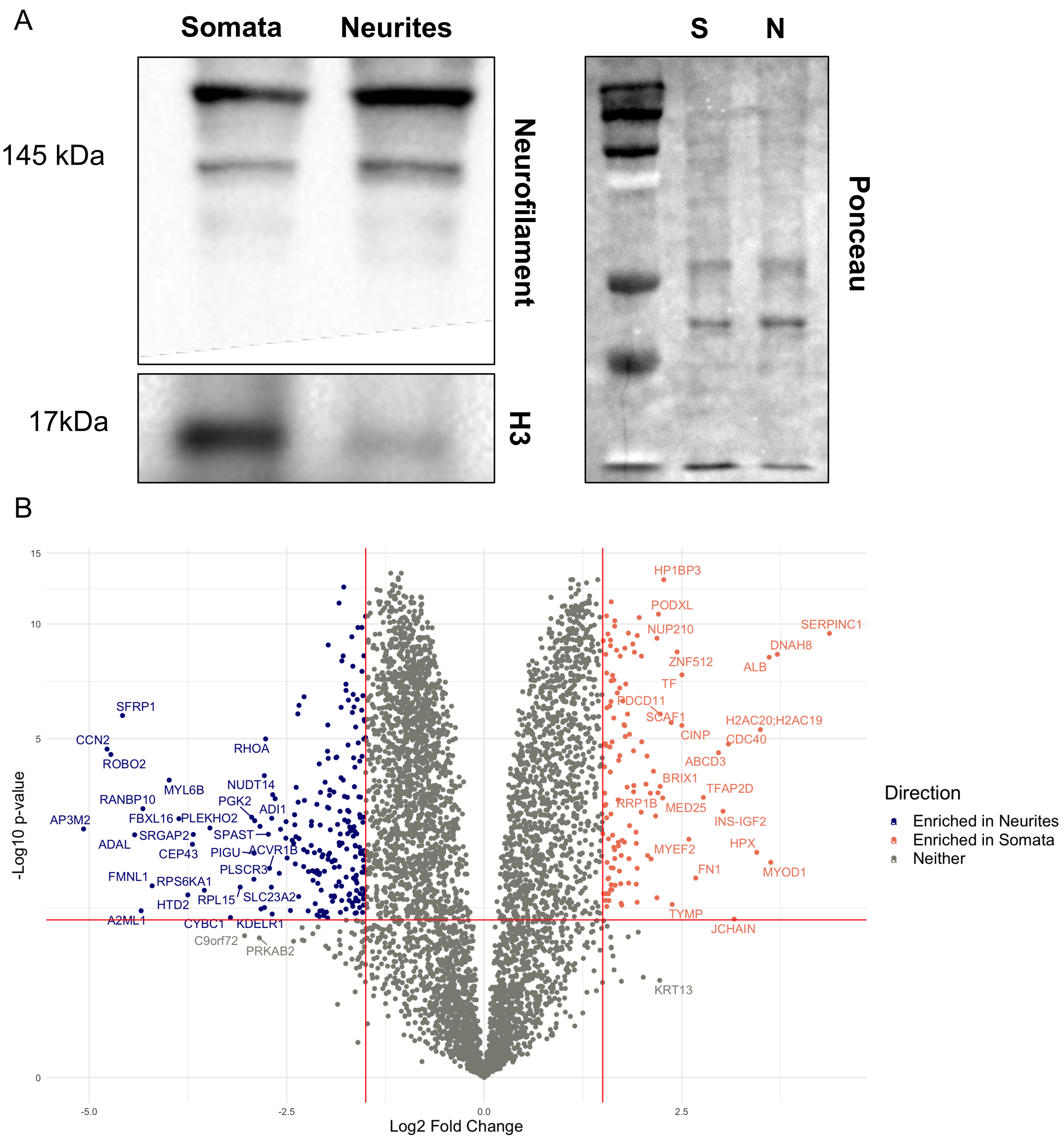

Fig. S10 Validation of compartment separation assay. (A) Representative Western blot showing enrichment of Neurofilament (pan-axonal ~145-160 kDa) in neurite and histone 3 (H3 ~17 kDa) in soma sections. Loading control is shown on the right by ponceau staining. (B) Volcano plot showing enrichment of proteins in neurites and somata. The x-axis represents log2FC, y-axis -log10 p-value. Neurite-enriched proteins

(log2FC < -1.5; FDR < 0.05) are indicated in blue, somata-enriched proteins (log2FC > 1.5; FDR < 0.05) are indicated in red.

A

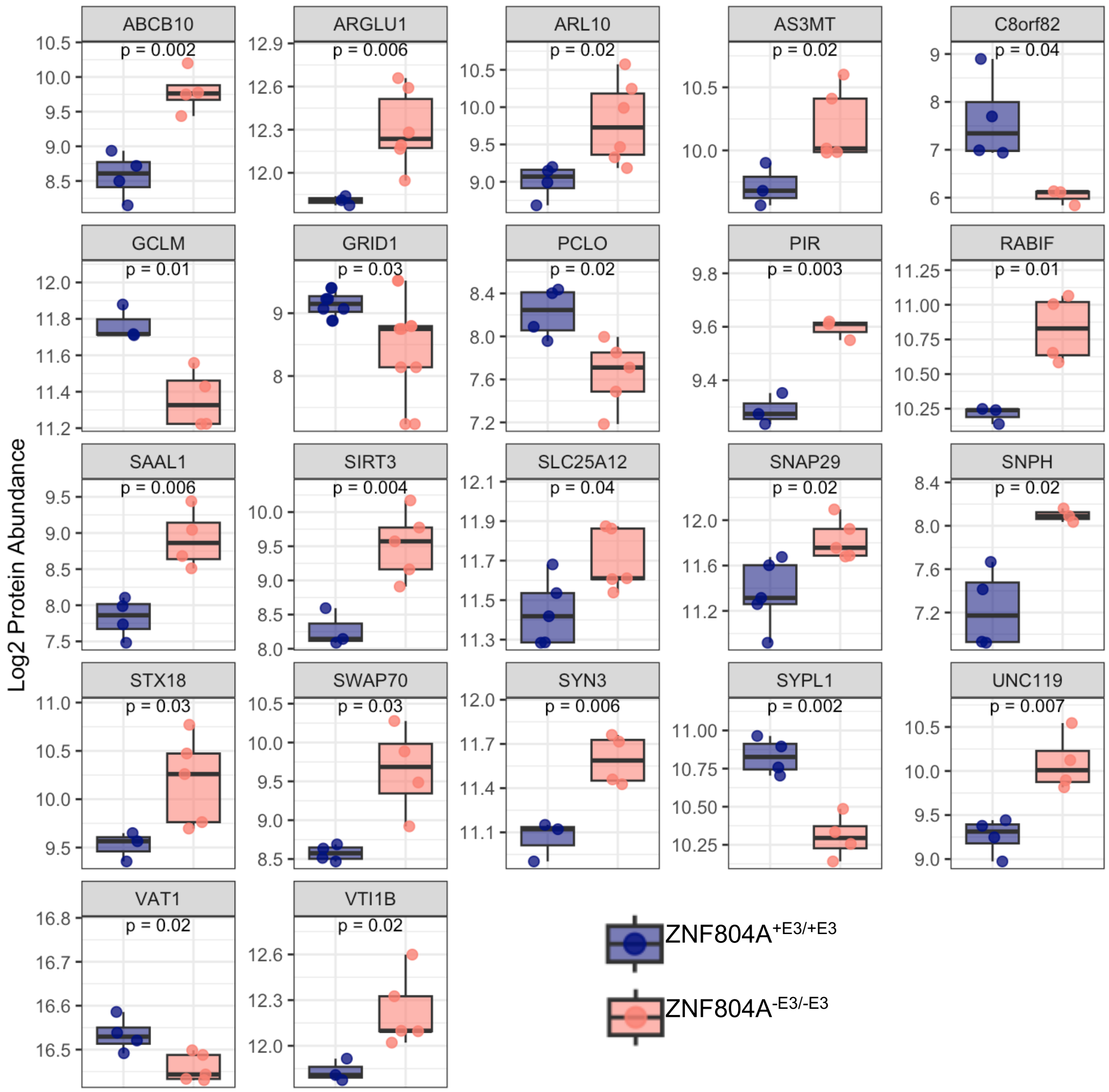
Log2 Protein Abundance
ABCB10
p = 0.002
ARGLU1
p = 0.006
ARL10
p = 0.02
AS3MT
p = 0.02
C8orf82
p = 0.04
GCLM
p = 0.01
GRID1
p = 0.03
PCLO
p = 0.02
PIR
p = 0.003
RABIF
p = 0.01
SAAL1
p = 0.006
SIRT3
p = 0.004
SLC25A12
p = 0.04
SNAP29
p = 0.02
SNPH
p = 0.02
STX18
p = 0.03
SWAP70
p = 0.03
SYN3
p = 0.006
SYPL1
p = 0.002
UNC119
p = 0.007
VAT1
p = 0.02
VTI1B
p = 0.02
ZNF804A+E3/+E3
ZNF804A-E3/-E3

B

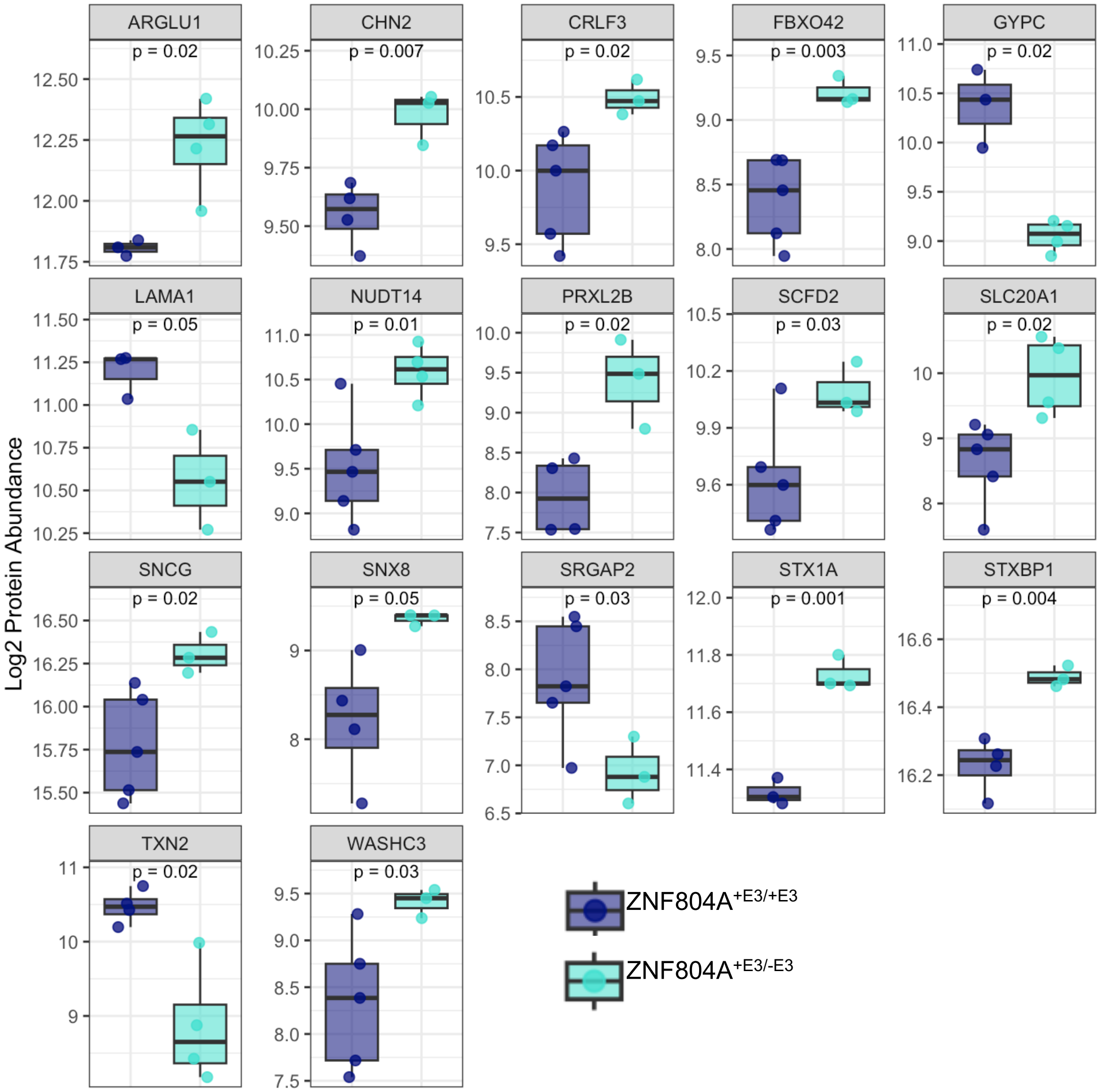

Fig. S11 Compartment-separation assay shows neurite-localized change in synaptic proteins. Boxplots of log2-transformed LFQ values of synaptic proteins expressed within the neurite compartments of wildtype and ZNF804A mutant neurons. Overlaying dotplots show biological replicates. (A) Two-sample student t-test confirmed up-regulation of pre- and post-synaptic proteins in ZNF804A$^{-E3/-E3}$ (e.g. ARGLU1: $t(9) = -3.75$, $p < 0.01$, SNAP29: $t(8) = -3.75$, $p < 0.01$, SYN3: $t(5) = -4.39$, $p < 0.01$, VTI1B: t(6)

= -2.73, $p < 0.05$; A) and (B) ZNF804A$^{+E3/-E3}$ (e.g. ARGLU1: $t(7) = -3.68$, $p < 0.05$, STX1A: $t(4) = -9.39$, $p < 0.001$, STXBP1: $t(5) = -5.17$, $p < 0.01$, SNCG: $t(6) = -3.02$, $p < 0.05$; B) cells compared to ZNF804A$^{+E3/+E3}$ controls.

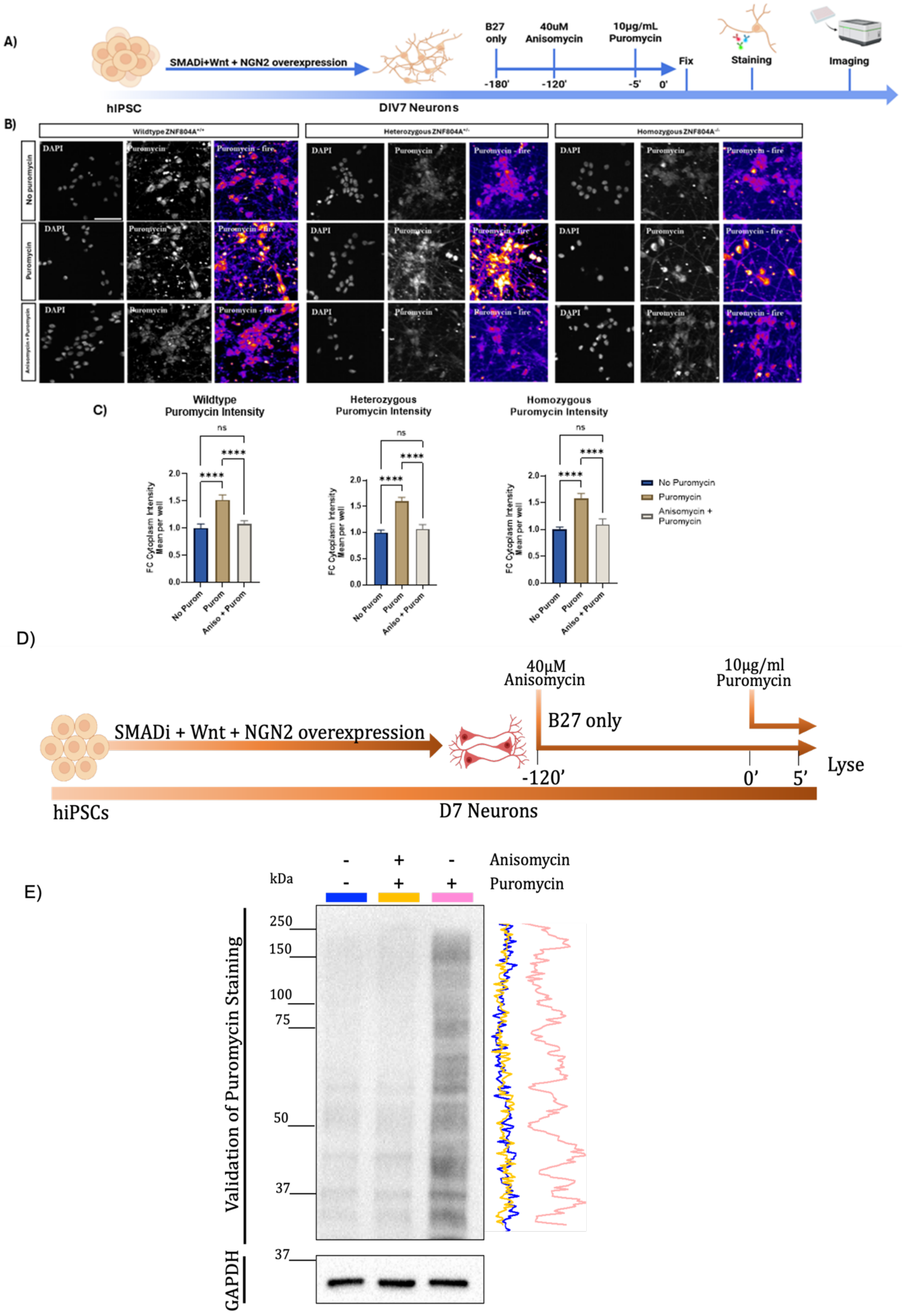
A)
SMADi+Wnt + NGN2 overexpression
B27 only
40uM Anisomycin
10µg/mL Puromycin
-180'
-120'
-5'
0'
Fix
Staining
Imaging
hIPSC
DIV7 Neurons
B)
No puromycin
Puromycin
DAPI
Puromycin
Puromycin - fire
C)
Wildtype Puromycin Intensity
Heterozygous Puromycin Intensity
Homozygous Puromycin Intensity
FC Cytoplasm Intensity Mean per well
ns
****
No Purom
Purom
Aniso + Purom
No Puromycin
Puromycin
Anisomycin + Puromycin
D)
40µM Anisomycin
10µg/ml Puromycin
B27 only
SMADi + Wnt + NGN2 overexpression
-120'
0'
5'
Lyse
hiPSCs
D7 Neurons
E)
Anisomycin
Puromycin
kDa
250
150
100
75
50
37
Validation of Puromycin Staining
GAPDH

Fig. S12 Validation of surface sensing of translation (SUnSET) assay. (A) Experimental timeline of validation of surface sensing of translation (SUnSET) and sample processing by high content imaging. (B) Representative confocal images of puromycin incorporation (fire LUT) in cytoplasm of ZNF804A$^{+E3/+E3}$, ZNF804A$^{+E3/-E3}$, and ZNF804A$^{-E3/-E3}$ developing neurons captured with high-content microscope (scale bar 500 µm). Puromycin incorporation was tested by intensity measurement in presence (row 2) and absence (row 3) of anisomycin, or with not treatment at all (row 1). (C) Bar graphs showing fold changes of puromycin intensity in cytoplasm compared to "No Puromycin" condition. All genotypes show a significant reduction in puromycylated polypeptides when treated with protein synthesis inhibitor, anisomycin, compared to the "Puromycin" only condition (One way ANOVA: ZNF804A+/+ $F(2, 15) = 81.78$, ZNF804A+/- $F(2, 15) = 129.3$, ZNF804A-/- $F(2, 15) = 81.71$, **** = $P < 0.0001$, n=6 wells per condition). (D) Schematic representation of experimental setup. Abbreviations: hiPSCs = human induced pluripotent stem cells, SMADi = SMAD inhibition, NGN2 = neurogenin 2. (E) Representative Western blot showing puromycin incorporation. Incorporation of puromycin was assessed by antibody detection under three conditions: no treatment (lane 1, blank), treatment with 40 µM anisomycin (lane 2), and puromycin alone (lane 3). The line graph on the right displays the staining intensity profile for each lane. Glyceraldehyde-3-phosphate dehydrogenase (GAPDH) serves as a loading control. Puromycin incorporation is undetectable in the untreated (blank) and anisomycin-treated samples. Created in BioRender. Powell, T. (2026) https://BioRender.com/vh5vr9x.

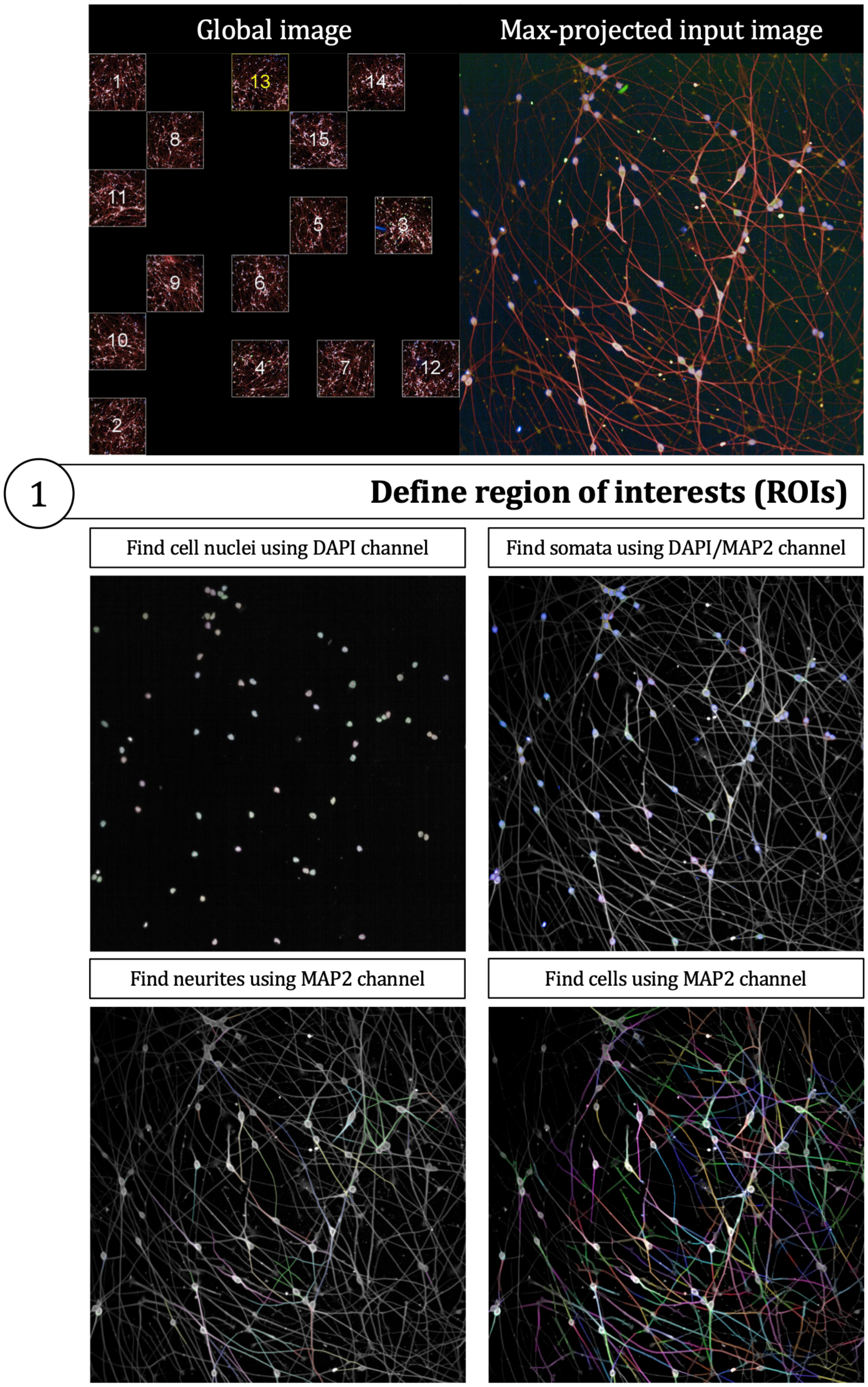
Global image
Max-projected input image
1
Define region of interests (ROIs)
Find cell nuclei using DAPI channel
Find somata using DAPI/MAP2 channel
Find neurites using MAP2 channel
Find cells using MAP2 channel

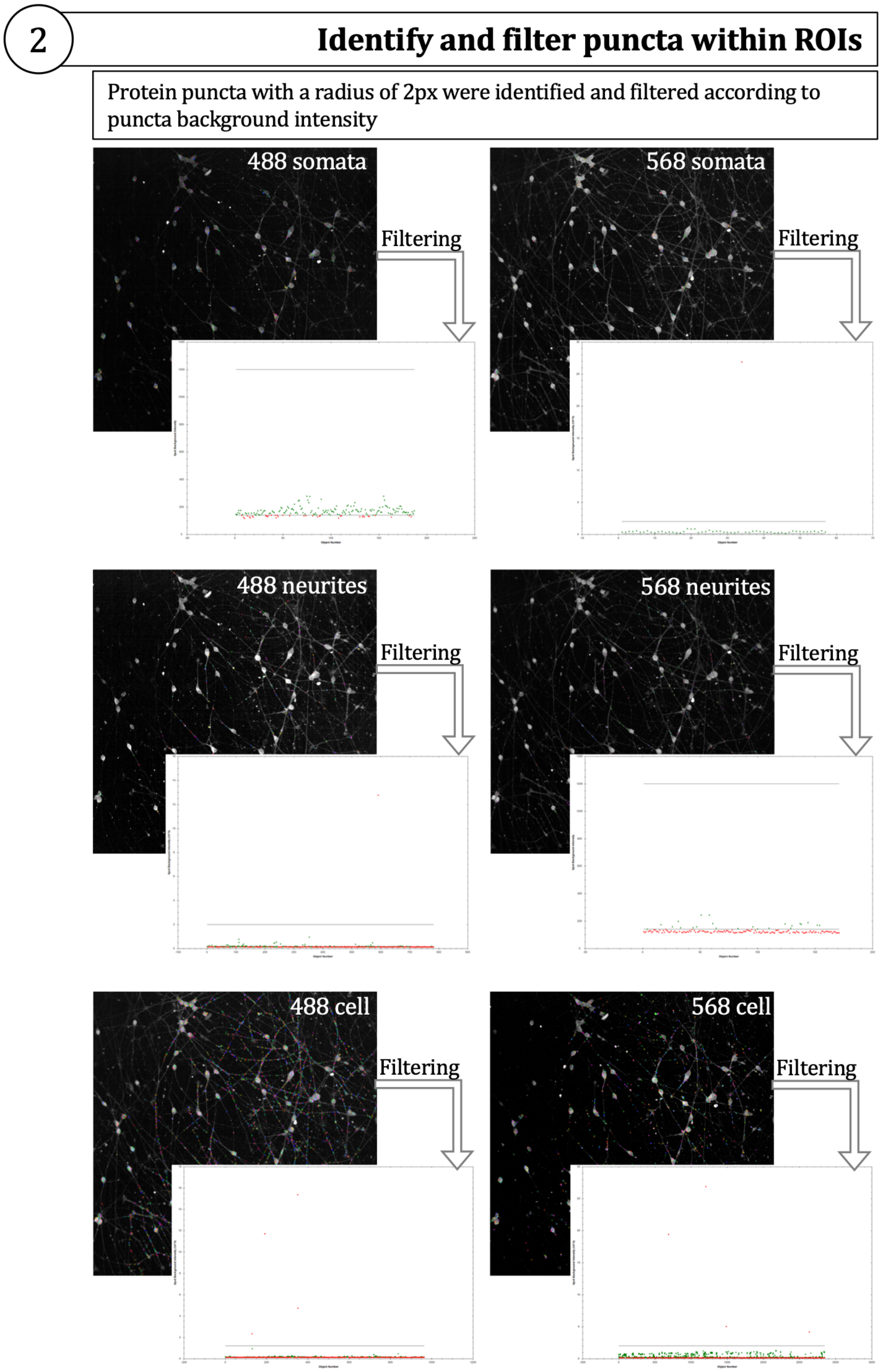

Fig. S13 Exemplary diagram of automated analysis pipeline. Automated analysis pipeline was instructed to (i) define regions of interests (ROIs) and (ii) detect and filter puncta within ROIs. DAPI and MAP2 channels were basis of somata identification, MAP2

channels helped to outline cells and identify neurites. Puncta detection was based on radii not exceeding 5 pixels (px) (~1.5μM) within predefined ROIs. Filtering of detected puncta was based on “Spot Background Intensity”. Further analyses were conducted only on puncta that met the filtering criteria.

Table Legends

Table S1. List of Primers and Antibodies used in this study. Forward and reverse primer sequences including amplicon sizes ordered by experiments, for which they were used in this study.

Table S2. Results of statistical analysis for Fig. 1B - C. Summary results table (top) of two-way ANOVA for isoform, timepoint, line and isoform:timepoint interaction effects on *ZNF804A* expression in neuroprogenitor cells (NPCs). Results table of Tukey's test for post-hoc analysis (bottom) with difference in means (*.diff), confidence levels (*.lwr, *.upr) and the adjusted p-values for all possible pairs for each tested variable (isoform, line and timepoint).

Table S3. RNASeq: Differentially expressed genes (DEGs) of Signatures A, B and C.

Table S4. Gene ontology (GO) terms and statistics for differentially expressed genes (FDR 7%) in ZNF804A-E3/-E3 neurons. Green colour-coding identifies terms plotted in dotplot (FDR < 0.05).

Table S5. List of genes used to build gene modules specific for calcium signalling, synapses, AIS, pan-neuronal and IEG modules.

Table S6. Log2-transformed label free quantitation (LFQ) scores for 5393 identified proteins in each sample

Table S7. Results of differential protein expression analysis according to cellular compartment**.**

Table S8. Gene ontology (GO) terms and statistics for proteins enriched in neurites (top) and somata (bottom).

Table S9. Cell line information.

Table S10. Single guide RNA (sgRNA) sequences used for dual single-guide RNA CRISPR/Cas9 approach including on- and off-target scores provided by IDT or CRISPOR.

Table S11. RNA sequencing quality scores and number of unique reads for each sample.

Table S12. List of antibodies used in this study.
